# Structural inequalities exacerbate infection disparities

**DOI:** 10.1038/s41598-025-91008-w

**Published:** 2025-03-17

**Authors:** Sina Sajjadi, Pourya Toranj Simin, Mehrzad Shadmangohar, Basak Taraktas, Ulya Bayram, Maria V. Ruiz-Blondet, Fariba Karimi

**Affiliations:** 1 Complexity Science Hub, Vienna, Austria; 2 IT:U Interdisciplinary Transformation University Austria, Linz, Austria; 3 Central European University, Vienna, Austria; 4 INSERM, Institut Pierre Louis d’Epidémiologie et de Santé Publique, Sorbonne Université, Paris, France; 5 Shahid Beheshti University, Tehran, Iran; 6 Bogazici University, Istanbul, Turkey; 7 Çanakkale Onsekiz Mart University, Çanakkale, Turkey; 8 Neurable, Boston, USA; 9 Graz University of Technology, Graz, Austria

**Keywords:** Physics, Information theory and computation, Statistical physics, thermodynamics and nonlinear dynamics

## Abstract

During the COVID-19 pandemic, the world witnessed a disproportionate infection rate among marginalized and low-income groups. Despite empirical evidence suggesting that structural inequalities in society contribute to health disparities, there has been little attempt to offer a computational and theoretical explanation to establish its plausibility and quantitative impact. Here, we focus on two aspects of structural inequalities: wealth inequality and social segregation. Our computational model demonstrates that (a) due to the inequality in self-quarantine ability, the infection gap widens between the low-income and high-income groups, and the overall infected cases increase, (b) social segregation between different socioeconomic status (SES) groups intensifies the disease spreading rates, and (c) the second wave of infection can emerge due to a false sense of safety among the medium and high SES groups. By performing two data-driven analyses, one on the empirical network and economic data of 404 metropolitan areas of the United States and one on the daily Covid-19 data of the City of Chicago, we verify that higher segregation leads to an increase in the overall infection cases and higher infection inequality across different ethnic/socioeconomic groups. These findings together demonstrate that reducing structural inequalities not only helps decrease health disparities but also reduces the spread of infectious diseases overall.

## Introduction

The COVID-19 pandemic resulted in a disproportionate infection rate among marginalized groups, people of color^1^, and low-income groups^2,3^. Health inequalities are often due to existing structural inequalities in which societies foster discrimination through mutually reinforcing systems such as housing, education, employment, and health care^4^. It has long been established that pandemics expose and deepen existing racial, ethnic, and income inequalities^5^ in society by aggravating resource constraints and rendering living conditions direr for those who live on the margins^6^. Despite growing interest in understanding how social and structural factors drive inequalities in health outcomes, systematic and quantitative understanding of their effects as a root cause of health inequalities has not received much attention^7^.

Inspired by the recent COVID-19 outbreak, this paper studies the effect of structural inequalities, namely income inequality and social segregation, on the infection disparities among different socioeconomic groups during public health crises. We propose a novel explanatory framework that incorporates game theory, agent-based modeling, and network analysis, to shed light on the impact of individual decision-making dynamics on the macro-level epidemic outcomes.

In this paper, we investigate two crucial factors contributing to structural inequalities, and subsequently, health inequalities^8^: (1) Income inequalities, and (2) Social segregation. Income inequalities often manifest in socioeconomic status and correlate with race and ethnicity, influencing multiple aspects of human life and decision-making processes. Research suggests that income inequality and marginalization increase infection rates among disadvantaged groups due to the inability to reduce mobility^9–12^. Social segregation is commonly associated with the homophilic tendencies to interact and associate with similar others^13^ along the racial^14,15^, ethnicity^16^, or occupational lines^17^. This social tendency at the micro-level influences the social network structure at the macro-level^18^ and impacts the spreading dynamics^19–22^. Evidence suggests that income inequalities and social segregation generate persistent long-term health inequalities between upper- and lower-income households^23,24^, which we must take into account to make realistic predictions on epidemic diffusion patterns.

During public health crises, social distancing and confinement measures become of utmost priority to contain the spread of the infection. However, the level of compliance with these measures is closely related to the nature of working and living conditions. Crucially, lower-income groups experience greater income loss due to the economic slowdown that lockdown and social distancing measures generate, which, in turn, may depress these groups’ willingness to stay in and wait for the pandemic out. Thus it is important to consider the interplay between epidemic growth, income status, and the individuals’ willingness to self-quarantine^1^ over time.

Existing empirical studies tend to focus on structural determinants of health inequality^26–29^, and long-term health implications of such inequality^30,31^ and unequal access to vaccination^32^. The few studies that addressed the decision-making dynamics (such as vaccination games) did not elaborate on the segregation aspect^33–35^.

To this end, we study the effect of socioeconomic disparities and social segregation on infection and subsequently mortality rates using epidemiological models. We compare the behavior of higher-income groups to that of lower-income groups when governments enforce lockdown measures.

This modeling framework effectively addresses several limitations found in the previous simulation models of epidemics. First, while an extensive body of research has been focused on the dynamics of the underlying social contacts^36–40^, The majority of the existing works tend to focus solely on the contact dynamics as an independent process, while overlooking the impact of infection-induced factors. Second, existing computational studies of epidemics, including COVID-19 cases, use Susceptible-Infectious-Removed (SIR) models to calculate the efficiency^41^ and practicality of herd immunity strategies^42,43^. However, these studies do not consider the feasibility of adopting public health strategies among individuals with varying levels of adaptability to the new policies. They also ignore social segregation as one of the drivers of disparity in infection cases among different socioeconomic groups. In reality, not only are individuals more likely to interact with those in their vicinity but also their level of compliance with lockdown measures varies by their socioeconomic status. Third, while game theoretical models are effective in modeling trade-offs between optimal vaccination strategies and economic costs^44^, as well as the effectiveness of social distancing and individual decisions to comply with lockdown measures^45,46^, these models often do not incorporate social segregation in investigating diffusion dynamics^47^. Here we combine the strengths of these approaches, by embedding an adaptive game-theoretical model (to capture decision making with trade-offs) into an agent-based network model (that examines the effect of network variations in diffusion).

Our numerical, analytical, and empirical analysis confirms that the adverse effect of income inequality and social segregation get amplified during health crises, especially when governments enforce confinement measures to contain the spread of infection. Reducing structural inequalities not only helps in decreasing health disparities but also reduces the spread of infectious diseases overall.

## Results

### Computational framework

We consider the spreading of an infection in a segregated society, where people (agents) need to decide whether to self-quarantine themselves or not as an adaptive dynamical process. The population is classified into socioeconomic (SES) groups/blocks, with the total number of groups denoted by *B*. Group membership also controls the interaction patterns of the agents, as there is a higher interaction probability *within* the groups, compared to the interaction probability *across* different groups. Thus, our model consists of three components: (a) contact network, (b) decision-making process, and (c) epidemic spreading. The components are described as follows in the following subsections. We also provide a mean-field approximation of this agent-based model (*See section*S2*in the Supplementary Information for the detailed derivation.*)

#### Contact network

We use the Stochastic Block Model (SBM) to model the segregation between different communities. Every two agents *i* and *j* respectively belonging to socioeconomic groups/blocks *a* and *b* are connected with a probability $\rho _{a,b}$, where $\varvec{ \rho }$ is known as the connection probability matrix. Due to the residential segregation, the probability of interaction among individuals of the same group is higher than average. Parameter *s* controls the level of segregation, with $s=0$ and $s=1$, leading to fully homogeneous and fully segregated communities respectively (see the Supplementary Information S3). We acknowledge that our method is mainly suitable for modeling residential segregation^48,49^. However, segregation exists in other forms, such as the workplace segregation^50,51^ and mobility segregation^52^ which can be the focus of future works.

#### Decision making

At each time step, every agent decides whether to *quarantine* itself or to *participate* in the society, i.e., go to work. Following^53–56^ we use the Fermi decision-making function to model the decision-making process, where agents assign probability $e ^ {\beta r_{\omega }} / C$ to each option $\omega$, based on its possible reward/punishment *r*. *C* serves as the normalization factor and $\beta$ indicates the intensity of selection. For $\beta \rightarrow \infty$ agents will almost definitely pick the option with the slightest reward advantage, while for $\beta \rightarrow 0$ they randomly choose; disregarding the rewards. For smaller positive $\beta$ values, agents assign a higher probability to the option with the higher reward.

Participation or going to work, exposes the agent to the infection, resulting in a *psychological fear of infection punishment*. The option of quarantining, in contrast, leads to *income loss*. We model the punishment of participation as $r_d I(t)$. Where $r_d$ denotes the baseline fear of infection and *I*(*t*) is the total fraction of infectious agents at time *t*. Hence, the perceived risk of infection is linearly dependent on the number of infectious agents in the network. Since *I*(*t*) is the *global* proportion of the infectious population, the information processed by the agents would be a global one. In future works, it would be interesting to consider partial information access for the agents.

On the other hand, $r_b$ indicates the income-loss punishment for agents in block *b*. The effect of income loss is conceived to be higher for individuals from lower socioeconomic classes, as they will be less likely to afford to lose their income and quarantine themselves. Hence, we hypothesize the punishment of income loss to be inversely proportional to the wealth of each individual ($r_b = -\frac{1}{w_b}$). Wealth *w* is distributed by the Pareto distribution $p(w) = \frac{\lambda w_m^\lambda }{w^{\lambda +1}}$^57,58^. The parameter $\lambda$ controls the level of equality, where $\lambda \rightarrow 0$ and $\lambda \rightarrow \infty$ represent the ultimate inequality (Dirac delta distribution of wealth) and equality (Uniform distribution of wealth) respectively. Distribution of wealth in turn controls the fear of income loss ($r_b = -\frac{1}{w}$) (*See the Supplementary Information.*)

We thus, derive the probability $P_b$ of participation for agents of block *b* to be:

1 $$\begin{aligned} P_b(t) = \frac{1}{e^{\beta \left( r_b - r_d I(t)\right) } + 1 } \end{aligned}$$

We take note that this dynamics only represents the self-imposed quarantine. Top-down confinement measures are out of the scope of this paper but are interesting to incorporate in future works. (See the discussion in S5)

**Fig. 1:**
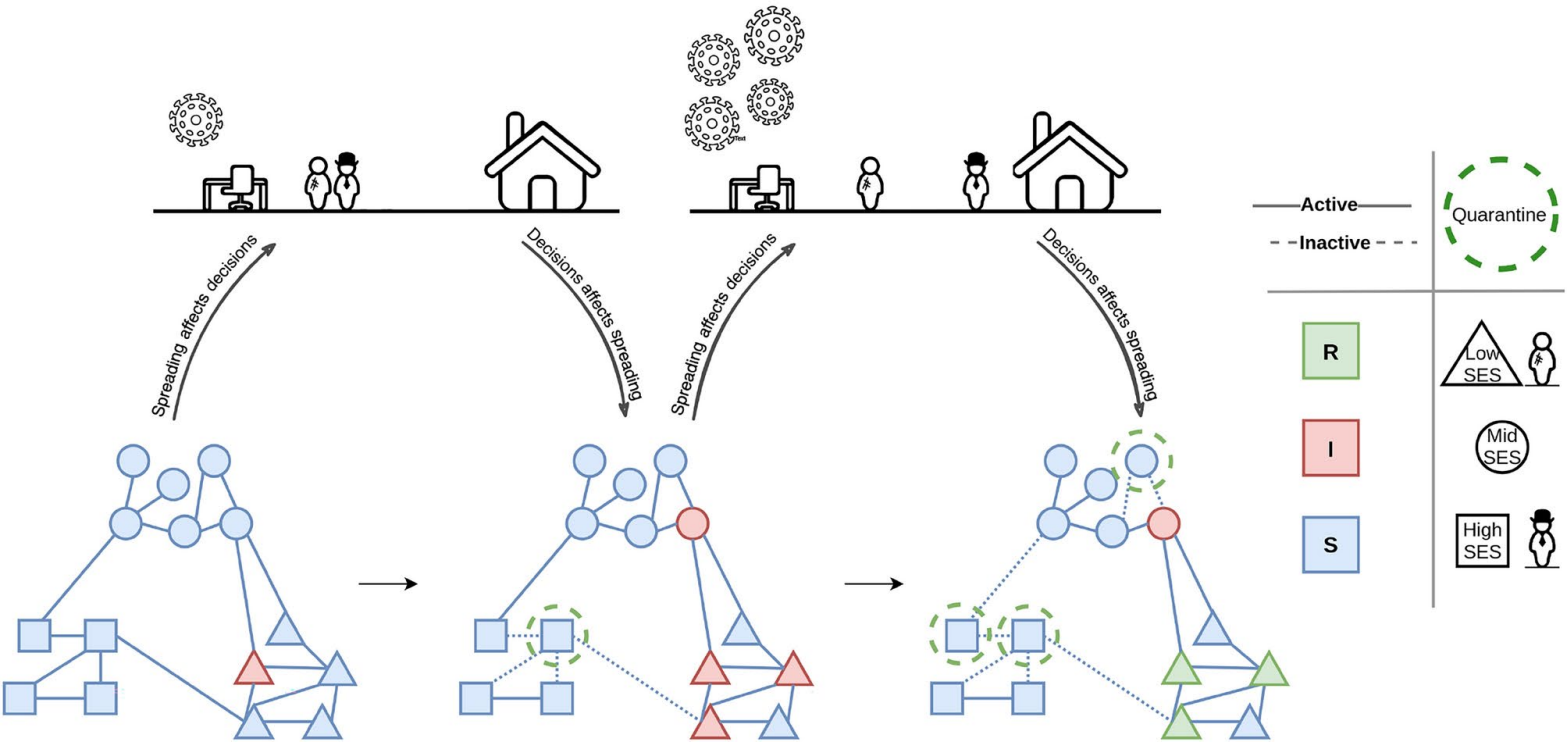
Schematic illustration of the interplay between the spreading and the decision-making models. On the top, we have shown the tendency of high and low SES individuals toward *participation* (Office) and *quarantine* (Home) under low and high infection scenarios. At the bottom we have shown the progress of the dynamics in three snapshots: Infectious agents transmit the disease to their neighbors. Some agents respond by quarantining (dashed green circle) and temporarily detaching themselves from their neighbors, eliminating the transmission possibility. Infectious agents get removed after some time. Shapes denote SES class/block and colors indicate infection status. Solid and dashed lines respectively illustrate the active links, capable of transmitting the disease, and inactive links, incapable of doing so. The curved arrows denote the interplay between the two dynamics resulting in the adaptive dynamics of our model.

**Fig. 2:**
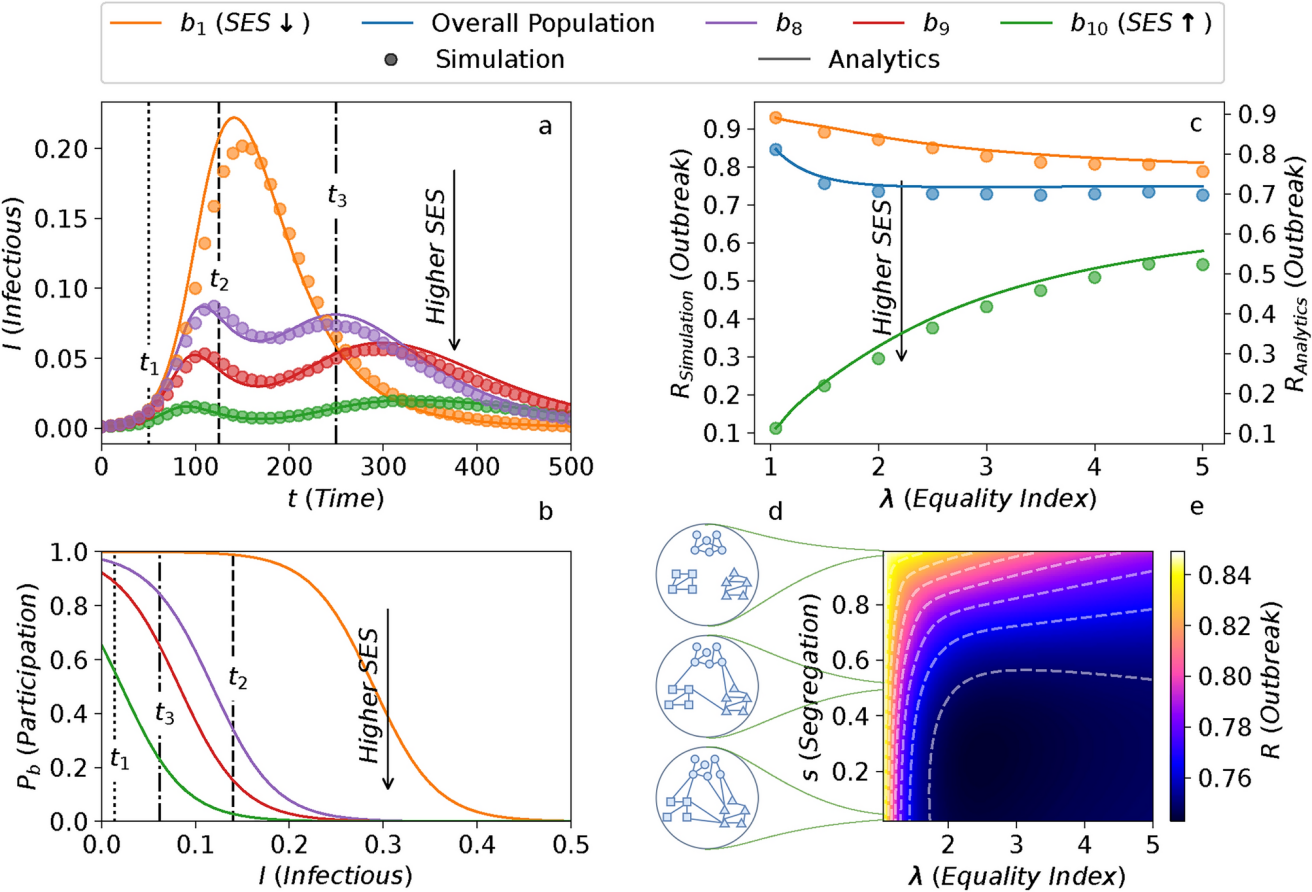
Infection prevalence over time and the final outbreak size. (**a**) Evolution of *I*(*t*), the proportion of infectious agents in each block over time *t*. Color code denotes SES groups, where higher SES groups are assigned to higher indices. (**b**) Probability of participation, $P_b$ (Eq. 1) for each block *b* as a function of the proportion of infectious population *I*. The dotted, dashed and dash-dotted vertical lines indicating the characteristic times $t_1$, $t_2$, and $t_3$ are illustrated on (**a**). The lines denote their corresponding *I*(*t*) values on (**b**). Colors denote the corresponding SES groups in (**a**). (**c**) The outbreak size, *R*, in each block, for varying levels of wealth equality index $\lambda$. Color code denotes SES groups, with higher SES being indexed by higher values. $\overline{d}$ represents the average over all SES groups. The left and right y-axes respectively indicate MFA and ABM; please note the minor discrepancy in their values. (**d**) Schematic network illustrations, for varying values of segregation. The number of intra-block and inter-block links respectively increase and decrease with *s*. (**e**) The overall outbreak size, *R* denoted by the color-axis, for varying levels of *s* and $\lambda$ based on the MFA. The horizontal lines connect the values of *s* to their corresponding networks, in (**c**). The values illustrated in the red window are also represented as the blue curve of (**c**). In (**a** and **c**), the dots and the lines respectively represent the ABM (agent-based model) and the MFA (mean-field approximation) results. *Error bars are smaller than the marker size.*.

#### Epidemic spreading

We use a SIR model to simulate the spread of an infectious disease in the network. We categorize the population into three compartments, *S* (Susceptible), *I* (Infectious), and *R* (Removed). It should be noted that the removed component, *R*, includes both the recovered and the deceased individuals, as both are incapable of further interacting with the disease. The proportion of the deceased individuals within the removed population can be subsequently calculated based on the specific disease, and need not be explicitly considered in the model. Initially a fraction $I_{init}$ of agents in each block are in the state *I*, with the rest being in the state *S*. Infection only propagates among the agents out of quarantine. At each time step, infectious agents *I* infect their *S* neighbors with probability $\mu \Delta t$, where $\mu$ is the transmission rate and $\Delta t$ is the size of a time step. Infectious agents are removed with the removal probability $\gamma \Delta t$, where $\gamma$ is the removal rate, and they will not be able to get infected or infect others again.

Figure 1 illustrates the interplay between the disease-spreading dynamic, the decision-making process of various socioeconomic groups, and social network segregation.

We acknowledge that the SIR model is one of the most simplistic spreading models. However, we will show that this model is capable of exhibiting complex behavior, describing empirical data, and providing us with insight into infection inequalities. We should note that our model focuses on the onset of a new pandemic, before the existence of a vaccine or drastic top-down confinement measures. Hence, in such a time scale, generalizations such as the consideration of demographic changes, birth-death dynamics, or movement between the SES groups are not crucial.

In the supplementary information, we consider more complex scenarios, such as the existence of an incubation period or the possibility of the waning of immunity^59^ (S6) and show that the results are robust with respect to these generalizations.

### The spread of infectious disease over the SES groups

Unless stated otherwise, we use a set of baseline parameters (described in Table S1) for all the analyses conducted in this section. To check the robustness of the calculations we also vary the values of equality index $\lambda$, segregation *s*, and transmission reproduction rate $R_0$. Where $R_0=\frac{\mu {\langle {k}\rangle } }{\gamma }$ controls the infection rate, with ${\langle {k}\rangle }$ describing the average contacts of an agent.

We consider 10 equally populated SES blocks and numerical results are averaged over 100 realizations. We evaluate the numerical results with analytical approximations detailed in the Supplementary Information S2.

Figure 2a shows the evolution of the fraction of infectious individuals in each SES group $I_b(t)$ over time. Panel (b) illustrates the probability of participation for the selected blocks ($P_b$), as a function of infection at time *t* (*I*(*t*)). Initially, at $t = t_1$ the small proportion of infectious people leads to a high probability of diffusion in all groups, hence the infection spreads quickly in all blocks. As the lower SES groups (orange) have a low opportunity of quarantining themselves, they continue participating in daily life. Their infection rates, therefore, increase similarly to the baseline SIR model^60^. It peaks at $t=t_2$ and then at $t=t_3$, as there are not enough susceptible agents remaining, it drops. In other words, they transmit the disease until they achieve the state of herd immunity. On the other hand, the higher SES groups react to the high proportion of infectious cases $(t=t_2)$ and start quarantining at a higher rate, leading to a drastic decrease in their infectious rates. As the lower SES groups achieve herd immunity and the number of active infectious cases drops ($t = t_3$), the higher SES having a *false sense of safety* move out of quarantine, giving rise to a second peak of the pandemic.

We observe a strong agreement between the results of the simulation and mean-field analytical approximation. The slight overestimation in the mean-field is only to be expected, as mean-field approximations generally display faster spreading dynamics. This is for the most part, due to the negligence of the network clustering in the mean-field approximation and reducing the chance of the dynamics getting trapped in closely knitted neighborhoods^59^.

### Role of wealth inequality

Next, we investigate the role of wealth distribution in the epidemic dynamics. In our model, $\lambda$ represents wealth equality in society. As illustrated in section S4 in the Supplementary Information, $\lambda$ affects the quarantine decision-making probabilities $P_b$. Increasing $\lambda$ (more equal regimes) leads to more homogeneous quarantining probabilities across the different SES blocks. Figure 2c illustrates the outbreak size as a function of equality and SES blocks. In the presence of high wealth inequality, the outbreak size is the highest for the lowest SES (orange) and the average population (blue). By increasing $\lambda$ and providing a more equal opportunity for quarantining, the infection prevalence (manifested in *R*) will increase for the higher SES groups. However, the lower SES groups, and most importantly, the overall population will benefit from a significant drop in their infected cases. Therefore, society would profit from a more equal distribution of wealth and consequently a more equal affordability of quarantining.

### Role of social segregation

We next analyze how segregation affects spreading dynamics. As shown in Fig. 2d, the parameter *s* indicates the degree of segregation in the contact network. It ranges from $s = 0$, representing a fully homogeneous network, to $s = 1$, which corresponds to a fully segregated network, where only agents within the same group can connect. The networks are generated using stochastic block models (SBMs), with details provided in section S3 of the Supplementary Information. To separate the effect of segregation, we ensure that the average degree, $\langle k \rangle$, or network density, remains constant across networks with different *s* values.

We perform the same analysis conducted in section "Results", this time also varying the level of segregation *s*. The results of this analysis are illustrated in Fig. 2d, where we demonstrate the average proportion of the outbreak size for different values of segregation *s* and equality index $\lambda$. As it is evident, keeping $\lambda$ constant and increasing *s* (moving vertically towards the top of the plot), significantly increases the outbreak size. This result can be counter-intuitive as higher values of segregation indicate a lower interaction rate between different groups and this might imply the confinement of the disease in the segregated blocks. However, high segregation yields several neighborhoods with a high concentration of agents having low quarantine capability, and hence a high infection risk. The high proximity of these agents can give rise to an explosion of infection throughout the neighborhood, boosting the spread of the disease in society. On the other hand, in a more uniform contact network, the quarantine performed by the high SES individuals, can also protect their low SES neighbors.

### Empirical data analysis

Similar to how we used this methodology to assess infection disparities across SES groups, the method can be applied to study the same disparities across ethnic groups.

In this section, we inform our computational framework by the empirical network and economic data of metropolitan areas (MAs) of the United States of America. We limit our focus to the four major ethnic groups: Non-Hispanic White, Black, Hispanic, and Asian.

We acknowledge that this approach simplifies the complexity of metropolitan populations. However, the stratification into ethnic groups is grounded in significant disparities in infection rates, which are linked to structural inequities and segregation patterns observed in urban areas. Ethnicity plays a key role in shaping both residential clustering and mobility patterns, which directly influence exposure to infection. Research has shown that ethnicity influences these factors more strongly than economic background^61^, making it a reliable indicator of segregated communities. Additionally, while access to quarantine and healthcare can vary within ethnic groups, community-level characteristics such as average wealth can provide a reasonable approximation of overall access to these resources. Given its strong alignment with variations in infection rates, residential clustering, and mobility, ethnicity serves as a meaningful proxy for understanding these dynamics. We recognize that this approach does not fully account for intra-group heterogeneity, such as variations in socioeconomic status or access to healthcare. However, this stratification reflects the granularity of the available data and avoids introducing assumptions that could lead to bias or overfitting.

In this setting, the population of each block $N_b$ will be set to its corresponding ethnic group’s population proportion, and the average wealth is then assigned to each group based on the empirical value. See Methods for details.

In this setting, the population of each block $N_b$ will be set to its corresponding ethnic group’s population proportion, and the average wealth of the ethnic group within each city is then assigned to that group based on the empirical value. See Methods for details.

We run the spreading model *independently* for each metropolitan area. Additionally, we consider a hypothetical scenario with perfect homogeneity which we call the *desegregated mixing*. In this scenario, each agent *i* is exposed to the other groups solely proportional to their population proportions and independent of its own block (SES/ethnic group) membership. (*See section* S7*in the Supplementary Information for the data and the detailed steps of this analysis.*)

**Fig. 3:**
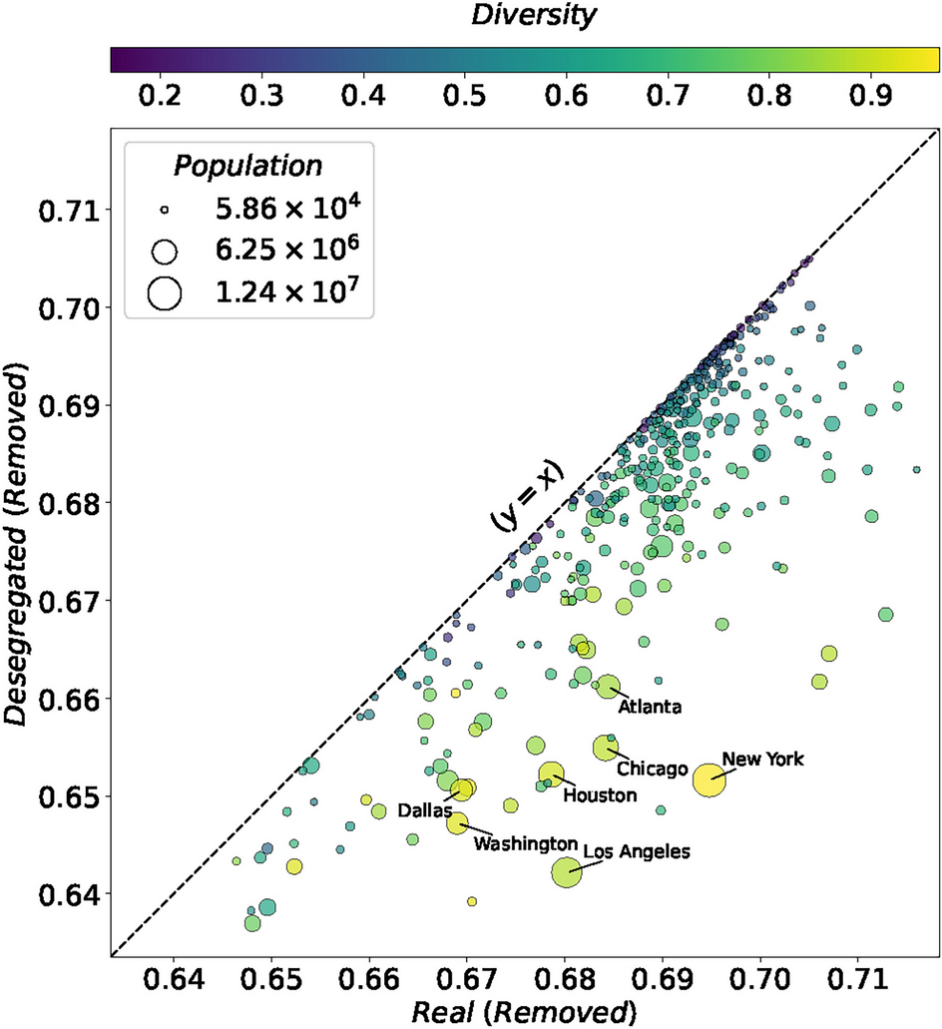
The outbreak size for the real and desegregated scenarios. Each point denotes one metropolitan area. The values on the x-axis and y-axis indicate the total removed population in the real and desegregated scenarios respectively. The size of each node represents the metropolitan area population. Colors denote the population diversity of each metropolitan area. The gray dashed line illustrates the identity ($y=x$) line.

In Fig. 3 we compare the outbreak size for the real (x-axis) and desegregated (y-axis) scenarios for each metropolitan area. Values below $y=x$ line show that real empirical segregation has a higher outbreak size compared to the hypothetical desegregated scenario with similar conditions. The colors illustrate the diversity of the population of each metropolitan area (normalized entropy of the ethnic composition^62^ (*See the Supplementary Information*). We observe that the outbreak size decreases for almost all metropolitan areas in the desegregated scenario. Additionally, more diverse metropolitan areas benefit further from desegregation.

### Case study of Covid-19 in Chicago: the effect of ethnic segregation on the infection trend

Using the modeling framework presented in this paper, we examine the case of early Covid-19 spread in the city of Chicago (not to be confused with the Chicago metropolitan area studied in the previous section). We again limit our analysis to the four major ethnic groups (Non-Hispanic White, Black, Hispanic, and Asian) and we use the daily data on Covid-19 cases by race/ethnicity in the city of Chicago^63^. We focus on the first months of the pandemic from the 14th of March 2020 to the 4th of July 2020 (113 days) to avoid exogenous effects such as vaccination and mandatory lockdowns.

We use the demographics of Chicago^64^ to find the proportional population of each ethnic group, average income, and segregation patterns. We use a maximum likelihood method to optimize and calibrate the other parameters of the model. (*See section* S8*in the Supplementary Information for the data and the detailed steps of this analysis*). Furthermore, we consider an alternative hypothetical scenario with perfect homogeneity in ethnic mixing, *desegregated mixing*, therefore removing the effect of segregation.

**Fig. 4:**
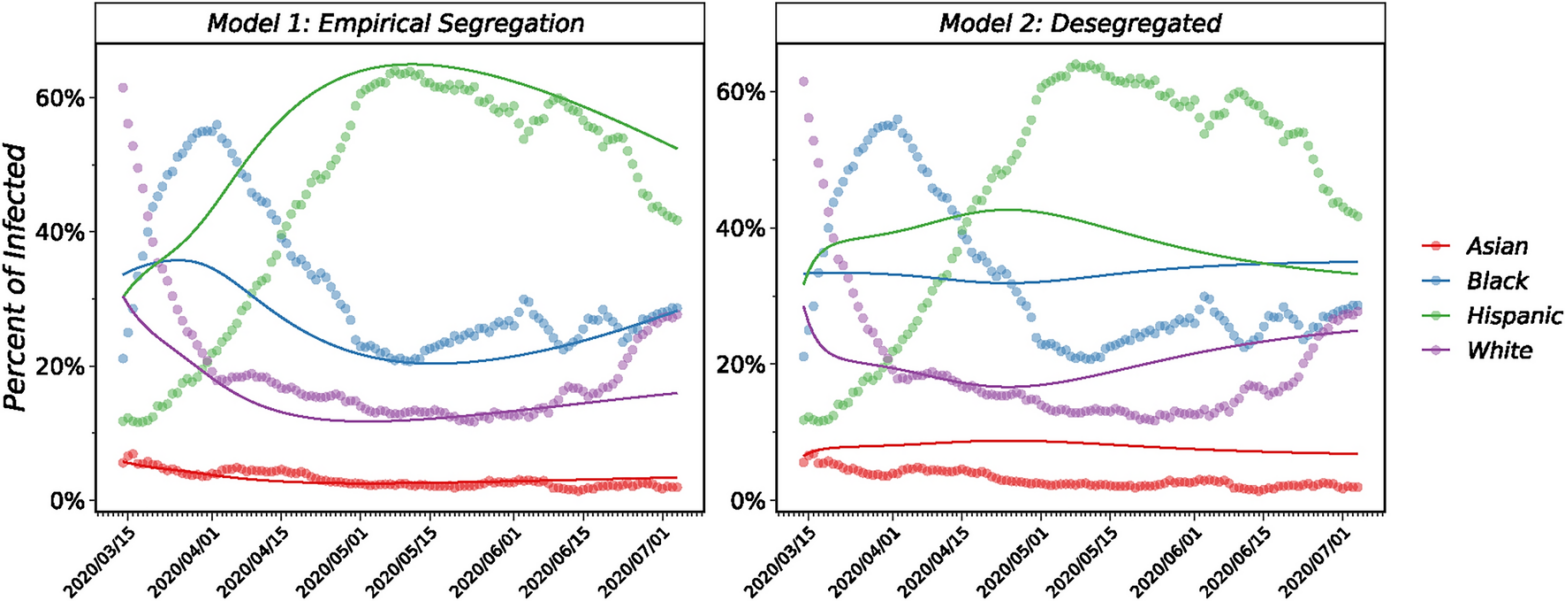
Percentage of the daily reported cases of Covid-19 coming from the four major ethnicities in Chicago. The color indicates ethnicity. The dots correspond to the empirical data, and the solid lines represent the model prediction. Values sum up to 100% at all time steps. The left panel represents our proposed model with empirical segregation, and the right panel represents the model without considering the segregation.

Figure 4 presents the results of our fitted models for the two scenarios, alongside empirical data. The panels illustrate the percentage of the daily reported cases of Covid-19 coming from the four major ethnicities in Chicago. The left panel shows the model incorporating empirical segregation, while the right panel assumes desegregated mixing. Our model with empirical segregation successfully captures the infection trends across ethnic groups over time, with a strong agreement emerging after the initial two weeks. This initial discrepancy may reflect early limitations in pandemic data collection, uneven testing access, and inconsistent documentation. It should be noted that, traditional spreading models, without considerations for the economic and/or structural inequalities, fail to capture these ethnic disparities and instead predict infection rates proportional to group populations.

## Discussion

With this modeling framework, we are able to evaluate the impact of two important dimensions of structural inequality, income inequality, and social segregation, on the health disparities in society. One key finding is that the level of income inequality in society matters in how the infection cases are distributed across social groups. When the income gap is high –because more low-income people will be obliged to go out to seek their livelihood– the infection rate will be higher than in a more equal society, where fewer people will have to go out to go to work despite the infection risk. Second, we find that higher segregation significantly intensifies the infection and subsequently mortality rates. It might seem that high segregation and low interaction among the groups, restrict the possibility of transmission across different SES groups, resulting in a lower infection rate. However, a high concentration of low SES individuals unable to quarantine can bring about a burst of infection in society. On the other hand, in a more uniform society, the low SES will be partially protected by the quarantine of their higher SES neighbors. Our computational model also predicts the appearance of a second peak in infection rates, due to the interplay between economic inequality and the decision-making procedure of the population. The low SES population gets infected until it reaches the point of herd immunity, the high SES on the other hand can afford to quarantine. However, after a drop in the low SES infectious cases, the high SES perceive the danger of infection to be low and move out of quarantine. This subsequently leads to a second infection peak. Furthermore, we inform our model parameters with the empirical data of the metropolitan areas of the United States. We also consider a hypothetical desegregated scenario for each area, where all groups are homogeneously mixed. We find that the infection rates are lower in the desegregated scenario, for most of the metropolitan areas.

Methodologically, this work is among the first papers to combine spreading dynamics on networks and game theory to model the dynamics of epidemic inequalities during a public health crisis. This computational framework allows us to compare different scenarios with respect to segregation and wealth inequality in a systematic manner. Moreover, we investigate the outcome of the simulations with the analytical approximation, enabling us to interpret the results in a reliable and robust way.

This modeling study is best evaluated at the onset of a new pandemic when vaccination solutions are not in place. In the future, it would be interesting to study how the delay in vaccinating lower-income classes can exacerbate health inequality and the time to reach immunity. In this paper, we assumed that each agent to have the same average number of links. In reality, lower-income groups often live in overpopulated housing estates in neighborhoods that suffer from a shortage of critical care physicians and medical supplies and work under conditions that do not allow the necessary distancing measures^65^. Hong el al.^66^ show this systematically by defining “Exposure density” as a metric for social distancing. They reveal significant disparities in social distancing practices across race, ethnicity, age groups, and socioeconomic factors in New York.

Nevertheless, the results of this model assumption can serve as the minimal condition of infection spread among the groups. Another direction for future research would be to include the dynamic of income inequality as a by-product of quarantining decisions. Finally, more detailed empirical data with information about the socioeconomic status of individuals, the willingness to participate in daily activities, and combined with spatial social networks would be beneficial to calibrate the computational model.

In summary, our study introduces a theoretical framework elucidating how population characteristics, such as socioeconomic class and social segregation, influence pandemic dynamics. Building on previous research, we demonstrated how income-associated self-quarantine ability shapes outbreaks and how existing inequalities and social segregation exacerbate disease spread. In a broader context, health inequalities can enforce other forms of inequalities and discrimination that should be tackled in a systematic and data-driven manner. These findings are crucial for policy-making, guiding the design of targeted social interventions.

## Methods

### Empirical analysis of metropolitan areas

We inform our computational framework by the empirical network and economic data of metropolitan areas (MA) of the United States of America. We limit our focus to the four major ethnic groups (Non-Hispanic White, Black, Hispanic, and Asian). We use the empirical values of exposure indices available in^67,68^ to construct the mixing patterns. The exposure index $\phi _{a,b}$ is the probability that a random member of *a* interacts with a member of *b*^62,69^. Exposure indices provide us with the probabilities of interaction in a stochastic block model. (*See the Supplementary Information for more detail*). Using the empirical population of each group and the interaction probabilities across the groups, we construct MA-specific SBMs. Therefore, SBM is a natural method for incorporating (ethnic) exposure indices in networks.

The income-loss punishment $r_b$ is adjusted based on the wealth distribution of each metropolitan area. We distribute the wealth in each metropolitan area under the assumption of the Pareto distribution. The Pareto distribution parameter, $\lambda$, is calculated based on the empirical value of the Gini coefficient in each metropolitan area available in^70^.

Hence, using the empirical data, we inform several of the parameters mentioned in Table S1. We keep $R_0 = 3$ as in the previous sections. As we have fewer SES groups compared to the synthetic models above, we will set $\beta = 5$ to better reflect the difference between the behavior of different blocks. The parameter $r_d$ is assumed to be $-100$ so that the outbreak size is in the range between 0.6 and 0.7. However, the robustness of the model with respect to these parameters is explored in the Supplementary Information (section S5).

## Supplementary Information

Supplementary Information.

## Data Availability

All the simulations, analyses and illustrations have been conducted via the unequal-spread package (written in Python), developed by Sina Sajjadi and Pourya Toranj Simin. This package is available under GPLv3, at https://github.com/Sepante/unequal-spread. A video summary of this study is available at: https://github.com/Sepante/unequal-spread/raw/master/video-abstract/abstract-vid.mp4.

## Footnotes

1 In this paper, quarantine refers specifically to self-quarantine: voluntary confinement at home. This act is distinguished from isolation, which applies to confirmed infectious cases, and social distancing, which allows external activities^25^.
