# Supplementary material for: Structural inequalities exacerbate infection disparities
Source: Sci Rep. 2025 Mar 17;15:9082. doi: 10.1038/s41598-025-91008-w (PMC11914215; doi:10.1038/s41598-025-91008-w)
Supplement: Supplementary file 1 — Supplementary Information. [file 41598_2025_91008_MOESM1_ESM.pdf]

Supplementary Information

S1 Agent-based simulation details

In this section, we provide the details of the agent-based simulation, necessary for the reproduction of our results. In this model each agent can be in one of the three different states: "SIR" (Susceptible-Infected-Removed). Agents are also classified into SES blocks, affecting their decision on whether or not to quarantine at each time step.

The Initial state, dynamics, and final state of the simulation are described in the following sections.

S1.1 Initial State

A proportion of size I_{init} within each SES block is set to state I. The rest of the population is in state S.

S1.2 Dynamics

The infection simulations are conducted using the rejection-based modeling[71]. In this method, all possible actions (infection, removal, or quarantine) will be proposed at each time step and they will be either accepted or rejected, based on their corresponding probability. The simulation steps consist of the following:

1. *Quarantine phase:* Every agent decides to quarantine, with the probability $1-P_b(I)$, based on its SES block membership, b and the total infectious fraction of the population, $I(t)$ at the time.

2. *Infection phase:* Every non-quarantining infectious agent, turns its non-quarantining susceptible neighbors to infectious with the infection probability $\mu\Delta t$, where μ is the transmission rate and Δt is the size of a time step.

3. *Removal phase:* Every infectious agent gets removed with the removal probability $\gamma\Delta t$, where γ is the rate of removal[60].

As we employ a parallel updating algorithm, the transitions in agents' health status only take effect after the end of each time step. Therefore, an agent turning infectious at time step t, can only transmit the infection and/or get removed, in time steps t', where $t' > t$.

S1.3 Final State

The simulation runs until it reaches the stationary state, i.e., until all of the agents are in one of the states, S or R. This holds true for the SIR model and extensions such as the SEIR model, however, for extensions such as SIRS, we consider the more general definition of the stable state ($\frac{dS}{dt} = \frac{dI}{dt} = \frac{dR}{dt} = 0$). (see S6).

S2 Mean-field approximation

In this section, we derive the mean-field approximation for our agent-based model. For this purpose, we first define the concept of "compartment" as the set of agents with the same SES and health (S-I-R) status. Secondly, we take into account the following assumptions:

1. Homogeneous Mixing: Agents within each compartment are identical and equally exposed to the rest of the population.

2. Determinism: The stochastic fluctuations of the system can be neglected as they are considered inconsequential on large scales.

3. Continuity: The progress of the dynamics can be approximated by a continuous time dimension instead of discrete time steps.

As a result of the first assumption, the state of the system can be represented by the size of the compartments (fractions of S, I, and R agents within each SES group). By incorporating the second and third assumptions, the dynamics can be described by a set of exchange rates between the compartments in the form of differential equations.

The transitions in the model consist of the following:

$$S + I \rightarrow I + I \tag{S1}$$

$$I \rightarrow R \tag{S2}$$

To obtain the transition rates between the compartments, we formulate Eq. S3, where $\widetilde{\Omega_i}$ is the probability of the transmission (Eq. S1) <u>not</u> to occur for an agent i of block b, during a time period of Δt. The first term on the right hand side denotes the case in which agent i is under quarantine, and the second term represents the situation where agent i is not in quarantine (P_b) but does not get infected (Q_i).

$$\widetilde{\Omega_i} = (1-P_b) + P_b Q_i \tag{S3}$$

To derive Q_i, we focus on the probability of transmission from a specific agent j in block b'. For this transmission to occur, agent j should be both infectious and out of quarantine. Given that this condition holds for a period of Δt, the probability of transmission would be $\mu\Delta t$, as μ is the infection probability in one time-step in the discrete model. Therefore, $\Omega_{j\rightarrow i}$ the overall probability of a susceptible agent i which is out of quarantine, getting infected by agent j from block b' is as formulated in Eq. S4.

$$\Omega_{j\rightarrow i} = P_{b'} \frac{I_{b'}}{N_{b'}} (\mu\Delta t) \tag{S4}$$

Where $I_{b'}$ is the number of infected agents in block b' and $N_{b'}$ is the population of block b'; therefore due to the homogeneous mixing assumption, $\frac{I_{b'}}{N_{b'}}$ is the probability that agent j from block b' is infected.

The probability Q_i that the not-quarantined agent i from block b, is not getting infected by any of its neighbors j is obtained in Eq. S5.

$$\begin{aligned} Q_i &= \prod_{j=1}^{k_i}(1-\Omega_{j\to i}) = \prod_{j=1}^{k_i}(1-P_{b'}\frac{I_{b'}}{N_{b'}}\mu\Delta t) \\ &= \prod_{b'=1}^{B}\prod_{j}^{\{\text{Neighbors of i in } b'\}}(1-P_{b'}\frac{I_{b'}}{N_{b'}}\mu\Delta t) \\ &= \prod_{b'=1}^{B}(1-P_{b'}\frac{I_{b'}}{N_{b'}}\mu\Delta t)^{k_{i,b'}} \end{aligned} \tag{S5}$$

Where $k_i \approx \langle k\rangle$ is the total number of i's neighbors and $k_{i,b'}$ is the number of i's neighbors within block b'. As $\phi_{b,b'}$ is the share of neighbors of $i \in b$ which are from block b', then $k_{i,b'} \approx \phi_{b,b'}k_i$. (See section S3.1) We can, therefore, approximate Eq. S5 as in Eq. S6. We note that this approximation is suitable only for networks with narrow degree distributions. The mean-field approximation for networks with broad degrees requires further consideration and might lead to qualitatively different results[72]. (Also see S3).

$$\begin{aligned} Q_i &= \prod_{b'=1}^{B}(1-P_{b'}\frac{I_{b'}}{N_{b"}}\mu\Delta t)^{k_{i,b'}} \\ &\approx \prod_{b'=1}^{B}(1-P_{b'}\frac{I_{b'}}{N_{b'}}\mu\Delta t)^{\phi_{b,b'}\langle k\rangle} \\ &\approx \prod_{b'=1}^{B}(1-\phi_{b,b'}\langle k\rangle P_{b'}\frac{I_{b'}}{N_{b'}}\mu\Delta t) \\ &\approx \prod_{b'=1}^{B}(1-\rho_{b,b'}P_{b'}I_{b'}\mu\Delta t) = \prod_{b'=1}^{B}(1-\alpha_{b'}\Delta t) \end{aligned} \tag{S6}$$

Where we have defined $\alpha_{b'} \equiv \rho_{b,b'}P_{b'}I_{b'}\mu$, and by rephrasing Eq. S6 we have Eq. S7.

$$\begin{aligned} &\prod_{b'=1}^{B}(1-\alpha_{b'}\Delta t) = \exp\left[\ln\left(\prod_{b'=1}^{B}(1-\alpha_{b'}\Delta t)\right)\right] \\ &= e^{\Sigma_{b'=1}^{B}\ln(1-\alpha_{b'}\Delta t)} \end{aligned} \tag{S7}$$

As $\Delta t \ll 1$ we can approximate $\ln(1-\alpha_{b'}\Delta t) \approx -\alpha_{b'}\Delta t$ obtaining Eq. S8.

$$\begin{aligned} Q_i &\approx \prod_{b'=1}^{B}(1-\alpha_{b'}\Delta t) \approx e^{-\Sigma_{b'=1}^{B}\alpha_{b'}\Delta t} \\ &= 1-\sum_{b'=1}^{B}\alpha_{b'}\Delta t + O(\Delta t^2)+\ldots \\ &\approx 1-\sum_{b'=1}^{B}\alpha_{b'}\Delta t = 1-\sum_{b'=1}^{B}\rho_{b,b'}P_{b'}I_{b'}\mu\Delta t \end{aligned} \tag{S8}$$

Having Q_i, we derive Ω_i, the probability that agent i from block b gets infected by any of its neighbors in Eq. S9.

$$\Omega_i = 1-(1-P_b+P_bQ_i) \tag{S9}$$

By substituting Q_i in Eq. S9 we obtain Eq. S10.

$$\begin{aligned} \Omega_i &\approx 1-\left(1-P_b+P_b(1-\sum_{b'=1}^{B}\rho_{b,b'}P_{b'}I_{b'}\mu\Delta t)\right) \\ &= \mu P_b\sum_{b'=1}^{B}P_{b'}I_{b'}\rho_{b,b'}\Delta t \end{aligned} \tag{S10}$$

The expected value of the number of infected agents after a time duration of size Δt and hence the change in the number of susceptible agents S_b in block b can be derived as in Eq. S11.

$$\begin{aligned} \Delta S_b &= -\sum_{i\in S_b}\Omega_i \approx -\sum_{i\in S_b}\mu P_b\sum_{b'=1}^{B}P_{b'}I_{b'}\rho_{b,b'}\Delta t \\ &= -\mu\left(\sum_{i\in S_b}\right)\left(P_b\sum_{b'=1}^{B}P_{b'}I_{b'}\rho_{b,b'}\Delta t\right) \\ &= -\mu S_bP_b\sum_{b'=1}^{B}P_{b'}I_{b'}\rho_{b,b'}\Delta t \end{aligned} \tag{S11}$$

By differentiating S_b with respect to time we arrive at Eq. S12.

$$\frac{dS_b(t)}{dt} = -\mu S_b(t)P_b\sum_{b'=1}^{B}P_{b'}I_{b'}\rho_{b,b'} \tag{S12}$$

On the other hand due to the transition in Eq. S2, the infected agents will get removed with a probability of $\gamma\Delta t$. Therefore, ΔR_b, the number of infectious agents in block b that will get removed after a time duration of size Δt can be expressed in Eq. S13.

$$\Delta R_b = I_b\gamma\Delta t \tag{S13}$$

Resulting in Eq. S14.

$$\frac{dR_b(t)}{dt} = \gamma I_b(t) \tag{S14}$$

As a result of the conservation of the population of each block, we also have Eq. S15.

$$S_b(t)+I_b(t)+R_b(t) = N_b \tag{S15}$$

By putting the equations S12, S14, and S15 together, we arrive at the set of ordinary differential equations describing the dynamics of the system in Eq. S16.

$$\begin{aligned} &\frac{dS_b(t)}{dt} = -\mu S_b(t)P_b\sum_{b'=1}^{B}P_{b'}I_{b'}\rho_{b,b'} \\ &\frac{dR_b(t)}{dt} = \gamma I_b(t) \\ &S_b(t)+I_b(t)+R_b(t) = N_b \end{aligned} \tag{S16}$$

In the main text, Fig. 2 panel a illustrates the mean-field value of the infectious compartment size over time, ($I_b(t)$ in

Eq. S16) as solid lines. Fig. 2 panel c illustrates the final ($t \to \infty$) outbreak size ($\lim_{t\to\infty} R(t)$ in Eq. S16) as solid lines. Fig. 2 panel e, Fig. S3 and Fig. S5 illustrate the same parameter as the color code.

By dividing the first two equations in Eq. S16 by γ, we will obtain Eq. S17.

$$\begin{aligned}\frac{dS_b(t)}{\gamma dt} &= -\frac{\mu}{\gamma} S_b(t) P_b \sum_{b'=1}^{B} P_{b'} I_{b'} \rho_{b,b'} \\ \frac{dR_b(t)}{\gamma dt} &= I_b(t) \\ S_b(t) + I_b(t) + R_b(t) &= N_b\end{aligned} \tag{S17}$$

We can scale the time dimension $dt \to dt' = \gamma dt$. By doing so, the dynamical equations would evolve at a different time scale, however, the stationary state (final outbreak size) would be invariant. In addition, considering $\phi_{a,b} = \frac{\rho_{a,b} N_b}{\langle k \rangle}$ (see section S3.1) and replacing ρ we will have Eq. S18.

$$\begin{aligned}\frac{dS_b(t)}{dt'} &= -\frac{\mu \langle k \rangle}{\gamma} \frac{1}{N_b} S_b(t) P_b \sum_{b'=1}^{B} P_{b'} I_{b'} \phi_{b,b'} \\ \frac{dR_b(t)}{dt'} &= I_b(t) \\ S_b(t) + I_b(t) + R_b(t) &= N_b\end{aligned} \tag{S18}$$

As the parameters μ, γ and $\langle k \rangle$ only appear in the form $\frac{\mu \langle k \rangle}{\gamma}$, we can introduce $R_0 = \frac{\mu \langle k \rangle}{\gamma}$ so that they are all incorporated in the parameter R_0.

S3 Segregation

To model segregated networks, we use stochastic block models (SBMs). SBMs are capable of demonstrating a controllable level of modularity[73] i.e., higher intra-group and lower inter-group connections, making them a good candidate for modeling community structures. In the stochastic block model, every two agents, i and j respectively belonging to blocks a and b, are connected with probability $\rho_{a,b}$, where $\boldsymbol{\rho}$ is known as the probability matrix. Here we consider the "blocks" to be identical to socioeconomic groups.

First, we construct a fully segregated $\boldsymbol{\rho}^{(\mathrm{hg})}$ (hg for heterogeneous) and a fully homogeneous matrix $\boldsymbol{\rho}^{(\mathrm{hm})}$ ((hm for homogeneous)) . In the former case, only links within blocks are allowed. Therefore, to maintain a uniform mean degree $\langle k \rangle$ across the blocks, we set $\rho^{(\mathrm{hg})}_{a,b} = \delta_{a,b} \frac{\langle k \rangle}{N_a}$ with N_a indicating the number of nodes within block a. In the latter case, agents do not discriminate based on block memberships and therefore $\rho^{(\mathrm{hm})}_{a,b} = \frac{\langle k \rangle}{N_T}$ with N_T indicating the total number of the nodes.

It should be noted that the current method is suitable for mesoscale social networks with narrow degree distributions. However, the generalization would be straightforward using the degree-corrected SBM[39] with an imposed degree distribution.

To construct a probability matrix with an arbitrary level of segregation we define $\boldsymbol{\rho}^{(s)} = s \times \boldsymbol{\rho}^{(\mathrm{hg})} + (1-s) \times \boldsymbol{\rho}^{(\mathrm{hm})}$ as a linear combination of the aforementioned matrices, where s indicates the intensity of segregation in the matrix.

Using $\boldsymbol{\rho}^{(s)}$ we generate networks with desired segregation levels, as illustrated in Fig. S1.

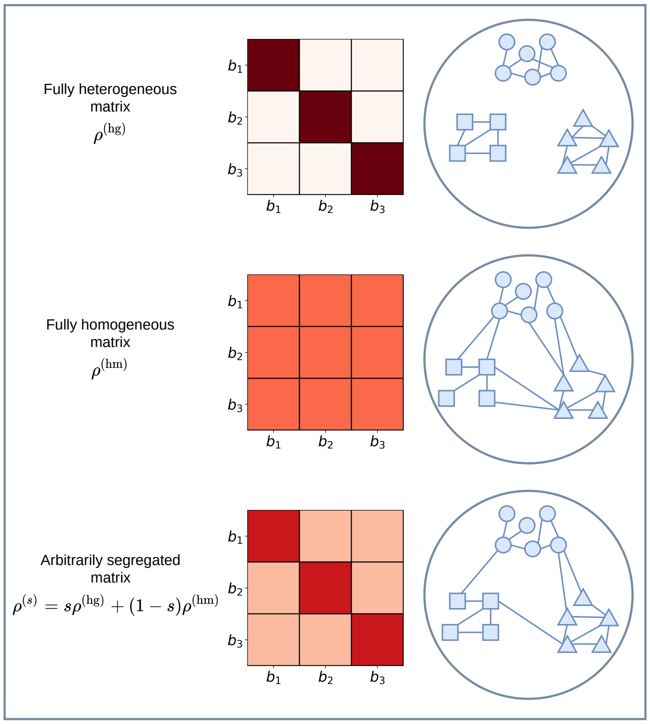

Figure S1. Segregation in the contact network: The value of segregation controls the interaction between individuals of different SES groups. By changing the value of s, we move from a fully homogeneous society to a fully segregated regime, so the on-diameter and off-diameter values of the probability matrix respectively increase and decrease. This variation is also reflected in the toy network illustrations. In the toy illustrations, shapes denote socioeconomic blocks. By increasing the value of s the number of intra-block and inter-block links respectively increase and decrease.

S3.1 Share of Connections

To calculate $\phi_{a,b}$, the share of neighbors from block b for agent $i \in a$, we divide the average number of i's neighbors in block b by the average number of i's total neighbors. Therefore, we obtain $\phi_{a,b} = \frac{\rho_{a,b} N_b}{\langle k \rangle}$. It should be noted that in contrast to $\boldsymbol{\rho}$, $\boldsymbol{\phi}$ is not symmetric and is invariant under the population size N_T.

$\phi_{a,b}$ is also known as the exposure index and has been defined as the probability that a random member of a interacts with a member of b[62,69].

Parameter	Description	Value
Contact Network		
B	Number of SES groups	10
N_T	Total number of agents	10^4
N_b	Population of block b	$N_b = \frac{N_T}{B}$
$\langle k \rangle$	Mean degree	20
s	Segregation	0.5
Spreading Dynamics		
R_0	Reproductive number	$\frac{\mu \langle k \rangle}{\gamma} = 3$
I_{init}	Initial infectious fraction	0.001
γ	Removal rate	0.03
μ	Transmission rate	0.0045
Decision Making		
r_d	Fear of infection	-10
β	Intensity of selection	3
λ	Indicator of equality	1.5

Table S1. **Model parameters and their baseline values.**

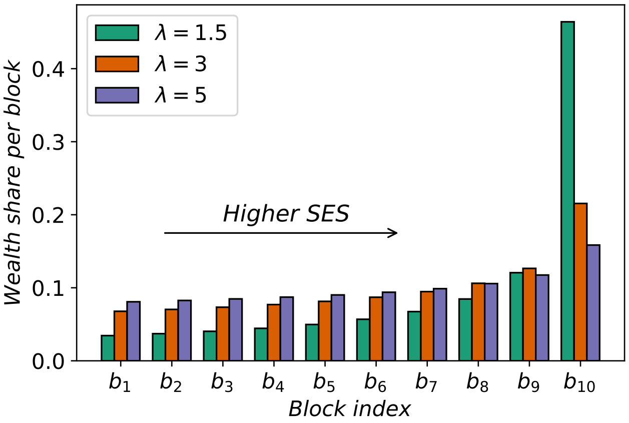

Figure S2. Distribution of the wealth among 10 blocks, via the Pareto distribution with exponents $\lambda = 1.5, 3, 5$. The arrow indicates moving from the lower to higher SES groups.

S4 Economic Inequality

The process of decision-making is governed by an economic game, where agents decide whether to quarantine themselves or to participate in society. Going to work exposes the agent to the infection, resulting in a *psychological fear of infection* punishment. The option of quarantining, in contrast, leads to *income loss*. The effect of income loss is higher for individuals from lower socioeconomic classes, as they will be less likely to afford to lose their income and quarantine themselves. Hence, we hypothesize the punishment of income loss for the agents to be inversely proportional to their share of wealth. As also mentioned in the main manuscript, we take note that this dynamics only represents the self-imposed quarantine. Top-down confinement measures are out of the scope of this paper but are interesting to incorporate in future works.

We assume wealth w is distributed across the discrete socioeconomic classes, by the Pareto distribution $p(w) = \frac{\lambda w_m^\lambda}{w^{\lambda+1}}$, where members of each class have identical wealth. The parameter λ controls the level of equality, where $\lambda \to 0$ and $\lambda \to \infty$ respectively represent the ultimate inequality (Dirac delta distribution of wealth) and equality (Uniform distribution of wealth). Wealth in turn controls the fear of income loss ($r_b = -\frac{1}{w_b}$). Knowing the wealth distribution, we can calculate the share of wealth for different SES groups. Let F be the cumulative portion of the population from poorest to richest, and L be the cumulative share of wealth; then, for Pareto distribution, we have:

$$L(F) = 1 - (1-F)^{\frac{\lambda-1}{\lambda}} \tag{S19}$$

This equation indicates the proportion of wealth owned by the F fraction of the population. In Fig. S2 we illustrate the share of wealth for each block of the population, for different values of λ.

We further assume the fear of income-loss r_b to be proportional to the inverse of each SES group's wealth:

$$r_b = -\frac{1}{w_b} \tag{S20}$$

We thus, hypothesize the probability P_b of participation (not quarantining) for agents of block b to be:

$$P_b(t) = \frac{1}{e^{\beta(r_b - r_d I(t))} + 1} \tag{S21}$$

S5 Robustness with respect to model parameters

In this section, we vary the baseline parameter values to further check the robustness of our analysis.

S5.1 Transmission Parameters

We focus on Eq. S18. As mentioned in S2, μ, γ and $\langle k \rangle$ are all incorporated in the parameter R_0. Hence, by varying R_0 we inspect the robustness of the results with respect to all three aforementioned parameters.

The results of this analysis are illustrated in Fig. S3. Unsurprisingly, increasing R_0 (moving upward) increases the outbreak size. More importantly, increasing λ (moving horizontally to the right) lowers the prevalence of the infection, for all R_0 values. Further supporting our results with respect to the λ equality index in section 2.

S5.2 Policy and behavioral variations

In this section, we discuss the versatility of our model to further incorporate the effect of pandemic-related policies and other city-specific characteristics which can affect the outbreak of the infection. Top-down lockdowns have been one of the widespread non-pharmaceutical interventions (NPI) to

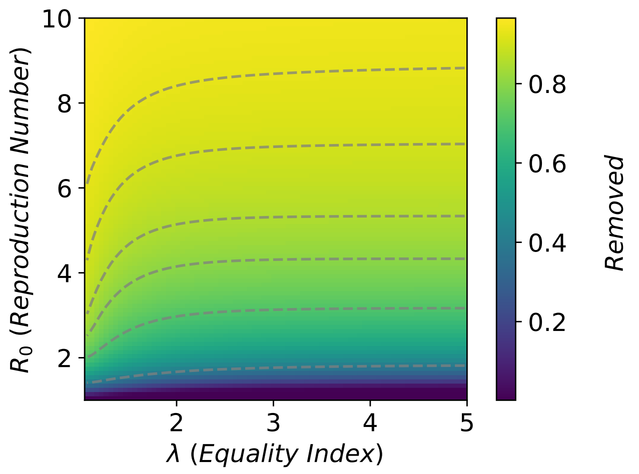

Figure S3. Outbreak size, R in the society for varying levels of transmission rate and equality. The x-axis is the parameter λ, the indicator of wealth equality. The y-axis is the reproduction number of infection R_0, controlling the transmission rate of the disease. Color-axis represents the proportion of the removed agents R at the end of the dynamics. The results are based on the mean-field approximation. Dashed lines are guides for the eye.

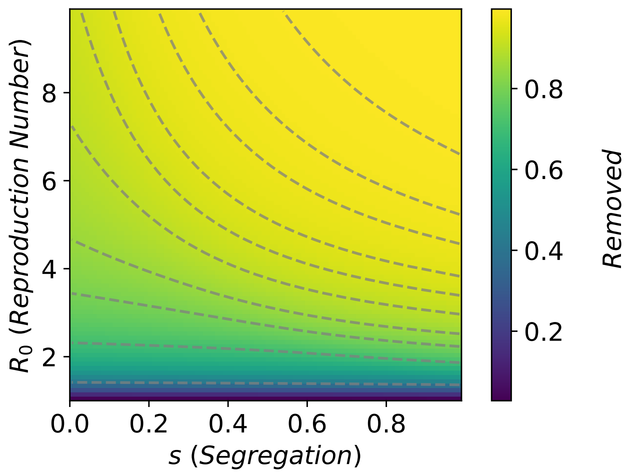

Figure S4. Outbreak size, R in the society for varying levels of transmission rate and segregation. The x-axis is s, the indicator of segregation. The y-axis is the reproduction number of infection R_0, controlling the transmission rate of the disease. Color-axis represents the proportion of the removed agents R at the end of the dynamics. The results are based on the mean-field approximation. Dashed lines are guides for the eye.

encounter pandemics such as Covid-19. While top-down confinement measures are out of the scope of this paper, we note that our framework allows for such interventions. For example, the quarantine we included in the model can be triggered due to extrinsic probabilistic methods. Various models can be devised to incorporate such dynamics in future works.

Another common NPI is the mask mandate. By lowering the droplet dispersion chance, masks reduce the infection probability[74]. Our modeling framework can reflect the incorporation of the mask by varying the infection probability and R_0 as we will perform in the following.

Additionally, we focus on the city-specific characteristics affecting the dynamics. One such feature would be the variation in behavioral responses. Our model can reflect these diversities in different ways. Some examples would be variations in sensitivity towards the infection prevalence, the intensity of the fear of infection due to awareness and media, and also the overall economic inequality. The parameters, controlling these characteristics would be respectively β, r_d, and λ. Another city-specific property would be the level of segregation, controlled by s. While in the following sections, we will focus on the empirically obtained segregation patterns, in this section we focus on the variations of this parameter in the baseline abstract model.

In Figs. S5, S7, S8, and S9 we have illustrated the results of these analyses varying two parameters at a time and analyzing the outbreak size.

In Fig. S5, by intensifying the fear of the infection, r_d, (lower negative values), the population will act more vigilantly and therefore the outbreak size decreases. More importantly, increasing λ (moving horizontally to the right) lowers the prevalence of the infection, for all r_d values, further supporting our results with respect to the λ equality index in section 2. To better understand the effect of λ on the decision-making probability and hence the spreading dynamics, in Fig. S6 we illustrate the Probability of participation, P_b (Eq. 1) for each block b as a function of the proportion of infectious population I. Each diagram corresponds to a unique value of λ. Higher values of λ and more equal wealth distributions lead to more similar probabilities across the blocks.

In Fig. S7, we observe similar results for the fear of infection r_d. For β we find that the increase in the selection intensity, increases the infection prevalence, hinting that short-sighted benefit maximization will lead to a larger outbreak size.

In Fig. S8, we find that an increase in R_0 results in an increase in the outbreak size, similar to the findings in Fig. S3. Our results for β also parallel the previous results, as higher β values intensify the infection prevalence.

In Fig. S9, we again find that the increase in β increases the outbreak size. For λ values, however, we find results partially contradicting our previous findings, as for some high β values, the increase of the equality index does not necessarily lead to a drop in infection rates. To better interpret these results we illustrate the behavior of the decision-making function as a function of β in Fig. S10. As we can observe, very low values of β lead to the complete insensitivity of the agents to the infection prevalence in the system, as they would fully

randomly (with a probability of 0.5) decide whether to participate or not. On the other hand, very high values of β would result in a deterministic step function, removing the randomness from the participation decision. In this case, with high inequality ($\lambda \to 0$), higher SES blocks, having a very high sensitivity to the infection, would fully seclude themselves from the system, leading to a drop in the infection prevalence, as only the lower SES groups will remain in circulation. As both extremes would be unrealistic, we will limit β to values between 2 and 5 so that the model would exhibit randomness, sensitivity to the infection rate, and also class disparities.

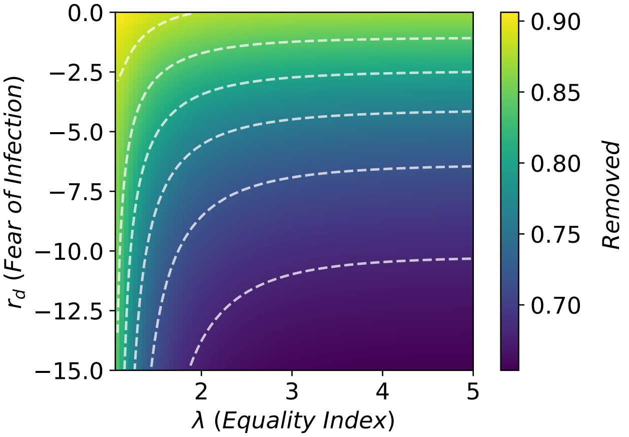

Figure S5. Outbreak size, R in the society for varying levels of fear of infection and equality. The x-axis is the parameter λ, the indicator of wealth equality. The y-axis is the parameter r_d, the indicator of the fear of infection. Color-axis represents the proportion of the removed agents R at the end of the dynamics. The results are based on the mean-field approximation. Dashed lines are guides for the eye.

S5.3 Diversified Initial Conditions

For the dynamics analyzed in the main manuscript, initially a fraction I_{init} of agents in each block are in the state I, with the rest being in the state S. In this section, we investigate the robustness of the dynamics with regard to this assumption. To this end, we consider two alternative distributions of the initial infectious agents. In one, all the initial infectious agents are from the highest SES group (High SES Outbreak), and in the other, they all belong to the lowest SES group (Low SES Outbreak). In both scenarios, we start with the same proportion of initially infectious agents as the baseline assumption.

In Fig. S11 we illustrate the time series of the infectious population for the three scenarios. Although we still run the model on 10 SES groups, for more clarity, we only visualize the highest (green) and the lowest SES groups.

We observe that qualitatively the dynamics follow the same patterns and the method is robust with respect to this variation. As we would intuitively expect, the peak infection of the lowest SES group would be respectively achieved earlier and later in the Low SES outbreak and the High SES outbreak scenarios. This could be interesting for future studies.

S6 Robustness with respect to model variations

In this section, we consider more generalized models and test the robustness of our results under these variations.

S6.1 Latent period

For many diseases such as smallpox or SARS, the infected individuals do not become infectious right at the time of infection but become one after a time interval known as the latent period[75]. This period is accounted for in the SEIR (Susceptible, Exposed, Infectious, Recovered) compartmental model[59]. To study the robustness of our model under more generalized scenarios, we further extend our adaptive model to also account for this dynamics. To do so, we keep intact the contact network and the decision-making process but modify our spreading dynamics from SIR to the SEIR model. Therefore, our Eq. S16 will be rewritten as in Eq. S22.

$$
\begin{aligned}
\frac{dS_b(t)}{dt} &= -\mu S_b(t) P_b \sum_{b'=1}^{B} P_{b'} I_{b'} \rho_{b,b'} \\
\frac{dE_b(t)}{dt} &= +\mu S_b(t) P_b \sum_{b'=1}^{B} P_{b'} I_{b'} \rho_{b,b'} - \sigma E_b(t) \\
\frac{dR_b(t)}{dt} &= \gamma I_b(t) \\
S_b(t) &+ I_b(t) + E_b(t) + R_b(t) = N_b
\end{aligned}
\tag{S22}
$$

As indicated in the Eq. S22 the infected agents do not directly join the I (Infectious) compartment, but first, go through the transitionary E (Exposed) compartment. By adding E, we also have to consider another parameter σ that denotes the rate by which the exposed individuals become infectious. In the real world, this rate σ is mostly higher than the recovery rate γ[75]. On the other hand, very high values of σ will lead to dynamics similar to SIR, as the exposed individuals will be almost instantly moved to the infectious compartment. Considering these two limits we alter the value of σ to check the robustness of our results in the main manuscript.

In Fig. S12 we illustrate the time series of the infectious population for the same four representative compartments as the main manuscript, this time using the SEIR dynamics (Eq. S22). We alter the value of σ across the panels while keeping the other parameters as the baseline S1. We observe that the main infection disparities are still pronounced in this extended model. We also observe the second peak of infection for higher SES compartments. Although the second peak becomes less significant, it is still visible in dynamics even with low values of σ. We also observe the convergence of the dynamics to the baseline SIR, with relatively high σ values. To further inspect the two main results, we study the effect of economic inequality and segregation on the overall outbreak

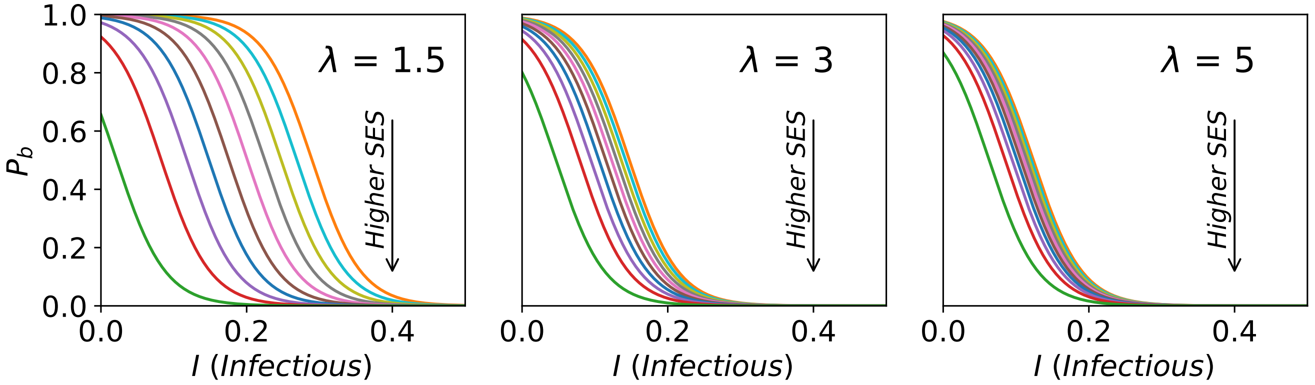

Figure S6. Probability of participation, P_b (Eq. 1) for each block b as a function of the proportion of infectious population I. The arrows indicate moving toward the higher SES groups. Each diagram corresponds to a unique value of λ. More equal wealth distribution leads to less deviating probabilities.

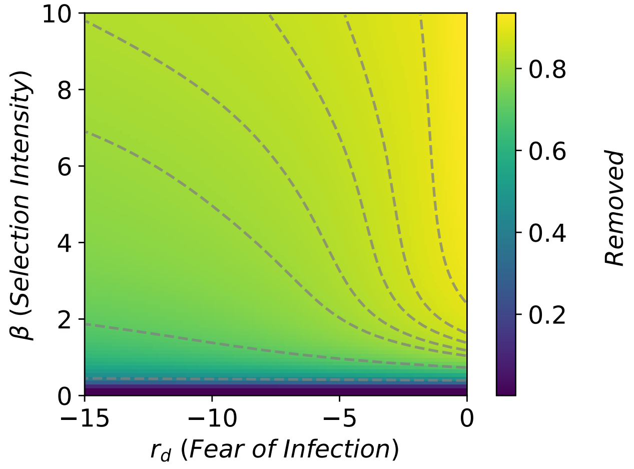

Figure S7. Outbreak size, R in the society for varying levels of fear of infection and selection intensity. The x-axis is the parameter r_d, the indicator of the fear of infection. The y-axis is β, the indicator of selection intensity. Color-axis represents the proportion of the removed agents R at the end of the dynamics. The results are based on the mean-field approximation. Dashed lines are guides for the eye.

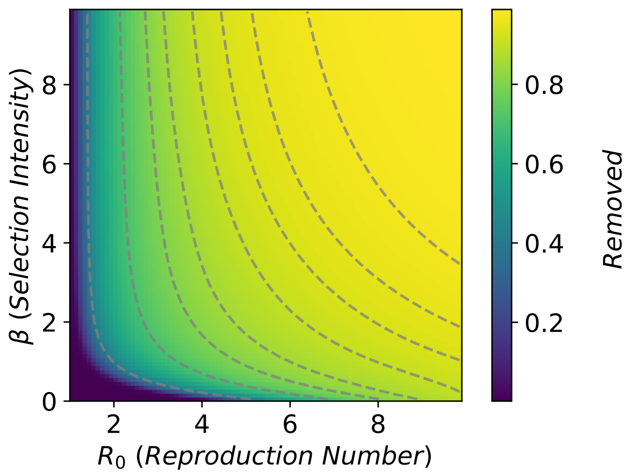

Figure S8. Outbreak size, R in the society for varying levels of transmission rate and selection intensity. The x-axis is the reproduction number of infection R_0, controlling the transmission rate of the disease. The y-axis is β, the indicator of selection intensity. Color-axis represents the proportion of the removed agents R at the end of the dynamics. The results are based on the mean-field approximation. Dashed lines are guides for the eye.

size. In Fig. S13 we vary the values of the economic equality index λ and the exposed-to-infectious rate σ and in Fig. S14 we vary the values of the segregation index s and the exposed-to-infectious rate σ. As we observe both higher segregation (high s) and higher economic inequality (low λ) exacerbate the outbreak sizes, additionally confirming the robustness of our results.

S6.2 Waning immunity

The immunity that occurs after infection is not always permanent. Re-infection is possible for many diseases[75] including Covid-19 and Influenza A[76]. This dynamics is accounted for in the SIRS (Susceptible, Infectious, Recovered, Susceptible) compartmental model[59]. To study the robustness of our model under more generalized scenarios, we further extend our adaptive model to also account for this dynamics. To do so, we keep intact the contact network and the decision-making process but modify our spreading dynamics from SIR to the SIRS model. Therefore, our Eq. S16 will be rewritten as in Eq. S23.

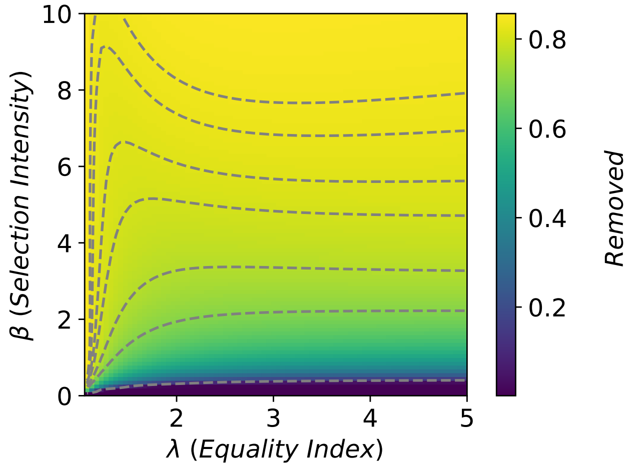

Figure S9. Outbreak size, R in the society for varying levels of equality and selection intensity. The x-axis is the parameter λ, the indicator of wealth equality. The y-axis is β, the indicator of selection intensity. Color-axis represents the proportion of the removed agents R at the end of the dynamics. The results are based on the mean-field approximation. Dashed lines are guides for the eye.

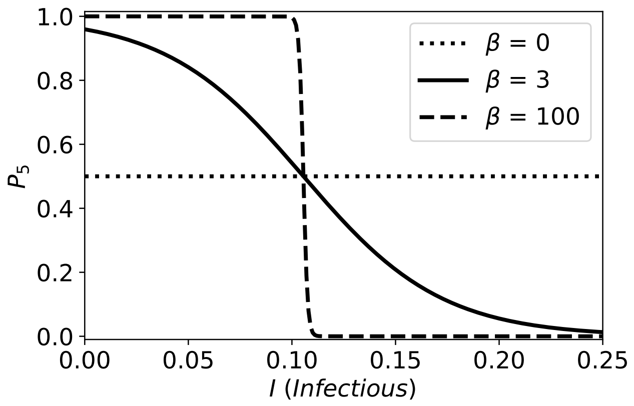

Figure S10. Effect of the selection intensity, β, on the decision making. Probability of participation, P_5 (Eq. 1) for block $b = 5$ is illustrated as a function of the proportion of infectious population I. Each diagram corresponds to a unique value of β. Very large β values lead to insensitivity to the infectious population and very high β values converge to a step function.

$$\frac{dS_b(t)}{dt} = -\mu S_b(t) P_b \sum_{b'=1}^{B} P_{b'} I_{b'} \rho_{b,b'} + wR_b(t)$$
$$\frac{dR_b(t)}{dt} = \gamma I_b(t) - wR_b(t) \tag{S23}$$
$$S_b(t) + I_b(t) + R_b(t) = N_b$$

As indicated in Eq. S23 the recovered agents do not remain recovered indefinitely but move back to the Susceptible (S) compartment with rate w. w denotes the immunity loss rate. In the real world, the rate w is mostly lower than the recovery rate γ[75]. On the other hand, very low values of w will lead to dynamics similar to SIR, as the recovered individuals will remain in the recovered state, almost indefinitely. Considering these two limits we alter the value of w to check the robustness of our results in the main manuscript.

In Fig. S12 we illustrate the time series of the infectious population for the same four representative compartments as the main manuscript, this time using the SIRS dynamics (Eq. S22). We alter the value of w across the panels while keeping the other parameters as the baseline S1. We observe that the main infection disparities are still pronounced in this extended model. We also observe the second peak of infection for higher SES compartments. We can further observe multiple peaks of the infectious population for very low but non-zero w values, which would be interesting for future studies. We also observe the convergence of the dynamics to the baseline, SIR, with $w = 0$.

To further inspect the two main results we study the effect of economic inequality and segregation on the overall spread of the infection. However, contrary to the stable states of SIR and SEIR models, the stable states of the SIRS model do not only consist of agents in the S and R states. The SIRS model makes possible the formation of endemic states of the diseases, in which a stable infectious population persists in the system[59]. Therefore, to study this set of equations, we consider the more general definition of the stable state ($\frac{dS}{dt} = \frac{dI}{dt} = \frac{dR}{dt} = 0$). Therefore, to analyze the prevalence of the infection, we focus on the stable infectious population in the endemic state. In Fig. S16 we vary the values of the economic equality index λ and the exposed-to-infectious rate σ and in Fig. S17 we vary the values of the segregation index s and the exposed-to-infectious rate w. As we observe both higher segregation (high s) and higher economic inequality (low λ) exacerbate the outbreak sizes. While due to the different nature of the model, the studied parameter is not the same as the previous models, the same underlying results are further confirmed.

S7 Empirical Analysis of United State Metropolitan Areas

In this section, we provide the technical details of the empirical analysis of section 2 of the main manuscript. We simulate the spreading independently in each metropolitan area (MA) using the mean-field approximation. Each block b corresponds to one ethnic group given that the segregation in cities is strongly correlated with ethnicity. We only focus on the four major ethnic groups (Non-Hispanic White, Black, Hispanic, and Asian) and neglect the rest of the population for the sake of simplicity. Hence, we set the number of blocks to $B = 4$. The population of each block N_b will be set to

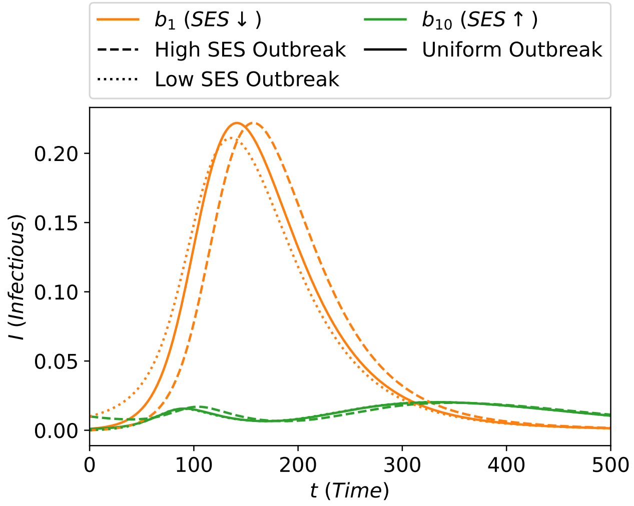

Figure S11. Diversified initial conditions under three scenarios. The solid lines represent the baseline model with initial infectious agents distributed uniformly across the SES groups. The dashed line illustrates the scenario with all infectious agents belonging to the highest SES group (High SES Outbreak). The dotted lines represent the scenario with all infectious agents belonging to the lowest SES group (Low SES Outbreak). Color denotes the SES groups, with orange and green respectively representing the lowest and highest SES blocks.

its corresponding ethnic group's population proportion. Following the argument in the main text, we approximate the level of access to quarantine for each block by the average wealth of the corresponding ethnicity in each metropolitan area. For each MA, we use the empirical exposure indices $\boldsymbol{\phi}$ provided in[68]. We can obtain $\rho_{a,b}$ the connection probability between members of blocks a and b, using $\phi_{a,b} = \frac{\rho_{a,b} N_b}{\langle k \rangle}$ as derived in the S3.1. As we can obtain the contact matrices empirically the parameter s describing the level of segregation becomes obsolete in this section. Additionally, we consider a hypothetical scenario with perfect homogeneity which we call the *desegregated mixing*. In this scenario, each agent $i \in a$ is exposed to the other groups b solely proportional to their population proportions (N_b) and independent of its own block (ethnic group) membership. Therefore, we will have $\phi_{a,b} = N_b$ for all blocks a and b.

The Gini coefficient G of the Pareto distribution is $G = \frac{1}{2\lambda - 1}$, with λ being the Pareto index[77]. Hence, we can find λ for each MA, based on its empirical Gini coefficient given in[70]. The wealth/quarantine access ranking of ethnic groups is adjusted in accordance with the ranking within each MA[70]. The wealth is distributed on the assumption of Pareto distribution with the obtained empirical λ, among the four unequally sized ethnic blocks based on the empirical ordering of the wealth. Due to the redistricting and changes in the definition of some of the MAs, we have missing economical (λ and wealth ranking of ethnicities) parameters. For such MAs, we use the values calculated across the whole country. As with the baseline model, we keep the reproduction number $R_0 = 3$. To better reflect the difference between the behavior of different blocks, we will set $\beta = 5$. The parameter r_d is assumed to be -100 so that the outbreak is in the range between 0.6 and 0.7. However, the robustness of the model with respect to these parameters is explored in S5.

In Fig. 3 of the main manuscript, S, *Entropy* S24, a measure of uncertainty in the system, has been used to quantify the diversity of the population[62]. In the context of ethnic composition, the entropy of the ethnic distribution denotes how unexpected a randomly chosen individual's ethnicity would be. In the case of full homogeneity, where all people are from the same ethnicity, the entropy/uncertainty would be 0. In the case of equally sized populations, the entropy/uncertainty would be maximum. We define $\tilde{S}$, the normalized entropy by dividing the entropy over S_M, the maximum entropy possible in a population with B different ethnic groups. $\tilde{S}$ corresponds to the diversity, the value shown as the color code in Fig. 3 of the main manuscript.

$$
\begin{aligned}
S &= -\sum_{b=1}^{B} N_b \log_2 N_b \\
S_M &= -\sum_{b=1}^{B} \frac{1}{B} \log_2 \frac{1}{B} = \log_2 B \\
\tilde{S} &= \frac{S}{S_M}
\end{aligned}
\tag{S24}
$$

S8 Case study of Covid-19 in Chicago: Ethnic composition of the infection cases

Using our method, we predict the ethnic composition of the Covid-19 cases during the first infection wave in the city of Chicago (not to be confused with the Chicago metropolitan area studied in the previous section). We again limit our analysis to the four major ethnic groups (Non-Hispanic White, Black, Hispanic, and Asian). We use the daily data on Covid-19 cases by race/ethnicity in the city of Chicago[63]. We focus on the first months of the pandemic from the 14th of March 2020 to the 4th of July 2020 (113 days) to avoid more complicated extrinsic effects such as vaccination and mandatory lockdowns.

To run the simulation for this case study, we need to find suitable values for the model parameters in Eq. S16. We use the demographics of Chicago[64] to set the proportional population of each group b, N_b.

In the next step, we empirically inform the segregation matrix, $\boldsymbol{\phi}$, for the city of Chicago. As opposed to the case of the metropolitan areas, we did not find a data set describing the exposure indices of the city of Chicago. Therefore, we calculate this parameter based on the population of each race/ethnicity in each community area (CA) of the city of Chicago[64] using

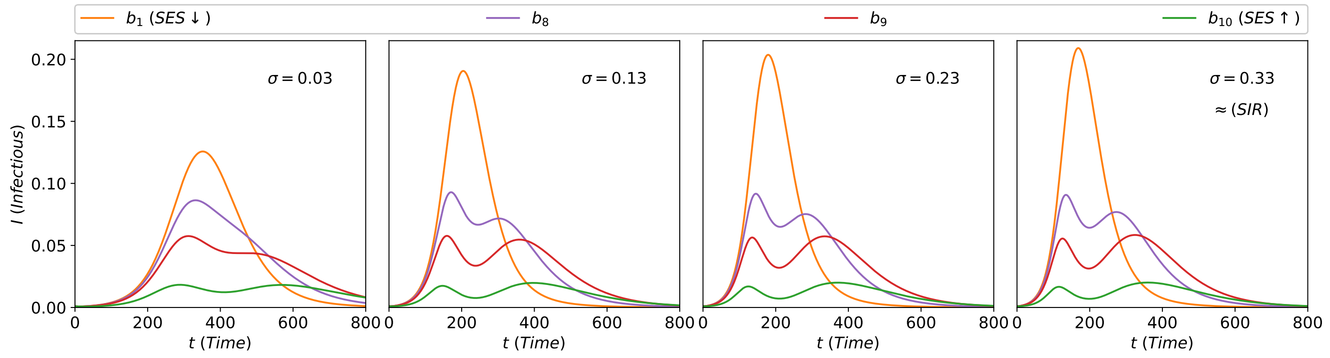

Figure S12. Evolution of $I(t)$, the proportion of infectious agents in each block over time t in the SEIR model. Color code denotes SES groups, where higher SES groups are assigned to higher indices.

a

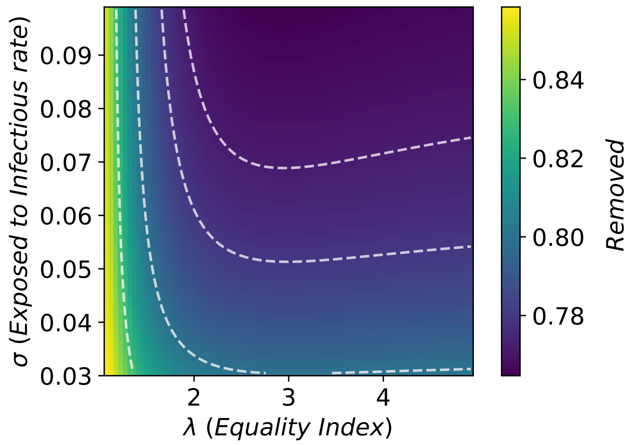

Figure S13. Outbreak size, R in the society for varying levels of exposed-to-infectious rate and equality for the SEIR model. The x-axis is the parameter λ, the indicator of wealth equality. The y-axis σ, is the rate by which exposed agents become infectious. Color-axis represents the proportion of the removed agents R at the end of the dynamics. The results are based on the mean-field approximation. Dashed lines are guides for the eye.

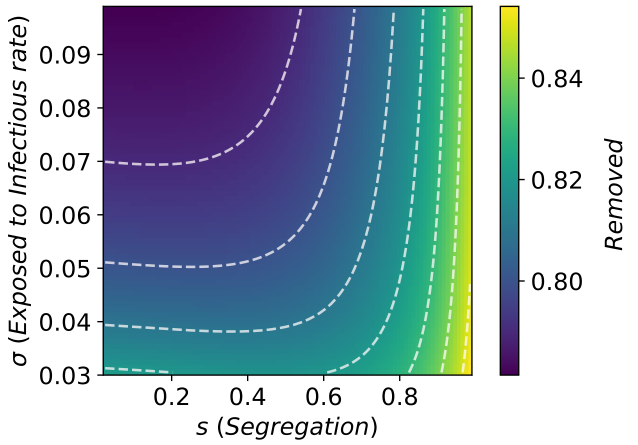

Figure S14. Outbreak size, R in the society for varying levels of exposed-to-infectious rate and segregation for the SEIR model. The x-axis is s, the indicator of segregation. The y-axis σ, is the rate by which exposed agents become infectious. Color-axis represents the proportion of the removed agents R at the end of the dynamics. The results are based on the mean-field approximation. Dashed lines are guides for the eye.

its mathematical definition ($\phi_{a,b} = \sum_i \frac{n_{ia}}{N_a}\frac{n_{ib}}{n_i}$)[62,69]. Where n_{ia} is the population of ethnicity a in neighborhood i, N_a is the overall population of ethnicity a and n_i is the overall population of the neighborhood i. Using the exposure matrix $\boldsymbol{\phi}$ we then obtain the segregation matrix $\boldsymbol{\rho}$ (see section S3.1). Furthermore, similar to section 2, we consider an alternative hypothetical scenario with perfect homogeneity *desegregated mixing*. Therefore, we will have $\phi_{a,b} = N_b$ for all blocks a and b.

Having the population proportions and the segregation patterns, we need to set suitable values for the rest of our parameters: Transmission rate, μ, removal rate, γ, the intensity of selection in the decision-making process, β, fear of infection r_d, and the fear of losing income for each group b, r_b.

For this purpose, we use the maximum likelihood (ML) method to estimate the parameters, given the empirical data of the Covid-19 cases.

Using the ML method, we find the values for our parameters that maximize the *likelihood* of the observed data under the model. Likelihood is defined as the probability for a given set of data $\boldsymbol{Y}$ to occur under a particular model with a set of parameters $\boldsymbol{\theta}$. In other words, we find the values for our parameters by which the observed data is most probable to occur.

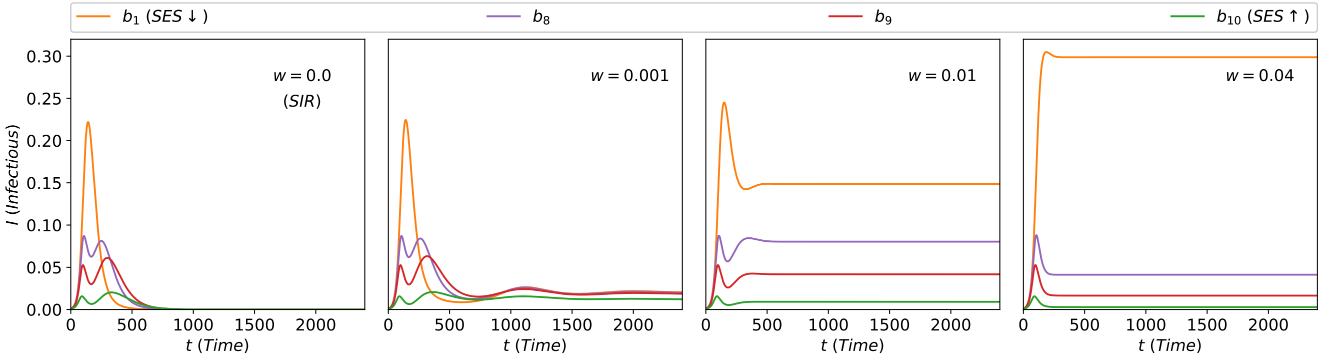

Figure S15. Evolution of $I(t)$, the proportion of infectious agents in each block over time t in the SIRS model. Color code denotes SES groups, where higher SES groups are assigned to higher indices.

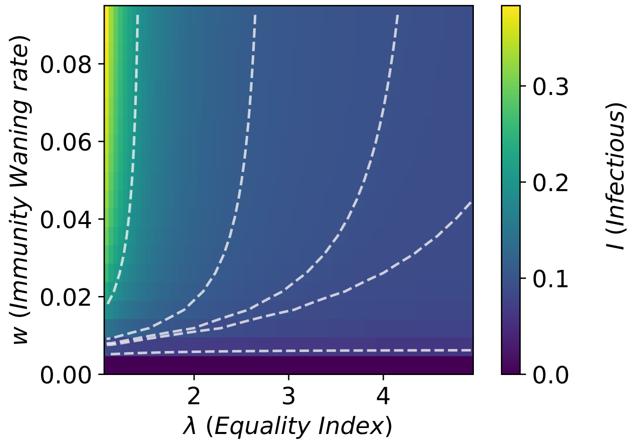

Figure S16. Proportion of the stable infectious population, I in the society for varying levels of immunity loss rate and equality for the SIRS model. The x-axis is the parameter λ, the indicator of wealth equality. The y-axis w, is the immunity loss rate. Color-axis represents the proportion of the removed agents R at the end of the dynamics. The results are based on the mean-field approximation. Dashed lines are guides for the eye.

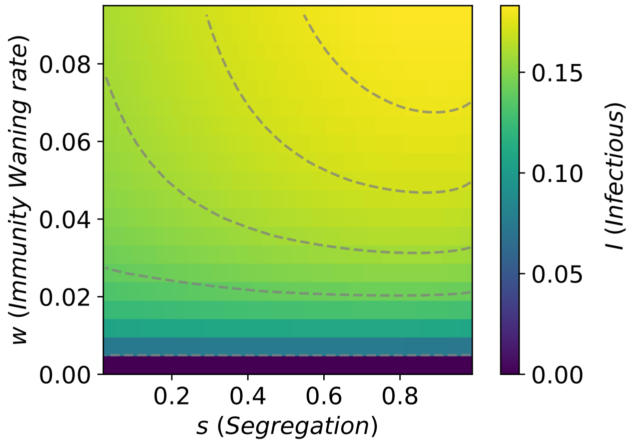

Figure S17. Proportion of the stable infectious population, I in the society for varying levels of immunity loss rate and segregation for the SIRS model. The x-axis is s, the indicator of segregation. The y-axis w, is the immunity loss rate. Color-axis represents the proportion of the removed agents R at the end of the dynamics. The results are based on the mean-field approximation. Dashed lines are guides for the eye.

$$\mathscr{L}(\boldsymbol{\theta};\boldsymbol{Y}) = \Psi(\boldsymbol{Y} \mid \boldsymbol{\theta}) \tag{S25}$$

Assuming the data points to be independent, we have:

$$\mathscr{L}(\boldsymbol{\theta};\boldsymbol{Y}) = \prod_{b=1}^{B}\prod_{t}\Psi(Y_b(t) \mid \boldsymbol{\theta}) \tag{S26}$$

Where $Y_b(t)$ represents the infection cases at time t, for group b.

We, therefore, need $\Psi(\boldsymbol{Y} \mid \boldsymbol{\theta})$, the probability to observe the empirical data, given our model and parameters. We note that Ψ describes the probability of having $Y_b(t)$ cases in each block b at time t given the parameters $\boldsymbol{\theta}$. Where $\boldsymbol{\theta}$ represents $\mu, \gamma, \beta, p_D, r_d$, and r_b for all groups b.

We can presume the expectation value of the function Ψ to lead to I_b in Eq. S16. However, we acknowledge that not all Covid-19 cases are expected to be documented. We, therefore, assume a probability p_D for a case to be symptomatic, identified, and documented. Hence, we devise Ψ in a way that its expectation value is set to $p_D I(t)$. To have a discrete probability distribution without the addition of further parameters, we suppose a Poisson distribution and set $\Psi(\boldsymbol{\theta}) = Poisson(p_D I(t))$.

To simplify the calculations, we turn to maximizing the

logarithm of the likelihood in Eq. S26, as it is equivalent to the maximization of the likelihood itself. We can also write the condition more directly based on the parameter $p_D I(t)$, as in S27.

$$\begin{aligned}\log \mathscr{L}(\boldsymbol{\theta};\boldsymbol{Y}) &= \log \prod_{b=1}^{B} \prod_{t} \Psi(Y_b(t) \mid p_D, I_b(t;\boldsymbol{\theta})) \\ &= \sum_{b=1}^{B} \sum_{t} \log \Psi(Y_b(t) \mid p_D, I_b(t;\boldsymbol{\theta})) \\ &= \sum_{b=1}^{B} \sum_{t} \log \mathit{Poisson}(Y_b(t); p_D I_b(t;\boldsymbol{\theta}))\end{aligned} \tag{S27}$$

The ML estimated parameters would be the set of parameters $\hat{\boldsymbol{\theta}}_{ML}$ that maximize the Log-likelihood:

$$\hat{\boldsymbol{\theta}}_{ML} = \arg\max_{\boldsymbol{\theta}} \{\log \mathscr{L}(\boldsymbol{\theta};\boldsymbol{Y})\} \tag{S28}$$

We use `optim`, a general-purpose optimization function in the R programming language to find $\hat{\boldsymbol{\theta}}_{ML}$ based on the Nelder and Mead method[78].

As mentioned above, two models will be considered, one with the empirical values of segregation and the other with perfect homogeneity or *desegregated mixing*. After obtaining the optimized values $\hat{\boldsymbol{\theta}}_{ML}$, we plug them in the model (Eq. S16).

Fig. 4 shows the result of our fitted models for the two aforementioned scenarios alongside the empirical data. We illustrate the percentage of the daily reported cases of Covid-19 coming from the four major ethnicities in Chicago. The left panel represents the model with empirical segregation, and the right panel represents the model without consideration for segregation. Results indicate that our proposed model (with empirical segregation) is successful in capturing the infection trends across the ethnic groups over time. The agreement between the model and the data becomes strong after the initial two weeks. The initial disagreement could be partially due to the small sample of the infected cases and also the lower accuracy in testing methods and documentation of the cases. It should be noted that traditional spreading models, without considerations for the economic and/or structural inequalities, would not be capable of capturing the ethnic discrepancies and they would predict infection cases proportional to the population of each group.

S9 Data

In the following tables, we provide the empirical data and the simulation results used in Fig. 3 of the main manuscript.

Table S2. Metropolitan Areas, their code numbers and outbreak sizes in the two scenarios.

Code	Metro	Real Outbreak	Desegregated Outbreak
10180	Abilene, TX	0.692	0.688
10420	Akron, OH	0.687	0.683
10500	Albany, GA	0.690	0.662
10540	Albany-Lebanon, OR	0.695	0.695
10580	Albany-Schenectady-Troy, NY	0.675	0.672
10740	Albuquerque, NM	0.684	0.675
10780	Alexandria, LA	0.708	0.692
10900	Allentown-Bethlehem-Easton, PA-NJ	0.691	0.684
11020	Altoona, PA	0.704	0.703
11100	Amarillo, TX	0.689	0.678
11180	Ames, IA	0.695	0.694
11244	Anaheim-Santa Ana-Irvine, CA	0.677	0.655
11260	Anchorage, AK	0.691	0.687
11460	Ann Arbor, MI	0.652	0.648
11500	Anniston-Oxford, AL	0.684	0.679
11540	Appleton, WI	0.696	0.696
11700	Asheville, NC	0.673	0.673
12020	Athens-Clarke County, GA	0.688	0.682
12060	Atlanta-Sandy Springs-Alpharetta, GA	0.684	0.661
12100	Atlantic City-Hammonton, NJ	0.692	0.674
12220	Auburn-Opelika, AL	0.680	0.677
12260	Augusta-Richmond County, GA-SC	0.693	0.682
12420	Austin-Round Rock-Georgetown, TX	0.666	0.658
12540	Bakersfield, CA	0.678	0.651
12580	Baltimore-Columbia-Towson, MD	0.682	0.662
12620	Bangor, ME	0.700	0.700
12700	Barnstable Town, MA	0.697	0.696
12940	Baton Rouge, LA	0.679	0.662
12980	Battle Creek, MI	0.691	0.688
13020	Bay City, MI	0.702	0.702
13140	Beaumont-Port Arthur, TX	0.702	0.673
13220	Beckley, WV	0.696	0.696
13380	Bellingham, WA	0.666	0.665
13460	Bend, OR	0.695	0.695
13740	Billings, MT	0.697	0.696
13780	Binghamton, NY	0.684	0.683
13820	Birmingham-Hoover, AL	0.711	0.689
13900	Bismarck, ND	0.697	0.697
13980	Blacksburg-Christiansburg, VA	0.665	0.661
14010	Bloomington, IL	0.691	0.690
14020	Bloomington, IN	0.694	0.694
14100	Bloomsburg-Berwick, PA	0.697	0.697
14260	Boise City, ID	0.686	0.686
14454	Boston, MA	0.667	0.653
14500	Boulder, CO	0.690	0.689
14540	Bowling Green, KY	0.682	0.680

14740	Bremerton-Silverdale-Port Orchard, WA	0.691	0.691
14860	Bridgeport-Stamford-Norwalk, CT	0.696	0.675
15180	Brownsville-Harlingen, TX	0.668	0.666
15260	Brunswick, GA	0.695	0.685
15380	Buffalo-Cheektowaga, NY	0.691	0.680
15500	Burlington, NC	0.695	0.688
15540	Burlington-South Burlington, VT	0.675	0.674
15680	California-Lexington Park, MD	0.692	0.689
15764	Cambridge-Newton-Framingham, MA	0.693	0.685
15804	Camden, NJ	0.682	0.671
15940	Canton-Massillon, OH	0.701	0.700
15980	Cape Coral-Fort Myers, FL	0.693	0.683
16020	Cape Girardeau, MO-IL	0.696	0.694
16060	Carbondale-Marion, IL	0.695	0.693
16180	Carson City, NV	0.675	0.674
16220	Casper, WY	0.691	0.691
16300	Cedar Rapids, IA	0.692	0.692
16540	Chambersburg-Waynesboro, PA	0.696	0.695
16580	Champaign-Urbana, IL	0.690	0.682
16620	Charleston, WV	0.674	0.671
16700	Charleston-North Charleston, SC	0.688	0.682
16740	Charlotte-Concord-Gastonia, NC-SC	0.691	0.678
16820	Charlottesville, VA	0.689	0.688
16860	Chattanooga, TN-GA	0.701	0.695
16940	Cheyenne, WY	0.692	0.692
16984	Chicago-Naperville-Evanston, IL	0.684	0.655
17020	Chico, CA	0.686	0.685
17140	Cincinnati, OH-KY-IN	0.693	0.686
17300	Clarksville, TN-KY	0.686	0.683
17420	Cleveland, TN	0.669	0.669
17460	Cleveland-Elyria, OH	0.707	0.688
17660	Coeur d'Alene, ID	0.696	0.696
17780	College Station-Bryan, TX	0.681	0.672
17820	Colorado Springs, CO	0.688	0.686
17860	Columbia, MO	0.692	0.691
17900	Columbia, SC	0.697	0.684
17980	Columbus, GA-AL	0.699	0.679
18020	Columbus, IN	0.661	0.660
18140	Columbus, OH	0.682	0.673
18580	Corpus Christi, TX	0.666	0.653
18700	Corvallis, OR	0.691	0.691
18880	Crestview-Fort Walton Beach-Destin, FL	0.690	0.689
19060	Cumberland, MD-WV	0.700	0.699
19124	Dallas-Plano-Irving, TX	0.669	0.651
19140	Dalton, GA	0.700	0.692
19180	Danville, IL	0.704	0.698
19300	Daphne-Fairhope-Foley, AL	0.694	0.693
19340	Davenport-Moline-Rock Island, IA-IL	0.692	0.691
19430	Dayton-Kettering, OH	0.697	0.687
19460	Decatur, AL	0.704	0.697
19500	Decatur, IL	0.700	0.694
19660	Deltona-Daytona Beach-Ormond Beach, FL	0.690	0.687
19740	Denver-Aurora-Lakewood, CO	0.690	0.682
19780	Des Moines-West Des Moines, IA	0.694	0.692
19804	Detroit-Dearborn-Livonia, MI	0.713	0.669

20020	Dothan, AL	0.685	0.680
20100	Dover, DE	0.684	0.682
20220	Dubuque, IA	0.669	0.668
20260	Duluth, MN-WI	0.698	0.698
20500	Durham-Chapel Hill, NC	0.681	0.670
20700	East Stroudsburg, PA	0.687	0.683
20740	Eau Claire, WI	0.697	0.697
20940	El Centro, CA	0.668	0.664
20994	Elgin, IL	0.692	0.677
21060	Elizabethtown-Fort Knox, KY	0.671	0.663
21140	Elkhart-Goshen, IN	0.692	0.688
21300	Elmira, NY	0.697	0.696
21340	El Paso, TX	0.650	0.645
21420	Enid, OK	0.690	0.688
21500	Erie, PA	0.696	0.693
21660	Eugene-Springfield, OR	0.695	0.695
21780	Evansville, IN-KY	0.698	0.697
21820	Fairbanks, AK	0.659	0.658
22020	Fargo, ND-MN	0.689	0.688
22140	Farmington, NM	0.694	0.690
22180	Fayetteville, NC	0.683	0.676
22220	Fayetteville-Springdale-Rogers, AR	0.691	0.687
22380	Flagstaff, AZ	0.692	0.690
22420	Flint, MI	0.710	0.696
22500	Florence, SC	0.689	0.680
22520	Florence-Muscle Shoals, AL	0.694	0.693
22540	Fond du Lac, WI	0.696	0.696
22660	Fort Collins, CO	0.693	0.693
22744	Fort Lauderdale-Pompano Beach-Sunrise, FL	0.674	0.649
22900	Fort Smith, AR-OK	0.693	0.690
23060	Fort Wayne, IN	0.682	0.677
23104	Fort Worth-Arlington-Grapevine, TX	0.686	0.669
23224	Frederick-Gaithersburg-Rockville, MD	0.682	0.665
23420	Fresno, CA	0.664	0.646
23460	Gadsden, AL	0.706	0.698
23540	Gainesville, FL	0.685	0.680
23580	Gainesville, GA	0.693	0.680
23844	Gary, IN	0.714	0.692
23900	Gettysburg, PA	0.696	0.696
24020	Glens Falls, NY	0.679	0.678
24140	Goldsboro, NC	0.681	0.672
24220	Grand Forks, ND-MN	0.682	0.681
24260	Grand Island, NE	0.693	0.687
24300	Grand Junction, CO	0.695	0.695
24340	Grand Rapids-Kentwood, MI	0.690	0.686
24420	Grants Pass, OR	0.695	0.695
24500	Great Falls, MT	0.696	0.696
24540	Greeley, CO	0.688	0.684
24580	Green Bay, WI	0.696	0.694
24660	Greensboro-High Point, NC	0.693	0.676
24780	Greenville, NC	0.691	0.684
24860	Greenville-Anderson, SC	0.694	0.691
25060	Gulfport-Biloxi, MS	0.666	0.662
25180	Hagerstown-Martinsburg, MD-WV	0.694	0.693
25220	Hammond, LA	0.684	0.680

25260	Hanford-Corcoran, CA	0.666	0.656
25420	Harrisburg-Carlisle, PA	0.678	0.672
25500	Harrisonburg, VA	0.692	0.690
25540	Hartford-East Hartford-Middletown, CT	0.698	0.683
25620	Hattiesburg, MS	0.691	0.680
25860	Hickory-Lenoir-Morganton, NC	0.692	0.692
25940	Hilton Head Island-Bluffton, SC	0.688	0.683
25980	Hinesville, GA	0.679	0.675
26140	Homosassa Springs, FL	0.696	0.696
26300	Hot Springs, AR	0.699	0.698
26380	Houma-Thibodaux, LA	0.648	0.638
26420	Houston-The Woodlands-Sugar Land, TX	0.679	0.652
26580	Huntington-Ashland, WV-KY-OH	0.688	0.688
26620	Huntsville, AL	0.692	0.684
26820	Idaho Falls, ID	0.692	0.692
26900	Indianapolis-Carmel-Anderson, IN	0.691	0.680
26980	Iowa City, IA	0.691	0.690
27060	Ithaca, NY	0.689	0.688
27100	Jackson, MI	0.700	0.699
27140	Jackson, MS	0.711	0.683
27180	Jackson, TN	0.686	0.678
27260	Jacksonville, FL	0.692	0.680
27340	Jacksonville, NC	0.681	0.680
27500	Janesville-Beloit, WI	0.694	0.693
27620	Jefferson City, MO	0.670	0.667
27740	Johnson City, TN	0.702	0.702
27780	Johnstown, PA	0.703	0.703
27860	Jonesboro, AR	0.677	0.665
27900	Joplin, MO	0.694	0.694
27980	Kahului-Wailuku-Lahaina, HI	0.668	0.654
28020	Kalamazoo-Portage, MI	0.690	0.687
28100	Kankakee, IL	0.703	0.693
28140	Kansas City, MO-KS	0.689	0.682
28420	Kennewick-Richland, WA	0.686	0.678
28660	Killeen-Temple, TX	0.680	0.670
28700	Kingsport-Bristol, TN-VA	0.705	0.704
28740	Kingston, NY	0.691	0.690
28940	Knoxville, TN	0.676	0.675
29020	Kokomo, IN	0.696	0.696
29100	La Crosse-Onalaska, WI-MN	0.696	0.696
29180	Lafayette, LA	0.699	0.691
29200	Lafayette-West Lafayette, IN	0.691	0.689
29340	Lake Charles, LA	0.706	0.691
29404	Lake County-Kenosha County, IL-WI	0.673	0.660
29420	Lake Havasu City-Kingman, AZ	0.692	0.691
29460	Lakeland-Winter Haven, FL	0.691	0.685
29540	Lancaster, PA	0.692	0.689
29620	Lansing-East Lansing, MI	0.693	0.691
29700	Laredo, TX	0.690	0.690
29740	Las Cruces, NM	0.657	0.645
29820	Las Vegas-Henderson-Paradise, NV	0.652	0.643
29940	Lawrence, KS	0.692	0.692
30020	Lawton, OK	0.677	0.676
30140	Lebanon, PA	0.697	0.694
30300	Lewiston, ID-WA	0.694	0.694

30340	Lewiston-Auburn, ME	0.697	0.696
30460	Lexington-Fayette, KY	0.689	0.687
30620	Lima, OH	0.700	0.698
30700	Lincoln, NE	0.693	0.692
30780	Little Rock-North Little Rock-Conway, AR	0.703	0.689
30860	Logan, UT-ID	0.699	0.699
30980	Longview, TX	0.697	0.692
31020	Longview, WA	0.689	0.689
31084	Los Angeles-Long Beach-Glendale, CA	0.680	0.642
31140	Louisville/Jefferson County, KY-IN	0.695	0.688
31180	Lubbock, TX	0.681	0.670
31340	Lynchburg, VA	0.688	0.686
31420	Macon-Bibb County, GA	0.707	0.685
31460	Madera, CA	0.685	0.656
31540	Madison, WI	0.660	0.658
31700	Manchester-Nashua, NH	0.681	0.680
31740	Manhattan, KS	0.689	0.688
31860	Mankato, MN	0.695	0.694
31900	Mansfield, OH	0.701	0.700
32580	McAllen-Edinburg-Mission, TX	0.677	0.676
32780	Medford, OR	0.690	0.690
32820	Memphis, TN-MS-AR	0.711	0.679
32900	Merced, CA	0.652	0.645
33124	Miami-Miami Beach-Kendall, FL	0.650	0.639
33140	Michigan City-La Porte, IN	0.694	0.692
33220	Midland, MI	0.697	0.697
33260	Midland, TX	0.691	0.680
33340	Milwaukee-Waukesha, WI	0.707	0.683
33460	Minneapolis-St. Paul-Bloomington, MN-WI	0.693	0.689
33540	Missoula, MT	0.681	0.680
33660	Mobile, AL	0.705	0.686
33700	Modesto, CA	0.670	0.661
33740	Monroe, LA	0.702	0.674
33780	Monroe, MI	0.697	0.697
33860	Montgomery, AL	0.702	0.680
33874	Montgomery County-Bucks County-Chester County, PA	0.691	0.690
34060	Morgantown, WV	0.697	0.696
34100	Morristown, TN	0.701	0.700
34580	Mount Vernon-Anacortes, WA	0.691	0.690
34620	Muncie, IN	0.700	0.699
34740	Muskegon, MI	0.706	0.697
34820	Myrtle Beach-Conway-North Myrtle Beach, SC-NC	0.697	0.696
34900	Napa, CA	0.680	0.671
34940	Naples-Marco Island, FL	0.696	0.679
34980	Nashville-Davidson–Murfreesboro–Franklin, TN	0.689	0.684
35004	Nassau County-Suffolk County, NY	0.672	0.658
35084	Newark, NJ-PA	0.707	0.665
35100	New Bern, NC	0.686	0.685
35154	New Brunswick-Lakewood, NJ	0.648	0.637
35300	New Haven-Milford, CT	0.696	0.679
35380	New Orleans-Metairie, LA	0.696	0.668
35614	New York-Jersey City-White Plains, NY-NJ	0.695	0.652
35660	Niles, MI	0.704	0.692
35840	North Port-Sarasota-Bradenton, FL	0.693	0.689
35980	Norwich-New London, CT	0.693	0.690

36084	Oakland-Berkeley-Livermore, CA	0.670	0.651
36100	Ocala, FL	0.695	0.692
36140	Ocean City, NJ	0.695	0.694
36220	Odessa, TX	0.682	0.673
36260	Ogden-Clearfield, UT	0.694	0.693
36420	Oklahoma City, OK	0.684	0.679
36500	Olympia-Lacey-Tumwater, WA	0.653	0.653
36540	Omaha-Council Bluffs, NE-IA	0.685	0.681
36740	Orlando-Kissimmee-Sanford, FL	0.681	0.666
36780	Oshkosh-Neenah, WI	0.695	0.695
36980	Owensboro, KY	0.697	0.696
37100	Oxnard-Thousand Oaks-Ventura, CA	0.688	0.666
37340	Palm Bay-Melbourne-Titusville, FL	0.690	0.688
37460	Panama City, FL	0.663	0.662
37620	Parkersburg-Vienna, WV	0.699	0.699
37860	Pensacola-Ferry Pass-Brent, FL	0.690	0.687
37900	Peoria, IL	0.697	0.692
37964	Philadelphia, PA	0.706	0.662
38060	Phoenix-Mesa-Chandler, AZ	0.690	0.676
38220	Pine Bluff, AR	0.716	0.683
38300	Pittsburgh, PA	0.683	0.680
38340	Pittsfield, MA	0.697	0.697
38540	Pocatello, ID	0.687	0.686
38860	Portland-South Portland, ME	0.695	0.694
38900	Portland-Vancouver-Hillsboro, OR-WA	0.654	0.653
38940	Port St. Lucie, FL	0.689	0.680
39100	Poughkeepsie-Newburgh-Middletown, NY	0.686	0.681
39150	Prescott Valley-Prescott, AZ	0.691	0.691
39300	Providence-Warwick, RI-MA	0.697	0.688
39340	Provo-Orem, UT	0.693	0.693
39380	Pueblo, CO	0.677	0.673
39460	Punta Gorda, FL	0.694	0.694
39540	Racine, WI	0.693	0.687
39580	Raleigh-Cary, NC	0.666	0.660
39660	Rapid City, SD	0.696	0.696
39740	Reading, PA	0.704	0.686
39820	Redding, CA	0.692	0.692
39900	Reno, NV	0.685	0.681
40060	Richmond, VA	0.687	0.673
40140	Riverside-San Bernardino-Ontario, CA	0.668	0.652
40220	Roanoke, VA	0.696	0.691
40340	Rochester, MN	0.663	0.663
40380	Rochester, NY	0.700	0.689
40420	Rockford, IL	0.691	0.685
40484	Rockingham County-Strafford County, NH	0.688	0.688
40580	Rocky Mount, NC	0.694	0.682
40660	Rome, GA	0.691	0.686
40900	Sacramento-Roseville-Folsom, CA	0.683	0.671
40980	Saginaw, MI	0.708	0.694
41060	St. Cloud, MN	0.696	0.694
41100	St. George, UT	0.663	0.662
41140	St. Joseph, MO-KS	0.695	0.695
41180	St. Louis, MO-IL	0.700	0.685
41420	Salem, OR	0.689	0.685
41500	Salinas, CA	0.690	0.649

41540	Salisbury, MD-DE	0.691	0.686
41620	Salt Lake City, UT	0.666	0.664
41660	San Angelo, TX	0.690	0.685
41700	San Antonio-New Braunfels, TX	0.687	0.671
41740	San Diego-Chula Vista-Carlsbad, CA	0.682	0.665
41884	San Francisco-San Mateo-Redwood City, CA	0.671	0.657
41940	San Jose-Sunnyvale-Santa Clara, CA	0.661	0.648
42020	San Luis Obispo-Paso Robles, CA	0.686	0.685
42034	San Rafael, CA	0.689	0.684
42100	Santa Cruz-Watsonville, CA	0.694	0.675
42140	Santa Fe, NM	0.681	0.665
42200	Santa Maria-Santa Barbara, CA	0.681	0.661
42220	Santa Rosa-Petaluma, CA	0.685	0.680
42340	Savannah, GA	0.689	0.675
42540	Scranton–Wilkes-Barre, PA	0.696	0.692
42644	Seattle-Bellevue-Kent, WA	0.683	0.678
42680	Sebastian-Vero Beach, FL	0.692	0.688
42700	Sebring-Avon Park, FL	0.685	0.683
43100	Sheboygan, WI	0.694	0.694
43300	Sherman-Denison, TX	0.690	0.689
43340	Shreveport-Bossier City, LA	0.714	0.690
43420	Sierra Vista-Douglas, AZ	0.690	0.678
43580	Sioux City, IA-NE-SD	0.695	0.687
43620	Sioux Falls, SD	0.696	0.695
43780	South Bend-Mishawaka, IN-MI	0.695	0.690
43900	Spartanburg, SC	0.689	0.684
44060	Spokane-Spokane Valley, WA	0.694	0.694
44100	Springfield, IL	0.695	0.692
44140	Springfield, MA	0.700	0.685
44180	Springfield, MO	0.697	0.696
44220	Springfield, OH	0.684	0.683
44300	State College, PA	0.688	0.686
44420	Staunton, VA	0.695	0.695
44700	Stockton, CA	0.660	0.650
44940	Sumter, SC	0.692	0.682
45060	Syracuse, NY	0.697	0.692
45104	Tacoma-Lakewood, WA	0.686	0.683
45220	Tallahassee, FL	0.692	0.681
45300	Tampa-St. Petersburg-Clearwater, FL	0.689	0.679
45460	Terre Haute, IN	0.697	0.696
45500	Texarkana, TX-AR	0.675	0.672
45540	The Villages, FL	0.698	0.695
45780	Toledo, OH	0.696	0.689
45820	Topeka, KS	0.697	0.694
45940	Trenton-Princeton, NJ	0.671	0.639
46060	Tucson, AZ	0.689	0.677
46140	Tulsa, OK	0.688	0.682
46220	Tuscaloosa, AL	0.704	0.689
46300	Twin Falls, ID	0.690	0.689
46340	Tyler, TX	0.700	0.687
46520	Urban Honolulu, HI	0.649	0.644
46540	Utica-Rome, NY	0.698	0.693
46660	Valdosta, GA	0.700	0.688
46700	Vallejo, CA	0.669	0.661
47020	Victoria, TX	0.676	0.665

47220	Vineland-Bridgeton, NJ	0.683	0.661
47260	Virginia Beach-Norfolk-Newport News, VA-NC	0.689	0.675
47300	Visalia, CA	0.658	0.647
47380	Waco, TX	0.686	0.674
47460	Walla Walla, WA	0.688	0.687
47580	Warner Robins, GA	0.680	0.675
47664	Warren-Troy-Farmington Hills, MI	0.677	0.672
47894	Washington-Arlington-Alexandria, DC-VA-MD-WV	0.669	0.647
47940	Waterloo-Cedar Falls, IA	0.696	0.693
48060	Watertown-Fort Drum, NY	0.694	0.693
48140	Wausau-Weston, WI	0.697	0.696
48260	Weirton-Steubenville, WV-OH	0.697	0.697
48300	Wenatchee, WA	0.686	0.684
48424	West Palm Beach-Boca Raton-Boynton Beach, FL	0.690	0.672
48540	Wheeling, WV-OH	0.705	0.705
48620	Wichita, KS	0.683	0.679
48660	Wichita Falls, TX	0.688	0.685
48700	Williamsport, PA	0.700	0.700
48864	Wilmington, DE-MD-NJ	0.682	0.672
48900	Wilmington, NC	0.698	0.696
49020	Winchester, VA-WV	0.654	0.649
49180	Winston-Salem, NC	0.702	0.691
49340	Worcester, MA-CT	0.678	0.674
49420	Yakima, WA	0.700	0.680
49620	York-Hanover, PA	0.696	0.693
49660	Youngstown-Warren-Boardman, OH-PA	0.705	0.700
49700	Yuba City, CA	0.646	0.643
49740	Yuma, AZ	0.678	0.651

Table S3. Metropolitan Areas, their demographics, diversity indices and Gini coefficients.
* The missing values of the Gini coefficient are replaced by the national value.

Code	Diversity	Gini	Asian Population	Black Population	Hispanic Population	White Population
10180	0.675	0.446	4216	15452	41566	110356
10420	0.504	0.469	30426	99126	16711	536310
10500	0.616	0.466	2135	79861	3924	61005
10540	0.371	0.406	3112	1512	12571	104118
10580	0.562	0.440	55314	88401	53324	670812
10740	0.678	0.461	30242	28703	439138	349193
10780	0.621	0.482	2593	45492	6423	93001
10900	0.623	0.446	31918	57223	157445	598061
11020	0.197	0.451	1320	3729	1708	113016
11100	0.713	0.464	9785	19302	77791	153401
11180	0.411	0.458	7779	4298	5741	104664
11244	0.840	*	777338	68796	1086834	1198655
11260	0.670	0.423	49821	22335	31682	239282
11460	0.672	0.485	40067	49360	20731	252220
11500	0.572	0.458	1762	27054	5010	79519
11540	0.345	0.413	9493	5307	12280	207935
11700	0.428	0.461	8406	24272	40498	381527
12020	0.683	0.520	9415	40912	19508	140539
12060	0.849	0.471	441049	2128962	730470	2661835
12100	0.834	0.477	23720	43028	53713	148858
12220	0.685	0.510	9962	41456	9135	109795
12260	0.711	0.460	18622	222080	37863	318870
12420	0.808	0.454	191547	175134	728021	1133113
12540	0.740	0.467	52640	54322	499158	279600
12580	0.786	0.456	209701	857431	215867	1499155
12620	0.196	0.451	2754	2498	2715	138306
12700	0.315	0.455	4484	9640	7908	194561
12940	0.698	0.478	21306	310912	55184	466937
12980	0.540	0.448	4359	17778	7426	100385
13020	0.292	0.439	894	2964	5930	90811
13140	0.796	0.467	12871	98968	69597	207916
13220	0.302	0.459	1257	8975	1722	99818
13380	0.498	0.430	15331	4570	22825	170385
13460	0.354	0.453	5642	1886	17237	164595
13740	0.290	0.461	3022	2705	10645	154227
13780	0.447	0.464	11336	16100	11402	200368
13820	0.662	0.485	24296	338291	64820	663010
13900	0.268	0.432	2295	3573	4424	114900
13980	0.455	0.477	10385	10338	6364	135313
14010	0.557	0.474	9391	17778	10524	129440
14020	0.431	0.470	10238	7259	6655	132364
14100	0.255	0.460	1825	1787	2627	74936
14260	0.482	0.444	28572	14152	109350	584342
14454	0.755	*	196213	280804	240589	1272240
14500	0.535	0.484	21910	5413	48168	245203
14540	0.522	0.486	9041	15913	10763	139587
14740	0.577	0.420	26214	12056	24184	199020
14860	0.773	0.540	59317	110743	205351	552125
15180	0.266	0.475	3002	1797	376680	37107

15260	0.603	0.480	1827	25743	6893	76364
15380	0.585	0.464	55857	163377	67476	851927
15500	0.721	0.452	3654	36737	24703	102487
15540	0.319	0.441	9702	6909	5793	194816
15680	0.607	0.395	5086	18601	6545	80233
15764	0.677	*	267713	132140	326092	1622343
15804	0.742	*	78705	224068	157777	799139
15940	0.367	0.443	5027	37482	10815	335739
15980	0.655	0.478	16604	62948	173161	490476
16020	0.387	0.444	1783	9531	2469	80529
16060	0.451	0.481	3560	13797	5168	106601
16180	0.614	0.457	2257	1499	14870	37064
16220	0.349	0.435	1231	1448	7169	66614
16300	0.403	0.433	7721	21657	9870	230990
16540	0.368	0.410	2071	7070	10764	132566
16580	0.739	0.495	27116	32703	16913	140880
16620	0.283	0.481	3213	17903	3306	227211
16700	0.675	0.480	23839	193629	60469	500545
16740	0.763	0.475	132152	615167	312184	1537545
16820	0.622	0.483	15847	27194	14760	158797
16860	0.526	0.476	12834	76628	32687	423757
16940	0.501	0.438	2399	3272	15602	75279
16984	0.887	*	644889	1383626	1710337	3427233
17020	0.620	0.495	13995	5622	40112	139651
17140	0.527	0.467	84506	306691	95073	1712872
17300	0.688	0.434	10920	68513	29339	202217
17420	0.394	0.441	1832	6792	8758	104143
17460	0.631	0.485	66033	434939	133862	1407943
17660	0.259	0.414	3581	1273	8756	149107
17780	0.802	0.505	16901	31006	70307	143798
17820	0.662	0.434	38256	55756	131700	501501
17860	0.518	0.461	9950	23995	8582	161327
17900	0.707	0.465	24092	280516	55280	452439
17980	0.741	0.486	10270	141895	24624	145341
18020	0.504	0.430	5863	2685	7257	64395
18140	0.631	0.453	122816	373119	110967	1478968
18580	0.636	0.456	10800	15699	255272	132778
18700	0.510	0.475	8731	1734	8524	72209
18880	0.594	0.445	12798	27506	28765	206915
19060	0.308	0.430	1148	7525	1458	82556
19124	0.915	*	515716	887823	1538201	2074022
19140	0.593	0.442	1857	4954	42830	90039
19180	0.501	0.443	767	11717	3816	56186
19300	0.430	0.457	3476	20446	12686	186495
19340	0.549	0.442	11187	38470	35572	289939
19430	0.549	0.456	26953	143444	28594	592963
19460	0.539	0.451	1635	20553	13272	112952
19500	0.523	0.462	1940	21690	2733	75117
19660	0.624	0.456	17092	74294	94951	463818
19740	0.712	0.449	179074	192876	691711	1814213
19780	0.525	0.431	35992	47833	57905	551774
19804	0.747	*	77377	702766	117649	857132
20020	0.574	0.470	2123	36983	6111	101747
20100	0.709	0.419	5910	51387	13981	104845
20220	0.324	0.455	2252	5160	2977	87349

20260	0.219	0.439	4209	8065	5096	256720
20500	0.810	0.483	37724	171509	84694	342134
20700	0.713	0.426	4947	25903	28558	104716
20740	0.281	0.433	6582	3651	4375	152745
20940	0.363	0.467	2790	4238	153027	16813
20994	0.719	*	34266	51842	210319	438246
21060	0.500	0.444	4566	17286	8307	120296
21140	0.585	0.427	3201	14290	39594	145039
21300	0.398	0.457	1839	7060	2938	69559
21340	0.422	0.467	15331	28547	717387	99313
21420	0.609	0.442	4162	2665	8982	42939
21500	0.447	0.455	7859	24514	12030	219180
21660	0.454	0.462	17329	8561	37780	298507
21780	0.393	0.464	6620	28390	9439	261753
21820	0.562	0.412	5535	5518	7320	63178
22020	0.420	0.448	8713	18538	9193	203866
22140	0.584	0.447	1417	1197	23630	43583
22180	0.803	0.442	18302	183350	66084	230744
22220	0.619	0.476	35567	18040	96185	366402
22380	0.570	0.464	4437	2926	21719	76904
22420	0.552	0.470	6030	87253	16259	283483
22500	0.627	0.480	3119	85122	5679	102842
22520	0.435	0.452	1671	20350	4810	119732
22540	0.336	0.401	1681	3270	6717	90150
22660	0.451	0.443	12940	6216	44662	282581
22744	0.888	*	84745	550704	608703	644230
22900	0.542	0.475	8776	13130	26112	168110
23060	0.611	0.438	21308	52283	33764	301981
23104	0.850	*	155318	398401	697033	1192352
23224	0.916	*	197182	243085	249528	614616
23420	0.791	0.478	120779	51954	540743	271889
23460	0.502	0.455	1218	16504	4895	77731
23540	0.747	0.506	21922	60859	39042	208164
23580	0.693	0.457	5184	16041	57010	120418
23844	0.720	*	12943	137040	122212	429236
23900	0.327	0.394	1461	2393	7790	89945
24020	0.231	0.426	1530	3283	3513	113904
24140	0.756	0.469	2306	37625	14927	60199
24220	0.406	0.451	3561	4945	5656	85293
24260	0.554	0.432	1035	2279	19699	52515
24300	0.429	0.434	3066	1877	23278	120749
24340	0.547	0.440	38384	87719	110671	823508
24420	0.337	0.466	2086	959	7037	72730
24500	0.315	0.460	1911	1928	4012	68794
24540	0.591	0.429	8786	6008	98462	205881
24580	0.430	0.430	10310	11708	27898	263602
24660	0.762	0.473	35252	218955	77289	428212
24780	0.719	0.477	4105	63248	12968	86837
24860	0.612	0.474	23783	155193	78318	647898
25060	0.650	0.462	13337	94946	26388	269136
25180	0.498	0.423	6594	33681	17418	227678
25220	0.631	0.480	1439	41428	7242	79825
25260	0.726	0.414	7545	9796	86607	44361
25420	0.609	0.436	35501	69967	44138	430003
25500	0.537	0.451	4099	7261	19138	102821

25540	0.725	0.465	75492	148114	187902	775080
25620	0.607	0.498	2733	53073	6192	106901
25860	0.515	0.453	12521	28306	31320	283545
25940	0.671	0.480	3818	39621	28490	139760
25980	0.795	0.404	2764	34163	9765	32778
26140	0.348	0.454	3152	5238	9241	131477
26300	0.479	0.456	1512	9820	7047	77239
26380	0.577	0.478	2778	38504	13030	139524
26420	0.912	0.484	642476	1279987	2669503	2399789
26580	0.204	0.474	3929	12362	4595	329883
26620	0.663	0.459	17309	112365	32184	313189
26820	0.386	0.424	2982	1371	20484	128709
26900	0.654	0.466	96398	348408	177787	1439031
26980	0.547	0.479	10458	14928	11810	134449
27060	0.593	0.495	12239	6074	7077	76737
27100	0.406	0.447	2203	15737	6184	131097
27140	0.630	0.480	9630	288140	16433	269917
27180	0.599	0.457	2065	50936	8187	115721
27260	0.746	0.465	84725	360534	163852	953455
27340	0.707	0.406	8237	32119	27641	129499
27500	0.472	0.418	3001	10904	15800	129796
27620	0.362	0.418	2060	12429	4615	126303
27740	0.310	0.476	3180	8614	8424	180571
27780	0.262	0.436	1114	7597	2465	119380
27860	0.542	0.490	2274	22343	7556	98300
27900	0.399	0.456	4587	5045	14984	143939
27980	0.715	0.441	89737	2448	16914	51926
28020	0.555	0.468	9103	36145	14776	193428
28100	0.620	0.431	1485	17253	12520	73591
28140	0.645	0.452	90101	296834	229233	1501275
28420	0.647	0.426	12373	6569	101784	172943
28660	0.857	0.435	23288	106376	114128	218125
28700	0.204	0.463	2963	8490	6038	281696
28740	0.539	0.462	5035	13277	21119	136695
28940	0.409	0.471	20195	60443	44667	727071
29020	0.412	0.415	1440	8269	3060	68470
29100	0.306	0.448	6582	3580	3294	123147
29180	0.628	0.482	10854	123809	25496	310101
29200	0.586	0.469	18324	13914	20292	165999
29340	0.629	0.472	4883	58598	11581	142284
29404	0.751	*	70922	68045	196508	530285
29420	0.462	0.448	4684	3381	34126	160165
29460	0.763	0.451	16658	110482	187695	392621
29540	0.481	0.426	16841	25461	61171	440613
29620	0.549	0.448	25025	54401	36913	408905
29700	0.151	0.462	1441	895	254354	9495
29740	0.547	0.484	3544	4149	147672	59749
29820	0.924	0.468	301029	320315	701416	892802
29940	0.510	0.463	6110	7533	8295	90281
30020	0.767	0.456	5688	23277	17006	68563
30140	0.450	0.411	2829	3676	20348	114004
30300	0.242	0.437	1160	698	2545	54909
30340	0.312	0.430	1554	7715	2222	95875
30460	0.581	0.477	18864	66184	40213	379636
30620	0.456	0.433	1236	15309	3272	79691

30700	0.499	0.444	17445	19116	26340	268344
30780	0.659	0.476	18433	183707	52786	469142
30860	0.394	0.420	4431	1716	16099	122077
30980	0.698	0.463	3464	53449	47444	173398
31020	0.404	0.445	3610	1678	10802	88067
31084	0.874	*	1635790	862757	4804763	2563609
31140	0.590	0.460	40308	210967	82964	918741
31180	0.755	0.484	11107	28425	114351	160030
31340	0.514	0.430	4834	47132	8932	193788
31420	0.654	0.501	4456	106979	8466	110168
31460	0.636	0.427	4716	4847	93178	48399
31540	0.510	0.438	43709	40103	46048	535921
31700	0.445	0.428	20162	13849	33498	342652
31740	0.636	0.443	7209	14063	14675	93829
31860	0.412	0.441	3172	6657	5021	86509
31900	0.365	0.428	1260	13484	2615	104231
32580	0.229	0.484	9236	3896	800001	53338
32780	0.456	0.466	7055	3398	30399	170232
32820	0.729	0.493	38846	627912	94948	552793
32900	0.696	0.461	22715	10284	173857	68729
33124	0.634	*	48173	396430	1856938	361517
33140	0.498	0.422	1174	13886	7985	85957
33220	0.269	0.464	2293	1667	2659	73825
33260	0.720	0.467	4676	11660	75586	79267
33340	0.712	0.477	77577	272794	182777	1009707
33460	0.619	0.445	305997	392110	242621	2648247
33540	0.289	0.470	2798	1535	6004	99953
33660	0.644	0.473	10523	154600	13563	236970
33700	0.750	0.440	44745	18794	265978	207908
33740	0.629	0.510	3035	78399	7174	113935
33780	0.285	0.419	1602	5802	6231	135369
33860	0.685	0.473	11379	171506	15734	180283
33874	0.565	*	156555	157274	137824	1545295
34060	0.365	0.503	4943	7947	4003	119307
34100	0.367	0.444	1698	4448	12648	119920
34580	0.518	0.429	4918	1647	23792	92347
34620	0.389	0.467	1955	10301	3533	93333
34740	0.516	0.434	1959	26722	10283	130141
34820	0.498	0.446	8234	57381	31724	376445
34900	0.741	0.479	13083	3279	48829	68909
34940	0.648	0.533	7040	24650	102249	235455
34980	0.650	0.463	77076	309616	193873	1359362
35004	0.771	*	251423	284017	589384	1746784
35084	0.879	*	164328	491765	514491	1055834
35100	0.653	0.457	4232	26806	8085	79824
35154	0.777	*	361999	187320	398125	1491972
35300	0.764	0.472	42680	123457	170081	509688
35380	0.785	0.499	44693	437140	146939	614816
35614	0.961	*	1914434	2296103	3565627	4417586
35660	0.563	0.476	4155	23727	9210	111603
35840	0.548	0.490	21805	55045	114215	622801
35980	0.591	0.443	13651	19825	30920	194894
36084	0.949	*	853816	299440	708649	927698
36100	0.641	0.460	7940	47611	55910	253837
36140	0.386	0.476	1337	4291	7465	80040

36220	0.641	0.451	3000	8311	100051	51023
36260	0.465	0.389	25444	12519	93912	547270
36420	0.714	0.464	60970	176369	212546	845718
36500	0.607	0.406	28385	14580	29024	207985
36540	0.604	0.445	42976	88653	111481	699143
36740	0.854	0.461	146254	425302	856028	1162010
36780	0.380	0.442	6649	6663	8328	145581
36980	0.359	0.459	2874	7214	4471	104604
37100	0.748	0.446	79507	19674	365285	360850
37340	0.611	0.453	22265	66244	67907	430936
37460	0.571	0.437	6343	20320	13846	128348
37620	0.174	0.455	913	2045	1196	82717
37860	0.626	0.441	21434	87377	32683	348480
37900	0.455	0.454	11342	42917	15185	322899
37964	0.867	*	185945	779245	265049	914077
38060	0.751	0.462	269642	325401	1472948	2597577
38220	0.614	0.471	993	43133	2289	39847
38300	0.419	0.466	82122	233234	52920	1947870
38340	0.371	0.460	2792	6133	7064	109263
38540	0.404	0.437	2470	1298	10617	75273
38860	0.276	0.437	14500	18212	12757	490011
38900	0.638	0.444	249060	105725	332155	1726583
38940	0.716	0.479	10555	78564	90507	295749
39100	0.715	0.452	27090	82107	131968	430343
39150	0.426	0.450	4844	2693	34390	183296
39300	0.595	0.464	63589	121164	236852	1199708
39340	0.462	0.418	30453	7922	89105	529241
39380	0.622	0.458	2589	4319	69921	85527
39460	0.421	0.457	3567	10664	14079	153700
39540	0.628	0.444	3333	26397	27911	135333
39580	0.788	0.447	113584	275383	169603	823769
39660	0.329	0.448	3137	3222	6876	108037
39740	0.598	0.437	8116	22599	99550	291258
39820	0.470	0.463	8535	3469	19730	136894
39900	0.705	0.462	40776	16616	122544	291200
40060	0.753	0.473	68844	383986	104488	726834
40140	0.809	0.448	406011	371876	2373208	1354348
40220	0.530	0.464	8785	45234	15863	237907
40340	0.464	0.435	12855	13578	11302	183659
40380	0.591	0.458	42853	137499	88854	794477
40420	0.687	0.449	10864	45514	53832	219924
40484	0.264	*	14329	6544	14171	397698
40580	0.683	0.461	1346	66314	10028	63657
40660	0.623	0.479	1691	15303	11466	67747
40900	0.872	0.458	431485	200469	533221	1156794
40980	0.621	0.469	3114	36752	16904	128722
41060	0.421	0.424	4828	17537	8167	165097
41100	0.401	0.436	5659	1869	20482	147462
41140	0.385	0.441	2207	7346	6422	101414
41180	0.581	0.464	104268	545323	106269	1983321
41420	0.601	0.434	18708	7868	108470	278369
41500	0.688	0.455	33583	12135	265321	120077
41540	0.623	0.459	9154	75466	37037	286350
41620	0.620	0.428	93528	31367	241610	859113
41660	0.661	0.452	2561	5061	47864	64369

41700	0.730	0.464	94121	190050	1389685	838674
41740	0.851	0.462	495274	184697	1119629	1422205
41884	0.868	*	584391	75572	328147	617208
41940	0.850	0.466	811305	55155	526598	575493
42020	0.620	0.444	15303	6156	67921	183468
42034	0.659	*	23652	8432	49410	173149
42100	0.687	0.473	17877	4884	94299	145551
42140	0.607	0.484	3326	1825	74377	67861
42200	0.717	0.479	32840	9520	210584	184746
42220	0.678	0.451	32764	11369	141438	285792
42340	0.736	0.468	16049	130027	30551	218958
42540	0.478	0.453	13188	27649	65746	449692
42644	0.785	*	670168	224429	338665	1759003
42680	0.564	0.517	3081	14593	20832	117422
42700	0.662	0.497	1959	10580	20943	65511
43100	0.456	0.406	7599	3401	8662	95837
43300	0.568	0.440	2657	9328	20868	95211
43340	0.677	0.558	7813	160308	19380	195831
43420	0.690	0.435	4327	5905	42615	68256
43580	0.625	0.432	5416	8866	29014	101221
43620	0.402	0.421	7457	15961	14137	227569
43780	0.595	0.472	9218	44860	28737	231865
43900	0.662	0.458	9808	68804	27732	214440
44060	0.417	0.454	26477	18777	37035	470528
44100	0.476	0.452	5496	30267	5469	161969
44140	0.655	0.469	29431	49756	136533	467506
44180	0.324	0.456	11013	17383	19305	405529
44220	0.423	0.427	1563	15403	5313	109794
44300	0.442	0.471	13327	6417	5651	129668
44420	0.395	0.427	1598	10612	5761	104408
44700	0.907	0.451	153600	66993	325725	215530
44940	0.656	0.464	2482	64604	5209	61751
45060	0.500	0.454	25933	68052	31861	513965
45104	0.747	*	109182	89917	111811	569815
45220	0.736	0.484	13891	125380	30242	206258
45300	0.752	0.477	150922	401879	652022	1888689
45460	0.305	0.441	3030	10641	4426	161894
45500	0.631	0.482	1877	38782	9594	92041
45540	0.390	0.425	1576	8962	7583	109213
45780	0.566	0.470	13527	99828	46297	470560
45820	0.524	0.427	4125	18904	25832	173646
45940	0.929	0.504	51708	77294	84177	168580
46060	0.706	0.466	42718	46405	372788	536868
46140	0.658	0.473	36760	101638	118902	599134
46220	0.654	0.471	4477	93678	13561	152203
46300	0.504	0.434	2549	1505	24386	82322
46340	0.744	0.461	5208	40655	47281	134452
46520	0.639	0.433	703490	32936	92322	175530
46540	0.464	0.436	12472	19590	16721	235637
46660	0.702	0.514	2993	54353	10490	77295
46700	0.967	0.415	87608	71017	128155	155125
47020	0.692	0.466	1680	6101	45219	43576
47220	0.804	0.500	2475	29172	53054	65808
47260	0.765	0.443	99873	578741	134850	941693
47300	0.591	0.460	19447	7172	309895	125022

47380	0.768	0.483	6569	43109	72552	148400
47460	0.566	0.448	1910	1449	14206	42580
47580	0.740	0.433	6954	67792	14354	98330
47664	0.557	*	168922	314936	102304	1942401
47894	0.924	*	606421	1415630	845422	2088703
47940	0.459	0.450	5384	16613	7104	136344
48060	0.461	0.432	3231	8204	7990	92755
48140	0.314	0.427	9585	2424	4982	145517
48260	0.252	0.435	920	6474	1870	103821
48300	0.545	0.448	2485	860	36741	77538
48424	0.801	*	52199	273743	349933	779759
48540	0.213	0.466	1130	5796	1825	126764
48620	0.647	0.448	29605	59809	91517	439813
48660	0.666	0.458	3859	15655	27186	95991
48700	0.303	0.423	1415	7655	2374	99687
48864	0.758	*	41989	173272	75501	432029
48900	0.553	0.491	6086	38280	22282	211568
49020	0.498	0.415	3330	8733	15821	111017
49180	0.666	0.469	15555	124421	77803	442388
49340	0.595	0.451	55493	57222	126361	703051
49420	0.597	0.432	4668	3034	130049	103578
49620	0.466	0.411	9068	32216	39360	365353
49660	0.425	0.457	5751	65542	19881	435715
49700	0.821	0.444	27254	6968	55088	83116
49740	0.574	0.454	3502	4400	130003	61123

Table S4. Exposure Indices for Metropolitan areas, values denote how much the first ethnicity is exposed to the second. w, b, a and h respectively denote White, Black, Asian and non-white Hispanic ethnicities.

Code	w_a	w_b	w_h	w_w	b_a	b_b	b_h	b_w	h_a	h_b	h_h	h_w	a_a	a_b	a_h	a_w
10180	0.64	0.54	0.55	0.69	0.10	0.14	0.11	0.08	0.22	0.29	0.32	0.21	0.04	0.03	0.02	0.02
10420	0.70	0.55	0.73	0.84	0.15	0.37	0.18	0.10	0.03	0.03	0.03	0.02	0.12	0.05	0.06	0.04
10500	0.54	0.27	0.43	0.59	0.40	0.69	0.52	0.36	0.03	0.02	0.04	0.03	0.03	0.01	0.02	0.02
10540	0.84	0.84	0.82	0.86	0.01	0.01	0.01	0.01	0.11	0.12	0.13	0.10	0.03	0.03	0.03	0.02
10580	0.69	0.52	0.65	0.82	0.12	0.29	0.18	0.07	0.07	0.11	0.09	0.05	0.11	0.08	0.08	0.06
10740	0.47	0.41	0.35	0.49	0.04	0.05	0.03	0.03	0.42	0.50	0.59	0.44	0.06	0.04	0.03	0.04
10780	0.66	0.38	0.62	0.75	0.25	0.57	0.24	0.19	0.05	0.03	0.12	0.04	0.03	0.01	0.02	0.02
10900	0.70	0.57	0.49	0.78	0.07	0.11	0.10	0.06	0.15	0.28	0.38	0.13	0.07	0.04	0.03	0.04
11020	0.94	0.91	0.93	0.94	0.03	0.06	0.04	0.03	0.02	0.02	0.02	0.01	0.02	0.01	0.01	0.01
11100	0.42	0.42	0.46	0.68	0.10	0.17	0.09	0.05	0.32	0.36	0.41	0.24	0.17	0.05	0.04	0.03
11180	0.76	0.80	0.82	0.86	0.05	0.05	0.04	0.03	0.06	0.06	0.06	0.04	0.13	0.10	0.08	0.06
11244	0.33	0.37	0.26	0.52	0.02	0.03	0.02	0.02	0.27	0.34	0.52	0.24	0.37	0.26	0.20	0.22
11260	0.59	0.58	0.64	0.74	0.09	0.10	0.08	0.05	0.11	0.12	0.11	0.08	0.21	0.20	0.17	0.12
11460	0.63	0.55	0.65	0.74	0.10	0.30	0.16	0.11	0.06	0.07	0.07	0.05	0.21	0.08	0.11	0.10
11500	0.64	0.52	0.68	0.76	0.28	0.42	0.24	0.18	0.05	0.04	0.06	0.04	0.02	0.02	0.02	0.01
11540	0.84	0.84	0.86	0.89	0.03	0.04	0.03	0.02	0.06	0.07	0.07	0.05	0.06	0.06	0.05	0.04
11700	0.82	0.76	0.78	0.85	0.06	0.12	0.06	0.05	0.10	0.10	0.14	0.08	0.02	0.02	0.02	0.02
12020	0.70	0.53	0.55	0.72	0.15	0.32	0.25	0.15	0.07	0.12	0.17	0.08	0.08	0.04	0.04	0.05
12060	0.44	0.25	0.36	0.62	0.23	0.59	0.31	0.20	0.14	0.11	0.24	0.10	0.19	0.05	0.08	0.07
12100	0.45	0.34	0.41	0.68	0.14	0.34	0.20	0.10	0.23	0.25	0.28	0.15	0.18	0.08	0.10	0.07
12220	0.64	0.55	0.58	0.69	0.20	0.35	0.26	0.21	0.05	0.06	0.10	0.05	0.11	0.05	0.06	0.06
12260	0.59	0.40	0.54	0.62	0.28	0.52	0.34	0.28	0.07	0.06	0.09	0.06	0.06	0.02	0.04	0.03
12420	0.51	0.42	0.41	0.59	0.08	0.13	0.09	0.06	0.24	0.38	0.44	0.26	0.17	0.08	0.06	0.09
12540	0.32	0.28	0.21	0.50	0.06	0.12	0.06	0.06	0.48	0.55	0.68	0.39	0.14	0.06	0.05	0.06
12580	0.53	0.29	0.48	0.69	0.23	0.58	0.29	0.17	0.08	0.07	0.15	0.07	0.16	0.06	0.07	0.07
12620	0.92	0.93	0.94	0.95	0.02	0.02	0.02	0.02	0.02	0.02	0.02	0.02	0.03	0.02	0.02	0.02
12700	0.88	0.85	0.85	0.91	0.05	0.07	0.07	0.04	0.04	0.06	0.06	0.03	0.02	0.02	0.02	0.02
12940	0.57	0.34	0.53	0.69	0.29	0.59	0.34	0.22	0.08	0.06	0.11	0.06	0.06	0.02	0.03	0.03
12980	0.75	0.59	0.70	0.81	0.10	0.30	0.19	0.10	0.06	0.08	0.08	0.05	0.09	0.03	0.03	0.03
13020	0.90	0.86	0.87	0.91	0.03	0.05	0.04	0.03	0.06	0.08	0.08	0.06	0.01	0.01	0.01	0.01
13140	0.46	0.26	0.37	0.72	0.26	0.50	0.29	0.13	0.20	0.20	0.30	0.13	0.07	0.03	0.04	0.03
13220	0.86	0.79	0.86	0.90	0.10	0.17	0.11	0.07	0.02	0.02	0.02	0.02	0.03	0.01	0.01	0.01
13380	0.78	0.78	0.78	0.81	0.02	0.03	0.02	0.02	0.11	0.11	0.13	0.10	0.09	0.08	0.07	0.07
13460	0.86	0.85	0.84	0.87	0.01	0.01	0.01	0.01	0.09	0.10	0.11	0.09	0.03	0.03	0.03	0.03
13740	0.90	0.88	0.88	0.91	0.02	0.02	0.02	0.02	0.06	0.08	0.08	0.06	0.02	0.02	0.02	0.02
13780	0.72	0.72	0.75	0.86	0.09	0.14	0.11	0.06	0.07	0.08	0.07	0.04	0.12	0.06	0.07	0.04
13820	0.67	0.32	0.57	0.75	0.21	0.61	0.30	0.16	0.06	0.06	0.11	0.06	0.06	0.02	0.02	0.02
13900	0.91	0.90	0.91	0.92	0.03	0.04	0.03	0.03	0.04	0.04	0.04	0.04	0.02	0.02	0.02	0.02
13980	0.72	0.80	0.79	0.85	0.06	0.10	0.07	0.06	0.06	0.04	0.05	0.04	0.17	0.06	0.09	0.06
14010	0.73	0.70	0.72	0.79	0.09	0.17	0.14	0.10	0.05	0.08	0.09	0.06	0.12	0.05	0.05	0.05
14020	0.77	0.80	0.80	0.86	0.06	0.07	0.06	0.04	0.05	0.06	0.05	0.04	0.12	0.08	0.08	0.06
14100	0.88	0.89	0.90	0.93	0.03	0.03	0.03	0.02	0.04	0.04	0.04	0.03	0.05	0.03	0.03	0.02
14260	0.80	0.78	0.73	0.81	0.02	0.03	0.02	0.02	0.12	0.14	0.22	0.14	0.05	0.04	0.03	0.04
14454	0.58	0.34	0.40	0.76	0.11	0.40	0.22	0.07	0.10	0.19	0.30	0.08	0.21	0.08	0.08	0.09
14500	0.76	0.75	0.69	0.78	0.02	0.02	0.02	0.02	0.12	0.16	0.24	0.14	0.09	0.07	0.06	0.07
14540	0.66	0.65	0.67	0.83	0.13	0.17	0.15	0.07	0.08	0.10	0.11	0.05	0.12	0.07	0.07	0.04
14740	0.72	0.69	0.72	0.77	0.05	0.07	0.06	0.04	0.10	0.12	0.11	0.09	0.12	0.11	0.11	0.10
14860	0.63	0.32	0.38	0.73	0.09	0.26	0.20	0.06	0.19	0.36	0.36	0.14	0.08	0.05	0.06	0.07
15180	0.14	0.15	0.08	0.18	0.01	0.01	0.00	0.01	0.83	0.83	0.91	0.80	0.02	0.01	0.01	0.01

15260	0.67	0.48	0.57	0.77	0.23	0.42	0.32	0.16	0.07	0.08	0.09	0.05	0.03	0.02	0.02	0.02
15380	0.57	0.38	0.58	0.84	0.21	0.46	0.22	0.07	0.08	0.09	0.13	0.05	0.14	0.07	0.07	0.04
15500	0.62	0.50	0.48	0.68	0.22	0.29	0.28	0.18	0.13	0.19	0.22	0.12	0.03	0.02	0.02	0.02
15540	0.85	0.84	0.88	0.90	0.05	0.06	0.04	0.03	0.03	0.03	0.03	0.03	0.07	0.07	0.05	0.04
15680	0.67	0.61	0.64	0.76	0.20	0.26	0.22	0.14	0.07	0.08	0.08	0.05	0.06	0.05	0.06	0.04
15764	0.63	0.58	0.45	0.76	0.07	0.10	0.07	0.05	0.10	0.18	0.39	0.09	0.20	0.14	0.09	0.10
15804	0.63	0.43	0.43	0.73	0.15	0.34	0.25	0.12	0.11	0.17	0.27	0.08	0.11	0.05	0.05	0.06
15940	0.87	0.70	0.80	0.88	0.08	0.25	0.15	0.08	0.03	0.04	0.04	0.03	0.02	0.01	0.01	0.01
15980	0.68	0.40	0.51	0.74	0.07	0.24	0.12	0.05	0.22	0.33	0.35	0.18	0.03	0.02	0.02	0.02
16020	0.82	0.67	0.80	0.88	0.12	0.27	0.14	0.08	0.03	0.04	0.04	0.02	0.03	0.02	0.02	0.02
16060	0.69	0.64	0.72	0.86	0.18	0.25	0.18	0.08	0.06	0.07	0.06	0.04	0.08	0.05	0.04	0.02
16180	0.68	0.66	0.60	0.69	0.03	0.05	0.02	0.03	0.26	0.25	0.34	0.24	0.04	0.04	0.04	0.04
16220	0.87	0.86	0.86	0.87	0.02	0.02	0.02	0.02	0.09	0.10	0.10	0.09	0.02	0.02	0.02	0.02
16300	0.80	0.76	0.81	0.87	0.10	0.15	0.11	0.07	0.04	0.05	0.05	0.04	0.06	0.03	0.03	0.03
16540	0.84	0.78	0.75	0.88	0.06	0.08	0.08	0.04	0.09	0.13	0.16	0.06	0.02	0.02	0.02	0.01
16580	0.49	0.49	0.53	0.73	0.14	0.29	0.20	0.12	0.09	0.10	0.12	0.06	0.28	0.12	0.14	0.10
16620	0.86	0.76	0.86	0.92	0.09	0.21	0.10	0.06	0.02	0.02	0.02	0.01	0.03	0.02	0.02	0.01
16700	0.64	0.51	0.55	0.70	0.23	0.37	0.28	0.20	0.08	0.09	0.14	0.07	0.04	0.03	0.03	0.03
16740	0.53	0.41	0.46	0.69	0.23	0.39	0.29	0.16	0.12	0.15	0.20	0.09	0.11	0.05	0.05	0.05
16820	0.61	0.67	0.67	0.76	0.13	0.17	0.14	0.12	0.08	0.08	0.10	0.06	0.18	0.08	0.09	0.06
16860	0.77	0.49	0.64	0.84	0.12	0.41	0.19	0.09	0.06	0.08	0.14	0.05	0.05	0.02	0.02	0.02
16940	0.78	0.74	0.74	0.79	0.04	0.05	0.04	0.03	0.16	0.19	0.19	0.15	0.03	0.03	0.02	0.02
16984	0.54	0.18	0.33	0.66	0.08	0.63	0.12	0.07	0.17	0.15	0.48	0.17	0.21	0.04	0.06	0.10
17020	0.66	0.67	0.66	0.72	0.03	0.04	0.03	0.03	0.20	0.21	0.24	0.19	0.10	0.08	0.07	0.07
17140	0.73	0.48	0.68	0.84	0.12	0.43	0.19	0.09	0.05	0.06	0.09	0.04	0.10	0.03	0.04	0.04
17300	0.60	0.55	0.58	0.70	0.24	0.31	0.26	0.19	0.12	0.11	0.12	0.08	0.05	0.04	0.04	0.03
17420	0.83	0.78	0.79	0.87	0.06	0.09	0.08	0.05	0.08	0.11	0.11	0.07	0.02	0.02	0.02	0.02
17460	0.71	0.30	0.59	0.82	0.14	0.62	0.20	0.09	0.06	0.06	0.18	0.06	0.09	0.02	0.03	0.03
17660	0.91	0.91	0.91	0.92	0.01	0.01	0.01	0.01	0.06	0.06	0.06	0.05	0.02	0.02	0.02	0.02
17780	0.57	0.45	0.44	0.62	0.09	0.18	0.14	0.10	0.22	0.32	0.36	0.22	0.12	0.05	0.05	0.07
17820	0.68	0.61	0.62	0.72	0.08	0.11	0.10	0.07	0.18	0.23	0.23	0.16	0.06	0.06	0.05	0.05
17860	0.75	0.71	0.74	0.81	0.13	0.18	0.15	0.11	0.05	0.05	0.06	0.04	0.07	0.05	0.06	0.05
17900	0.57	0.39	0.53	0.66	0.31	0.52	0.33	0.24	0.07	0.06	0.10	0.06	0.05	0.03	0.03	0.03
17980	0.54	0.30	0.44	0.59	0.31	0.60	0.40	0.30	0.10	0.07	0.11	0.08	0.06	0.02	0.04	0.04
18020	0.72	0.78	0.76	0.82	0.04	0.04	0.04	0.03	0.09	0.10	0.13	0.09	0.15	0.08	0.07	0.07
18140	0.66	0.44	0.58	0.79	0.16	0.43	0.26	0.11	0.06	0.08	0.09	0.04	0.13	0.05	0.06	0.06
18580	0.39	0.29	0.26	0.43	0.04	0.07	0.04	0.03	0.52	0.62	0.68	0.51	0.05	0.03	0.02	0.03
18700	0.76	0.77	0.77	0.80	0.02	0.02	0.02	0.02	0.09	0.10	0.11	0.09	0.12	0.11	0.10	0.09
18880	0.72	0.66	0.70	0.77	0.11	0.16	0.12	0.09	0.12	0.12	0.13	0.10	0.06	0.05	0.05	0.04
19060	0.88	0.73	0.84	0.91	0.08	0.23	0.12	0.07	0.02	0.02	0.02	0.02	0.02	0.01	0.01	0.01
19124	0.42	0.28	0.29	0.55	0.13	0.34	0.18	0.12	0.18	0.30	0.47	0.22	0.27	0.08	0.06	0.10
19140	0.64	0.54	0.49	0.72	0.04	0.05	0.05	0.03	0.29	0.39	0.46	0.23	0.02	0.02	0.01	0.01
19180	0.77	0.51	0.68	0.84	0.16	0.40	0.24	0.11	0.05	0.08	0.08	0.05	0.02	0.01	0.01	0.01
19300	0.83	0.76	0.82	0.84	0.09	0.17	0.09	0.08	0.06	0.05	0.08	0.06	0.02	0.02	0.02	0.02
19340	0.73	0.64	0.68	0.80	0.12	0.20	0.13	0.08	0.09	0.12	0.15	0.08	0.06	0.04	0.03	0.03
19430	0.78	0.44	0.73	0.82	0.11	0.50	0.17	0.11	0.04	0.03	0.06	0.04	0.07	0.02	0.04	0.04
19460	0.75	0.53	0.56	0.82	0.14	0.30	0.24	0.10	0.09	0.16	0.19	0.07	0.02	0.01	0.01	0.01
19500	0.76	0.54	0.70	0.80	0.16	0.41	0.25	0.16	0.03	0.03	0.03	0.02	0.05	0.01	0.02	0.02
19660	0.73	0.55	0.62	0.76	0.10	0.27	0.12	0.09	0.14	0.16	0.23	0.13	0.03	0.02	0.02	0.03
19740	0.62	0.47	0.48	0.71	0.08	0.16	0.08	0.05	0.22	0.29	0.38	0.18	0.09	0.07	0.06	0.06
19780	0.71	0.65	0.66	0.83	0.10	0.14	0.11	0.06	0.11	0.13	0.16	0.07	0.08	0.08	0.07	0.05
19804	0.59	0.18	0.46	0.74	0.17	0.77	0.22	0.15	0.04	0.04	0.30	0.06	0.20	0.02	0.03	0.05
20020	0.71	0.52	0.68	0.75	0.22	0.42	0.25	0.19	0.04	0.04	0.05	0.04	0.02	0.01	0.02	0.02
20100	0.56	0.53	0.56	0.63	0.32	0.35	0.31	0.26	0.08	0.08	0.09	0.08	0.04	0.04	0.04	0.03
20220	0.85	0.81	0.86	0.90	0.08	0.12	0.08	0.05	0.04	0.04	0.04	0.03	0.04	0.03	0.03	0.02

20260	0.92	0.88	0.91	0.94	0.04	0.07	0.04	0.03	0.02	0.03	0.02	0.02	0.02	0.02	0.02	0.02
20500	0.55	0.40	0.43	0.63	0.21	0.40	0.32	0.20	0.11	0.16	0.20	0.11	0.13	0.05	0.05	0.06
20700	0.61	0.54	0.57	0.68	0.17	0.22	0.19	0.13	0.18	0.21	0.20	0.16	0.04	0.03	0.03	0.03
20740	0.89	0.89	0.90	0.92	0.02	0.04	0.03	0.02	0.03	0.03	0.03	0.03	0.06	0.04	0.04	0.04
20940	0.11	0.13	0.09	0.16	0.03	0.14	0.02	0.03	0.83	0.72	0.88	0.79	0.03	0.02	0.02	0.02
20994	0.62	0.48	0.40	0.70	0.07	0.14	0.08	0.06	0.22	0.33	0.48	0.19	0.08	0.05	0.04	0.05
21060	0.72	0.68	0.71	0.83	0.16	0.20	0.16	0.10	0.08	0.08	0.08	0.05	0.04	0.04	0.04	0.03
21140	0.72	0.56	0.59	0.77	0.07	0.15	0.10	0.06	0.19	0.28	0.30	0.16	0.02	0.02	0.02	0.02
21300	0.86	0.73	0.77	0.87	0.06	0.19	0.15	0.07	0.03	0.06	0.06	0.03	0.05	0.02	0.02	0.02
21340	0.21	0.18	0.10	0.20	0.06	0.07	0.03	0.05	0.69	0.72	0.86	0.72	0.04	0.03	0.02	0.03
21420	0.66	0.68	0.65	0.76	0.06	0.06	0.05	0.04	0.19	0.17	0.21	0.14	0.10	0.09	0.09	0.06
21500	0.74	0.60	0.68	0.87	0.14	0.26	0.20	0.07	0.06	0.10	0.08	0.04	0.05	0.05	0.04	0.03
21660	0.80	0.80	0.80	0.83	0.03	0.03	0.03	0.02	0.11	0.12	0.12	0.10	0.07	0.06	0.05	0.05
21780	0.84	0.72	0.80	0.87	0.09	0.21	0.14	0.08	0.03	0.05	0.04	0.03	0.04	0.02	0.02	0.02
21820	0.74	0.70	0.72	0.79	0.08	0.10	0.09	0.06	0.10	0.12	0.12	0.08	0.08	0.08	0.07	0.06
22020	0.80	0.78	0.82	0.86	0.11	0.12	0.09	0.07	0.04	0.04	0.05	0.04	0.06	0.05	0.04	0.03
22140	0.65	0.63	0.61	0.66	0.02	0.02	0.02	0.02	0.31	0.32	0.36	0.31	0.03	0.02	0.02	0.02
22180	0.44	0.39	0.46	0.52	0.37	0.45	0.35	0.31	0.14	0.13	0.15	0.13	0.05	0.04	0.04	0.04
22220	0.58	0.70	0.55	0.76	0.04	0.06	0.03	0.04	0.24	0.17	0.34	0.14	0.14	0.08	0.09	0.06
22380	0.71	0.69	0.68	0.75	0.03	0.04	0.03	0.03	0.20	0.22	0.25	0.19	0.05	0.05	0.04	0.04
22420	0.76	0.39	0.73	0.82	0.17	0.56	0.21	0.12	0.04	0.04	0.05	0.04	0.03	0.01	0.02	0.02
22500	0.59	0.42	0.56	0.60	0.33	0.54	0.39	0.35	0.04	0.03	0.04	0.03	0.04	0.01	0.02	0.02
22520	0.79	0.69	0.76	0.84	0.15	0.26	0.18	0.12	0.04	0.04	0.05	0.03	0.02	0.01	0.01	0.01
22540	0.85	0.83	0.84	0.89	0.04	0.06	0.04	0.03	0.08	0.09	0.09	0.06	0.02	0.02	0.02	0.02
22660	0.80	0.80	0.78	0.82	0.02	0.02	0.02	0.02	0.12	0.13	0.16	0.12	0.06	0.04	0.04	0.04
22744	0.36	0.20	0.32	0.48	0.22	0.50	0.24	0.17	0.36	0.26	0.39	0.30	0.06	0.03	0.05	0.05
22900	0.66	0.61	0.57	0.82	0.09	0.11	0.11	0.05	0.18	0.22	0.26	0.09	0.07	0.06	0.06	0.04
23060	0.55	0.51	0.57	0.81	0.21	0.27	0.22	0.09	0.10	0.14	0.15	0.06	0.14	0.09	0.06	0.04
23104	0.46	0.33	0.37	0.61	0.19	0.30	0.17	0.11	0.24	0.30	0.41	0.22	0.12	0.07	0.05	0.06
23224	0.42	0.35	0.34	0.59	0.18	0.28	0.22	0.14	0.18	0.22	0.30	0.14	0.22	0.15	0.14	0.14
23420	0.29	0.24	0.21	0.41	0.06	0.09	0.05	0.05	0.47	0.53	0.64	0.41	0.19	0.14	0.10	0.13
23460	0.78	0.48	0.63	0.85	0.16	0.43	0.27	0.10	0.04	0.08	0.08	0.04	0.02	0.01	0.01	0.01
23540	0.59	0.49	0.61	0.68	0.15	0.34	0.18	0.14	0.14	0.12	0.14	0.11	0.12	0.06	0.08	0.06
23580	0.59	0.48	0.42	0.71	0.08	0.14	0.10	0.06	0.28	0.35	0.46	0.20	0.04	0.03	0.03	0.03
23844	0.70	0.25	0.50	0.75	0.12	0.57	0.20	0.08	0.15	0.17	0.29	0.14	0.04	0.01	0.02	0.02
23900	0.87	0.86	0.85	0.89	0.03	0.04	0.03	0.02	0.08	0.08	0.11	0.07	0.02	0.02	0.01	0.01
24020	0.93	0.79	0.88	0.94	0.03	0.13	0.06	0.02	0.03	0.07	0.04	0.03	0.02	0.01	0.01	0.01
24140	0.54	0.42	0.49	0.60	0.32	0.46	0.27	0.26	0.11	0.11	0.21	0.12	0.03	0.02	0.02	0.02
24220	0.80	0.80	0.82	0.87	0.07	0.08	0.06	0.05	0.06	0.07	0.08	0.05	0.07	0.05	0.04	0.03
24260	0.68	0.60	0.54	0.76	0.04	0.06	0.04	0.03	0.27	0.33	0.41	0.20	0.02	0.02	0.01	0.01
24300	0.80	0.79	0.78	0.82	0.01	0.02	0.01	0.01	0.16	0.18	0.19	0.15	0.02	0.02	0.02	0.02
24340	0.71	0.56	0.59	0.83	0.11	0.24	0.12	0.06	0.12	0.15	0.25	0.08	0.07	0.05	0.04	0.03
24420	0.88	0.87	0.87	0.88	0.01	0.01	0.01	0.01	0.09	0.09	0.09	0.08	0.03	0.02	0.02	0.02
24500	0.86	0.83	0.85	0.90	0.04	0.04	0.04	0.02	0.07	0.08	0.08	0.05	0.03	0.04	0.03	0.02
24540	0.65	0.56	0.54	0.70	0.02	0.04	0.02	0.02	0.28	0.38	0.41	0.26	0.04	0.03	0.02	0.03
24580	0.77	0.70	0.67	0.87	0.05	0.10	0.06	0.03	0.13	0.16	0.22	0.07	0.05	0.04	0.05	0.03
24660	0.50	0.37	0.48	0.68	0.30	0.47	0.32	0.19	0.11	0.11	0.15	0.09	0.09	0.05	0.05	0.04
24780	0.54	0.44	0.48	0.58	0.36	0.46	0.38	0.32	0.06	0.08	0.12	0.07	0.03	0.02	0.02	0.03
24860	0.72	0.58	0.61	0.76	0.14	0.29	0.21	0.14	0.08	0.11	0.16	0.07	0.06	0.02	0.02	0.03
25060	0.64	0.49	0.60	0.74	0.22	0.41	0.27	0.17	0.07	0.08	0.09	0.06	0.06	0.03	0.04	0.03
25180	0.77	0.68	0.75	0.82	0.12	0.22	0.15	0.10	0.07	0.08	0.08	0.06	0.03	0.02	0.03	0.02
25220	0.60	0.51	0.62	0.66	0.32	0.42	0.30	0.27	0.06	0.05	0.07	0.06	0.02	0.01	0.01	0.01
25260	0.36	0.30	0.25	0.38	0.08	0.13	0.06	0.07	0.49	0.51	0.64	0.50	0.08	0.06	0.04	0.06
25420	0.67	0.49	0.54	0.81	0.13	0.30	0.24	0.08	0.08	0.15	0.16	0.06	0.12	0.07	0.06	0.06
25500	0.70	0.68	0.66	0.80	0.07	0.08	0.08	0.05	0.17	0.20	0.23	0.12	0.05	0.04	0.04	0.03

25540	0.65	0.37	0.43	0.76	0.10	0.34	0.19	0.07	0.13	0.24	0.33	0.10	0.12	0.05	0.05	0.06
25620	0.60	0.46	0.56	0.72	0.33	0.48	0.37	0.23	0.04	0.04	0.05	0.03	0.03	0.02	0.02	0.02
25860	0.76	0.71	0.71	0.82	0.08	0.14	0.11	0.07	0.10	0.12	0.14	0.08	0.06	0.04	0.04	0.03
25940	0.67	0.51	0.58	0.72	0.16	0.33	0.19	0.15	0.14	0.14	0.21	0.12	0.02	0.02	0.02	0.02
25980	0.38	0.36	0.40	0.47	0.45	0.48	0.41	0.38	0.13	0.12	0.15	0.12	0.04	0.04	0.04	0.03
26140	0.87	0.86	0.87	0.88	0.04	0.04	0.04	0.03	0.06	0.07	0.07	0.06	0.03	0.02	0.02	0.02
26300	0.80	0.66	0.75	0.83	0.11	0.22	0.14	0.08	0.07	0.10	0.10	0.07	0.02	0.02	0.02	0.02
26380	0.71	0.59	0.70	0.76	0.19	0.33	0.20	0.16	0.07	0.07	0.09	0.06	0.02	0.01	0.02	0.01
26420	0.33	0.22	0.25	0.51	0.17	0.34	0.17	0.12	0.27	0.36	0.51	0.28	0.22	0.08	0.06	0.09
26580	0.91	0.86	0.92	0.94	0.05	0.11	0.05	0.03	0.02	0.02	0.02	0.01	0.02	0.02	0.01	0.01
26620	0.67	0.47	0.57	0.73	0.21	0.42	0.28	0.17	0.06	0.08	0.11	0.06	0.06	0.03	0.03	0.04
26820	0.84	0.82	0.80	0.84	0.01	0.01	0.01	0.01	0.13	0.15	0.17	0.13	0.02	0.02	0.02	0.02
26900	0.68	0.42	0.52	0.79	0.12	0.41	0.27	0.10	0.07	0.14	0.18	0.06	0.13	0.03	0.04	0.05
26980	0.72	0.65	0.74	0.81	0.11	0.19	0.10	0.07	0.07	0.08	0.10	0.06	0.10	0.08	0.06	0.06
27060	0.64	0.73	0.71	0.77	0.06	0.08	0.06	0.06	0.08	0.08	0.08	0.07	0.22	0.12	0.14	0.10
27100	0.83	0.66	0.80	0.87	0.10	0.28	0.14	0.08	0.04	0.05	0.05	0.04	0.02	0.02	0.02	0.01
27140	0.61	0.28	0.46	0.64	0.31	0.68	0.46	0.31	0.03	0.03	0.06	0.03	0.04	0.01	0.02	0.02
27180	0.66	0.47	0.62	0.74	0.27	0.47	0.29	0.21	0.05	0.05	0.07	0.04	0.02	0.01	0.01	0.01
27260	0.60	0.39	0.58	0.70	0.19	0.46	0.22	0.15	0.12	0.10	0.14	0.10	0.09	0.04	0.06	0.05
27340	0.62	0.58	0.62	0.68	0.17	0.23	0.17	0.14	0.15	0.14	0.17	0.13	0.05	0.04	0.05	0.04
27500	0.80	0.66	0.70	0.84	0.07	0.14	0.12	0.06	0.10	0.18	0.16	0.08	0.03	0.02	0.02	0.02
27620	0.81	0.78	0.83	0.88	0.12	0.17	0.10	0.08	0.04	0.04	0.05	0.03	0.03	0.02	0.02	0.01
27740	0.87	0.83	0.86	0.90	0.06	0.09	0.06	0.04	0.05	0.06	0.06	0.04	0.03	0.02	0.02	0.02
27780	0.91	0.75	0.85	0.93	0.06	0.21	0.11	0.05	0.02	0.04	0.03	0.02	0.02	0.01	0.01	0.01
27860	0.68	0.63	0.68	0.79	0.22	0.27	0.22	0.14	0.07	0.08	0.07	0.05	0.03	0.02	0.02	0.02
27900	0.84	0.84	0.76	0.86	0.03	0.04	0.03	0.03	0.09	0.09	0.18	0.08	0.04	0.03	0.03	0.03
27980	0.25	0.36	0.33	0.44	0.01	0.02	0.02	0.02	0.10	0.11	0.11	0.11	0.64	0.51	0.54	0.43
28020	0.76	0.59	0.68	0.80	0.12	0.30	0.20	0.11	0.05	0.08	0.09	0.05	0.06	0.03	0.03	0.04
28100	0.76	0.44	0.58	0.78	0.12	0.38	0.24	0.10	0.10	0.17	0.17	0.10	0.02	0.01	0.01	0.02
28140	0.68	0.47	0.55	0.78	0.12	0.35	0.18	0.09	0.11	0.14	0.23	0.08	0.08	0.04	0.04	0.04
28420	0.64	0.58	0.45	0.66	0.02	0.03	0.02	0.02	0.27	0.34	0.50	0.27	0.06	0.05	0.03	0.05
28660	0.41	0.35	0.44	0.55	0.28	0.32	0.25	0.17	0.24	0.26	0.27	0.23	0.06	0.06	0.05	0.04
28700	0.93	0.90	0.93	0.94	0.03	0.06	0.04	0.03	0.02	0.03	0.02	0.02	0.02	0.01	0.01	0.01
28740	0.76	0.70	0.71	0.79	0.08	0.12	0.10	0.07	0.12	0.16	0.16	0.11	0.04	0.03	0.03	0.03
28940	0.83	0.68	0.78	0.87	0.07	0.23	0.10	0.06	0.06	0.08	0.09	0.05	0.05	0.02	0.03	0.02
29020	0.84	0.76	0.82	0.86	0.10	0.18	0.12	0.09	0.04	0.05	0.04	0.04	0.03	0.02	0.02	0.02
29100	0.87	0.86	0.88	0.90	0.03	0.04	0.03	0.02	0.03	0.03	0.03	0.02	0.07	0.06	0.05	0.05
29180	0.67	0.49	0.66	0.73	0.22	0.44	0.24	0.20	0.06	0.05	0.07	0.05	0.05	0.02	0.03	0.02
29200	0.63	0.68	0.72	0.79	0.06	0.12	0.08	0.06	0.08	0.12	0.13	0.09	0.24	0.08	0.07	0.07
29340	0.68	0.38	0.66	0.76	0.21	0.55	0.25	0.16	0.06	0.05	0.06	0.05	0.04	0.02	0.03	0.02
29404	0.62	0.41	0.40	0.71	0.05	0.18	0.12	0.05	0.15	0.36	0.42	0.15	0.17	0.06	0.06	0.08
29420	0.79	0.77	0.76	0.80	0.02	0.02	0.02	0.02	0.17	0.18	0.20	0.16	0.03	0.02	0.02	0.02
29460	0.58	0.46	0.49	0.61	0.14	0.25	0.16	0.13	0.25	0.27	0.33	0.23	0.04	0.02	0.02	0.02
29540	0.75	0.63	0.60	0.85	0.06	0.09	0.10	0.04	0.14	0.24	0.26	0.08	0.05	0.04	0.04	0.03
29620	0.66	0.60	0.69	0.82	0.14	0.23	0.16	0.08	0.07	0.11	0.10	0.06	0.14	0.06	0.05	0.04
29700	0.05	0.06	0.04	0.05	0.01	0.01	0.00	0.00	0.94	0.92	0.96	0.93	0.01	0.01	0.00	0.01
29740	0.39	0.35	0.23	0.37	0.03	0.03	0.02	0.02	0.55	0.59	0.74	0.58	0.04	0.03	0.01	0.02
29820	0.42	0.34	0.31	0.49	0.14	0.20	0.15	0.12	0.26	0.34	0.42	0.25	0.19	0.13	0.11	0.14
29940	0.78	0.77	0.78	0.81	0.07	0.08	0.08	0.06	0.08	0.08	0.08	0.07	0.07	0.06	0.06	0.05
30020	0.56	0.53	0.57	0.63	0.22	0.26	0.22	0.18	0.16	0.16	0.16	0.14	0.06	0.05	0.05	0.05
30140	0.83	0.72	0.60	0.85	0.03	0.04	0.04	0.02	0.12	0.22	0.35	0.11	0.03	0.02	0.02	0.02
30300	0.92	0.92	0.92	0.93	0.01	0.01	0.01	0.01	0.04	0.05	0.05	0.04	0.02	0.02	0.02	0.02
30340	0.88	0.71	0.86	0.91	0.08	0.24	0.10	0.06	0.02	0.03	0.02	0.02	0.02	0.02	0.02	0.01
30460	0.74	0.61	0.64	0.79	0.12	0.24	0.19	0.11	0.06	0.12	0.14	0.07	0.08	0.03	0.03	0.04
30620	0.81	0.64	0.77	0.83	0.13	0.31	0.18	0.12	0.03	0.04	0.04	0.03	0.02	0.01	0.01	0.01

30700	0.75	0.74	0.74	0.83	0.07	0.09	0.08	0.05	0.09	0.11	0.12	0.07	0.08	0.06	0.06	0.05
30780	0.66	0.41	0.50	0.75	0.22	0.47	0.34	0.16	0.06	0.10	0.14	0.06	0.05	0.02	0.02	0.03
30860	0.82	0.79	0.80	0.85	0.02	0.02	0.02	0.01	0.13	0.15	0.15	0.10	0.04	0.04	0.04	0.03
30980	0.64	0.51	0.53	0.68	0.19	0.27	0.23	0.16	0.15	0.20	0.23	0.15	0.02	0.01	0.01	0.01
31020	0.83	0.81	0.81	0.85	0.02	0.02	0.02	0.02	0.11	0.13	0.14	0.10	0.04	0.04	0.04	0.03
31084	0.26	0.18	0.15	0.48	0.06	0.24	0.08	0.06	0.35	0.46	0.65	0.29	0.33	0.11	0.12	0.17
31140	0.70	0.48	0.64	0.80	0.16	0.41	0.20	0.11	0.07	0.08	0.13	0.06	0.07	0.03	0.04	0.03
31180	0.48	0.33	0.44	0.59	0.16	0.25	0.09	0.06	0.27	0.36	0.44	0.32	0.10	0.06	0.03	0.03
31340	0.75	0.64	0.73	0.79	0.17	0.31	0.20	0.16	0.04	0.04	0.04	0.03	0.03	0.02	0.02	0.02
31420	0.55	0.32	0.43	0.63	0.37	0.63	0.48	0.31	0.04	0.04	0.07	0.03	0.04	0.02	0.02	0.02
31460	0.35	0.28	0.21	0.52	0.04	0.09	0.03	0.03	0.56	0.60	0.73	0.41	0.05	0.03	0.03	0.04
31540	0.71	0.68	0.69	0.83	0.07	0.12	0.10	0.05	0.08	0.12	0.13	0.06	0.14	0.08	0.08	0.06
31700	0.79	0.75	0.73	0.85	0.04	0.06	0.06	0.03	0.08	0.13	0.16	0.07	0.09	0.06	0.05	0.05
31740	0.68	0.62	0.66	0.75	0.12	0.17	0.14	0.09	0.12	0.15	0.14	0.10	0.07	0.06	0.06	0.05
31860	0.81	0.81	0.82	0.86	0.09	0.09	0.08	0.06	0.06	0.06	0.06	0.05	0.05	0.04	0.04	0.03
31900	0.87	0.67	0.82	0.88	0.09	0.29	0.15	0.09	0.02	0.03	0.02	0.02	0.02	0.01	0.01	0.01
32580	0.09	0.08	0.06	0.11	0.01	0.02	0.00	0.01	0.86	0.88	0.93	0.86	0.04	0.02	0.01	0.02
32780	0.80	0.79	0.76	0.82	0.02	0.02	0.02	0.02	0.14	0.16	0.19	0.14	0.04	0.04	0.03	0.03
32820	0.54	0.23	0.34	0.63	0.31	0.68	0.45	0.27	0.07	0.07	0.18	0.06	0.08	0.02	0.03	0.04
32900	0.23	0.25	0.22	0.33	0.04	0.06	0.04	0.04	0.59	0.60	0.67	0.56	0.14	0.09	0.08	0.08
33124	0.20	0.08	0.12	0.28	0.12	0.49	0.09	0.08	0.65	0.42	0.78	0.61	0.03	0.01	0.02	0.03
33140	0.80	0.60	0.76	0.82	0.12	0.31	0.13	0.10	0.07	0.08	0.09	0.07	0.01	0.01	0.01	0.01
33220	0.89	0.90	0.91	0.92	0.03	0.03	0.02	0.02	0.04	0.04	0.04	0.03	0.05	0.04	0.03	0.03
33260	0.52	0.36	0.38	0.55	0.07	0.12	0.08	0.05	0.37	0.50	0.52	0.36	0.04	0.03	0.02	0.03
33340	0.60	0.24	0.46	0.80	0.20	0.61	0.13	0.07	0.12	0.09	0.36	0.08	0.09	0.06	0.05	0.05
33460	0.61	0.55	0.62	0.79	0.15	0.24	0.16	0.08	0.08	0.10	0.12	0.06	0.17	0.12	0.10	0.07
33540	0.90	0.90	0.82	0.91	0.02	0.02	0.01	0.01	0.05	0.06	0.14	0.05	0.03	0.03	0.02	0.02
33660	0.63	0.36	0.60	0.70	0.27	0.60	0.32	0.24	0.04	0.03	0.04	0.04	0.06	0.02	0.03	0.03
33700	0.38	0.37	0.33	0.46	0.04	0.05	0.03	0.03	0.45	0.48	0.56	0.42	0.12	0.10	0.08	0.08
33740	0.56	0.31	0.57	0.74	0.36	0.65	0.35	0.21	0.04	0.03	0.06	0.04	0.03	0.01	0.02	0.02
33780	0.90	0.84	0.89	0.91	0.05	0.09	0.05	0.04	0.04	0.05	0.05	0.04	0.01	0.01	0.01	0.01
33860	0.48	0.31	0.43	0.64	0.38	0.62	0.46	0.30	0.04	0.04	0.08	0.04	0.11	0.02	0.03	0.03
33874	0.73	0.62	0.67	0.80	0.07	0.20	0.12	0.06	0.06	0.11	0.14	0.06	0.14	0.07	0.07	0.07
34060	0.82	0.75	0.81	0.89	0.07	0.15	0.10	0.05	0.04	0.05	0.04	0.03	0.08	0.04	0.05	0.03
34100	0.82	0.78	0.73	0.88	0.04	0.05	0.05	0.03	0.12	0.15	0.20	0.08	0.02	0.02	0.02	0.01
34580	0.73	0.72	0.63	0.78	0.01	0.02	0.02	0.01	0.21	0.22	0.31	0.16	0.05	0.04	0.04	0.04
34620	0.84	0.71	0.83	0.87	0.08	0.24	0.11	0.08	0.04	0.04	0.04	0.03	0.03	0.02	0.02	0.02
34740	0.82	0.43	0.71	0.84	0.11	0.48	0.20	0.09	0.06	0.08	0.07	0.06	0.02	0.01	0.01	0.01
34820	0.76	0.70	0.73	0.82	0.13	0.20	0.14	0.11	0.08	0.08	0.11	0.06	0.03	0.02	0.02	0.02
34900	0.34	0.38	0.47	0.58	0.06	0.06	0.02	0.02	0.32	0.32	0.43	0.33	0.28	0.24	0.08	0.07
34940	0.70	0.41	0.42	0.75	0.05	0.14	0.11	0.04	0.22	0.44	0.46	0.18	0.03	0.01	0.02	0.02
34980	0.66	0.51	0.56	0.77	0.15	0.33	0.21	0.12	0.11	0.13	0.19	0.08	0.08	0.04	0.04	0.04
35004	0.57	0.31	0.43	0.72	0.08	0.30	0.15	0.05	0.15	0.32	0.35	0.15	0.20	0.07	0.06	0.08
35084	0.57	0.16	0.29	0.70	0.10	0.57	0.23	0.07	0.16	0.24	0.43	0.14	0.17	0.03	0.05	0.09
35100	0.60	0.60	0.64	0.70	0.24	0.30	0.22	0.20	0.08	0.07	0.09	0.06	0.08	0.04	0.04	0.03
35154	0.43	0.43	0.45	0.72	0.09	0.18	0.10	0.05	0.12	0.22	0.34	0.12	0.36	0.18	0.11	0.10
35300	0.62	0.37	0.41	0.72	0.13	0.30	0.20	0.09	0.16	0.28	0.34	0.14	0.09	0.04	0.04	0.05
35380	0.47	0.27	0.48	0.66	0.30	0.61	0.29	0.19	0.14	0.10	0.18	0.12	0.09	0.03	0.04	0.03
35614	0.34	0.15	0.23	0.59	0.10	0.49	0.18	0.08	0.23	0.28	0.47	0.18	0.34	0.08	0.12	0.15
35660	0.73	0.37	0.70	0.84	0.12	0.54	0.19	0.08	0.07	0.07	0.08	0.06	0.07	0.02	0.03	0.03
35840	0.78	0.56	0.62	0.81	0.06	0.18	0.11	0.05	0.12	0.23	0.24	0.11	0.04	0.02	0.02	0.03
35980	0.70	0.59	0.58	0.80	0.09	0.14	0.14	0.06	0.13	0.21	0.22	0.09	0.08	0.06	0.06	0.05
36084	0.28	0.25	0.25	0.47	0.08	0.20	0.13	0.08	0.20	0.31	0.38	0.19	0.44	0.23	0.24	0.26
36100	0.67	0.56	0.61	0.74	0.12	0.23	0.16	0.10	0.17	0.19	0.21	0.13	0.04	0.02	0.02	0.02
36140	0.84	0.75	0.76	0.88	0.06	0.10	0.08	0.04	0.09	0.13	0.14	0.07	0.02	0.02	0.02	0.01

36220	0.40	0.30	0.28	0.39	0.06	0.09	0.05	0.05	0.51	0.59	0.66	0.54	0.03	0.02	0.02	0.02
36260	0.79	0.75	0.73	0.82	0.02	0.03	0.02	0.02	0.14	0.18	0.21	0.12	0.04	0.04	0.04	0.04
36420	0.66	0.50	0.50	0.72	0.13	0.30	0.13	0.11	0.13	0.16	0.34	0.13	0.08	0.04	0.04	0.05
36500	0.70	0.69	0.72	0.76	0.06	0.07	0.06	0.05	0.11	0.12	0.12	0.10	0.13	0.12	0.11	0.10
36540	0.68	0.52	0.56	0.80	0.14	0.27	0.11	0.07	0.09	0.14	0.29	0.09	0.08	0.07	0.04	0.04
36740	0.46	0.32	0.36	0.56	0.14	0.33	0.15	0.12	0.32	0.30	0.44	0.26	0.08	0.05	0.05	0.06
36780	0.84	0.82	0.84	0.88	0.05	0.07	0.05	0.04	0.06	0.06	0.07	0.05	0.05	0.05	0.04	0.04
36980	0.83	0.81	0.84	0.89	0.07	0.11	0.08	0.06	0.04	0.05	0.05	0.04	0.05	0.03	0.03	0.02
37100	0.48	0.44	0.29	0.57	0.03	0.03	0.02	0.02	0.36	0.42	0.61	0.29	0.13	0.11	0.08	0.11
37340	0.74	0.61	0.68	0.76	0.10	0.21	0.14	0.09	0.12	0.14	0.14	0.11	0.05	0.03	0.04	0.04
37460	0.74	0.63	0.72	0.79	0.12	0.24	0.15	0.10	0.08	0.10	0.10	0.08	0.04	0.04	0.04	0.04
37620	0.94	0.94	0.94	0.95	0.03	0.04	0.03	0.02	0.02	0.02	0.02	0.01	0.02	0.01	0.01	0.01
37860	0.70	0.56	0.70	0.75	0.17	0.34	0.18	0.14	0.07	0.07	0.08	0.06	0.05	0.04	0.05	0.04
37900	0.73	0.51	0.68	0.87	0.11	0.39	0.22	0.07	0.04	0.08	0.07	0.03	0.12	0.03	0.03	0.03
37964	0.47	0.18	0.28	0.67	0.23	0.66	0.31	0.16	0.12	0.10	0.33	0.08	0.18	0.05	0.09	0.10
38060	0.58	0.45	0.40	0.66	0.07	0.11	0.08	0.06	0.25	0.38	0.48	0.22	0.10	0.06	0.05	0.06
38220	0.54	0.26	0.55	0.67	0.40	0.71	0.40	0.28	0.03	0.02	0.04	0.03	0.03	0.01	0.01	0.01
38300	0.77	0.57	0.80	0.88	0.08	0.38	0.13	0.07	0.03	0.03	0.03	0.02	0.12	0.03	0.04	0.03
38340	0.85	0.79	0.82	0.88	0.05	0.10	0.07	0.04	0.06	0.08	0.08	0.05	0.04	0.02	0.02	0.02
38540	0.84	0.83	0.78	0.85	0.02	0.02	0.01	0.01	0.10	0.12	0.18	0.11	0.04	0.03	0.02	0.03
38860	0.88	0.82	0.89	0.92	0.05	0.10	0.05	0.03	0.03	0.04	0.03	0.02	0.04	0.04	0.03	0.03
38900	0.66	0.65	0.65	0.74	0.05	0.08	0.05	0.04	0.13	0.16	0.20	0.13	0.16	0.12	0.10	0.10
38940	0.63	0.44	0.53	0.70	0.15	0.32	0.19	0.12	0.19	0.22	0.26	0.16	0.03	0.02	0.02	0.02
39100	0.64	0.48	0.53	0.70	0.12	0.22	0.16	0.09	0.19	0.26	0.27	0.16	0.06	0.04	0.04	0.04
39150	0.82	0.80	0.77	0.82	0.01	0.01	0.01	0.01	0.15	0.16	0.20	0.14	0.03	0.02	0.02	0.02
39300	0.70	0.57	0.47	0.81	0.07	0.14	0.13	0.06	0.15	0.25	0.36	0.09	0.07	0.04	0.04	0.04
39340	0.78	0.78	0.75	0.82	0.01	0.01	0.01	0.01	0.14	0.15	0.19	0.13	0.06	0.05	0.05	0.04
39380	0.56	0.49	0.48	0.57	0.03	0.04	0.03	0.02	0.39	0.45	0.48	0.39	0.02	0.02	0.01	0.02
39460	0.82	0.78	0.80	0.85	0.07	0.10	0.08	0.05	0.09	0.10	0.10	0.07	0.02	0.02	0.02	0.02
39540	0.73	0.51	0.56	0.77	0.12	0.25	0.21	0.10	0.12	0.23	0.22	0.12	0.02	0.02	0.02	0.02
39580	0.52	0.48	0.52	0.66	0.14	0.31	0.25	0.16	0.09	0.16	0.17	0.11	0.25	0.06	0.06	0.07
39660	0.87	0.86	0.87	0.90	0.04	0.04	0.03	0.02	0.06	0.07	0.06	0.05	0.03	0.04	0.03	0.02
39740	0.73	0.54	0.36	0.81	0.06	0.08	0.08	0.04	0.18	0.36	0.54	0.12	0.03	0.02	0.01	0.02
39820	0.79	0.79	0.80	0.82	0.02	0.03	0.02	0.02	0.12	0.12	0.13	0.12	0.06	0.06	0.05	0.05
39900	0.60	0.55	0.51	0.67	0.04	0.05	0.04	0.03	0.26	0.31	0.36	0.22	0.10	0.09	0.08	0.08
40060	0.57	0.38	0.44	0.68	0.20	0.49	0.33	0.20	0.08	0.09	0.17	0.06	0.15	0.04	0.05	0.05
40140	0.31	0.26	0.23	0.43	0.09	0.12	0.08	0.07	0.43	0.52	0.61	0.41	0.18	0.10	0.07	0.09
40220	0.75	0.53	0.67	0.83	0.14	0.37	0.21	0.10	0.06	0.07	0.09	0.04	0.05	0.03	0.03	0.03
40340	0.76	0.72	0.77	0.85	0.09	0.12	0.09	0.05	0.06	0.07	0.08	0.05	0.10	0.08	0.07	0.05
40380	0.68	0.42	0.52	0.83	0.14	0.37	0.26	0.07	0.08	0.17	0.18	0.06	0.10	0.04	0.04	0.04
40420	0.67	0.47	0.57	0.73	0.12	0.29	0.17	0.10	0.15	0.20	0.23	0.14	0.05	0.03	0.03	0.03
40484	0.89	0.90	0.90	0.92	0.02	0.02	0.02	0.02	0.04	0.04	0.04	0.03	0.05	0.04	0.04	0.03
40580	0.44	0.36	0.49	0.54	0.48	0.57	0.38	0.37	0.06	0.06	0.12	0.08	0.02	0.01	0.01	0.01
40660	0.68	0.56	0.54	0.76	0.16	0.27	0.21	0.13	0.14	0.16	0.23	0.09	0.03	0.02	0.02	0.02
40900	0.38	0.36	0.42	0.60	0.11	0.14	0.10	0.06	0.23	0.26	0.29	0.20	0.28	0.24	0.18	0.14
40980	0.74	0.38	0.59	0.80	0.14	0.49	0.28	0.11	0.08	0.13	0.12	0.08	0.04	0.01	0.02	0.02
41060	0.78	0.64	0.80	0.87	0.14	0.27	0.10	0.07	0.04	0.05	0.08	0.04	0.04	0.04	0.02	0.02
41100	0.83	0.82	0.80	0.85	0.01	0.01	0.01	0.01	0.12	0.14	0.16	0.11	0.04	0.04	0.03	0.03
41140	0.82	0.81	0.83	0.87	0.08	0.10	0.08	0.06	0.06	0.07	0.08	0.05	0.04	0.02	0.02	0.02
41180	0.73	0.35	0.69	0.83	0.14	0.59	0.19	0.10	0.04	0.04	0.07	0.04	0.09	0.03	0.04	0.04
41420	0.66	0.66	0.55	0.72	0.02	0.02	0.02	0.02	0.27	0.26	0.38	0.22	0.06	0.05	0.05	0.04
41500	0.35	0.32	0.15	0.52	0.04	0.10	0.02	0.03	0.48	0.46	0.76	0.34	0.13	0.12	0.06	0.10
41540	0.70	0.54	0.60	0.76	0.19	0.34	0.20	0.14	0.08	0.10	0.18	0.08	0.03	0.02	0.02	0.02
41620	0.65	0.62	0.58	0.74	0.03	0.04	0.03	0.02	0.22	0.25	0.30	0.16	0.10	0.09	0.09	0.07
41660	0.56	0.49	0.46	0.59	0.05	0.06	0.04	0.04	0.36	0.42	0.47	0.35	0.03	0.02	0.02	0.02

41700	0.37	0.29	0.26	0.46	0.09	0.14	0.07	0.07	0.46	0.52	0.64	0.43	0.08	0.05	0.03	0.04
41740	0.39	0.36	0.32	0.56	0.06	0.10	0.06	0.05	0.29	0.38	0.49	0.25	0.26	0.16	0.13	0.14
41884	0.33	0.30	0.30	0.49	0.04	0.12	0.05	0.04	0.18	0.23	0.33	0.16	0.45	0.34	0.32	0.31
41940	0.25	0.29	0.24	0.40	0.03	0.04	0.03	0.03	0.20	0.29	0.42	0.22	0.52	0.38	0.32	0.36
42020	0.68	0.53	0.62	0.70	0.02	0.14	0.02	0.02	0.23	0.28	0.31	0.23	0.08	0.05	0.05	0.06
42034	0.68	0.55	0.52	0.73	0.04	0.14	0.04	0.03	0.18	0.22	0.36	0.15	0.11	0.10	0.09	0.09
42100	0.58	0.56	0.35	0.68	0.03	0.03	0.02	0.02	0.30	0.31	0.58	0.23	0.09	0.10	0.06	0.07
42140	0.49	0.46	0.36	0.57	0.01	0.02	0.01	0.01	0.46	0.49	0.61	0.39	0.03	0.02	0.02	0.02
42200	0.43	0.42	0.31	0.55	0.02	0.04	0.02	0.02	0.38	0.45	0.61	0.35	0.16	0.09	0.06	0.08
42220	0.58	0.56	0.52	0.66	0.03	0.03	0.03	0.02	0.30	0.33	0.39	0.26	0.09	0.08	0.07	0.07
42340	0.54	0.39	0.51	0.66	0.31	0.50	0.33	0.23	0.09	0.08	0.11	0.07	0.06	0.04	0.05	0.04
42540	0.76	0.69	0.57	0.85	0.07	0.11	0.07	0.04	0.11	0.16	0.34	0.08	0.06	0.03	0.02	0.02
42644	0.51	0.47	0.54	0.64	0.08	0.14	0.09	0.06	0.11	0.14	0.15	0.10	0.30	0.24	0.22	0.20
42680	0.77	0.53	0.64	0.80	0.08	0.32	0.09	0.07	0.12	0.13	0.24	0.11	0.02	0.02	0.02	0.02
42700	0.64	0.55	0.60	0.70	0.10	0.19	0.12	0.09	0.22	0.24	0.26	0.19	0.04	0.02	0.02	0.02
43100	0.75	0.76	0.75	0.85	0.04	0.05	0.04	0.03	0.11	0.10	0.11	0.07	0.11	0.09	0.09	0.06
43300	0.71	0.64	0.65	0.77	0.09	0.11	0.10	0.06	0.16	0.22	0.23	0.14	0.04	0.02	0.02	0.02
43340	0.61	0.31	0.53	0.66	0.29	0.63	0.36	0.26	0.06	0.04	0.08	0.05	0.04	0.01	0.02	0.02
43420	0.60	0.59	0.40	0.66	0.08	0.09	0.04	0.05	0.26	0.27	0.54	0.25	0.06	0.06	0.03	0.04
43580	0.60	0.54	0.52	0.78	0.08	0.12	0.09	0.05	0.27	0.29	0.34	0.15	0.05	0.05	0.05	0.03
43620	0.78	0.76	0.79	0.87	0.10	0.11	0.09	0.05	0.07	0.08	0.09	0.05	0.06	0.05	0.04	0.02
43780	0.75	0.55	0.56	0.79	0.11	0.29	0.22	0.11	0.07	0.14	0.20	0.07	0.06	0.02	0.02	0.03
43900	0.64	0.53	0.58	0.72	0.22	0.34	0.24	0.17	0.09	0.10	0.14	0.08	0.05	0.03	0.03	0.03
44060	0.82	0.82	0.83	0.86	0.04	0.05	0.04	0.03	0.07	0.08	0.08	0.06	0.06	0.06	0.05	0.05
44100	0.79	0.59	0.75	0.84	0.13	0.35	0.18	0.11	0.03	0.03	0.03	0.02	0.05	0.02	0.03	0.03
44140	0.67	0.44	0.41	0.79	0.08	0.17	0.13	0.05	0.16	0.35	0.43	0.12	0.10	0.04	0.03	0.04
44180	0.86	0.84	0.87	0.90	0.05	0.07	0.05	0.04	0.05	0.06	0.05	0.04	0.04	0.03	0.03	0.02
44220	0.85	0.63	0.78	0.86	0.10	0.31	0.15	0.09	0.04	0.05	0.06	0.04	0.02	0.01	0.01	0.01
44300	0.72	0.67	0.77	0.86	0.04	0.18	0.07	0.03	0.05	0.06	0.05	0.03	0.19	0.09	0.11	0.07
44420	0.83	0.79	0.81	0.86	0.10	0.13	0.11	0.08	0.05	0.06	0.07	0.04	0.02	0.02	0.01	0.01
44700	0.23	0.22	0.25	0.39	0.10	0.13	0.09	0.07	0.36	0.42	0.49	0.38	0.32	0.23	0.17	0.16
44940	0.51	0.38	0.46	0.54	0.41	0.56	0.47	0.40	0.05	0.04	0.05	0.04	0.03	0.02	0.02	0.02
45060	0.68	0.50	0.65	0.86	0.16	0.34	0.21	0.07	0.06	0.10	0.09	0.04	0.10	0.06	0.05	0.04
45104	0.58	0.55	0.58	0.69	0.12	0.15	0.12	0.09	0.14	0.15	0.16	0.11	0.15	0.15	0.14	0.11
45220	0.59	0.40	0.51	0.64	0.25	0.49	0.35	0.24	0.08	0.08	0.10	0.08	0.07	0.03	0.04	0.04
45300	0.58	0.43	0.50	0.69	0.12	0.29	0.14	0.09	0.21	0.23	0.31	0.17	0.08	0.05	0.05	0.05
45460	0.86	0.79	0.85	0.91	0.07	0.15	0.10	0.05	0.03	0.04	0.03	0.02	0.04	0.02	0.02	0.02
45500	0.67	0.50	0.58	0.71	0.24	0.41	0.32	0.21	0.07	0.08	0.09	0.06	0.03	0.01	0.01	0.01
45540	0.87	0.56	0.72	0.89	0.05	0.31	0.16	0.05	0.06	0.13	0.12	0.05	0.02	0.01	0.01	0.01
45780	0.76	0.48	0.71	0.81	0.14	0.42	0.18	0.10	0.06	0.08	0.10	0.07	0.04	0.02	0.02	0.02
45820	0.77	0.63	0.63	0.82	0.09	0.16	0.14	0.07	0.11	0.19	0.21	0.10	0.03	0.02	0.02	0.02
45940	0.47	0.26	0.28	0.59	0.09	0.42	0.24	0.12	0.11	0.26	0.41	0.14	0.34	0.06	0.07	0.14
46060	0.59	0.51	0.40	0.63	0.05	0.07	0.05	0.04	0.30	0.38	0.52	0.28	0.06	0.05	0.03	0.05
46140	0.65	0.47	0.53	0.76	0.12	0.31	0.15	0.08	0.14	0.17	0.28	0.11	0.09	0.04	0.04	0.04
46220	0.64	0.39	0.59	0.69	0.27	0.55	0.30	0.24	0.06	0.04	0.10	0.05	0.04	0.01	0.02	0.02
46300	0.76	0.75	0.69	0.76	0.02	0.02	0.01	0.01	0.19	0.20	0.28	0.20	0.03	0.03	0.02	0.02
46340	0.66	0.43	0.42	0.69	0.15	0.28	0.23	0.13	0.14	0.26	0.34	0.15	0.04	0.02	0.02	0.03
46520	0.14	0.25	0.21	0.29	0.03	0.06	0.05	0.05	0.08	0.13	0.12	0.11	0.76	0.56	0.63	0.55
46540	0.53	0.56	0.63	0.88	0.17	0.19	0.16	0.05	0.13	0.13	0.12	0.04	0.17	0.11	0.10	0.03
46660	0.57	0.40	0.54	0.62	0.33	0.52	0.34	0.28	0.07	0.07	0.10	0.07	0.03	0.02	0.02	0.02
46700	0.31	0.28	0.32	0.43	0.17	0.21	0.17	0.13	0.26	0.30	0.34	0.26	0.26	0.21	0.18	0.17
47020	0.49	0.37	0.38	0.53	0.06	0.08	0.07	0.05	0.42	0.54	0.53	0.40	0.03	0.02	0.02	0.02
47220	0.50	0.35	0.29	0.58	0.16	0.29	0.19	0.16	0.31	0.35	0.50	0.24	0.03	0.01	0.01	0.02
47260	0.54	0.38	0.50	0.64	0.28	0.50	0.33	0.23	0.09	0.08	0.10	0.07	0.09	0.05	0.06	0.06
47300	0.29	0.32	0.23	0.37	0.02	0.02	0.01	0.02	0.63	0.62	0.72	0.57	0.06	0.04	0.04	0.04

47380	0.60	0.40	0.44	0.64	0.13	0.28	0.18	0.12	0.21	0.30	0.37	0.21	0.06	0.02	0.02	0.03
47460	0.72	0.66	0.66	0.72	0.03	0.08	0.02	0.02	0.22	0.22	0.29	0.22	0.04	0.04	0.03	0.03
47580	0.55	0.45	0.49	0.58	0.31	0.44	0.38	0.31	0.07	0.08	0.10	0.07	0.06	0.03	0.04	0.04
47664	0.67	0.50	0.71	0.82	0.11	0.39	0.15	0.08	0.04	0.05	0.08	0.04	0.18	0.06	0.06	0.06
47894	0.45	0.23	0.33	0.58	0.16	0.55	0.26	0.16	0.16	0.15	0.30	0.14	0.22	0.07	0.12	0.13
47940	0.74	0.59	0.70	0.86	0.14	0.29	0.18	0.07	0.06	0.08	0.08	0.04	0.06	0.05	0.04	0.03
48060	0.74	0.72	0.73	0.85	0.11	0.12	0.12	0.06	0.11	0.11	0.11	0.06	0.04	0.04	0.04	0.03
48140	0.82	0.84	0.86	0.90	0.02	0.02	0.02	0.01	0.04	0.04	0.05	0.03	0.12	0.09	0.07	0.05
48260	0.89	0.81	0.88	0.92	0.07	0.15	0.09	0.05	0.02	0.03	0.02	0.02	0.01	0.01	0.01	0.01
48300	0.68	0.66	0.59	0.69	0.01	0.01	0.01	0.01	0.29	0.31	0.38	0.28	0.02	0.02	0.02	0.02
48424	0.57	0.33	0.41	0.66	0.16	0.40	0.19	0.12	0.22	0.24	0.36	0.19	0.05	0.03	0.03	0.04
48540	0.92	0.87	0.92	0.94	0.05	0.11	0.05	0.04	0.02	0.02	0.02	0.01	0.01	0.01	0.01	0.01
48620	0.62	0.51	0.58	0.77	0.13	0.24	0.12	0.07	0.15	0.18	0.25	0.12	0.10	0.06	0.05	0.04
48660	0.67	0.54	0.59	0.72	0.11	0.22	0.13	0.09	0.18	0.22	0.26	0.17	0.04	0.03	0.03	0.03
48700	0.87	0.76	0.83	0.91	0.09	0.18	0.12	0.06	0.03	0.04	0.03	0.02	0.02	0.02	0.02	0.01
48864	0.60	0.42	0.50	0.68	0.21	0.41	0.28	0.17	0.08	0.12	0.17	0.09	0.11	0.05	0.05	0.06
48900	0.79	0.60	0.70	0.80	0.11	0.29	0.16	0.11	0.08	0.09	0.12	0.07	0.03	0.02	0.02	0.02
49020	0.77	0.70	0.69	0.82	0.07	0.10	0.10	0.06	0.13	0.17	0.19	0.10	0.03	0.03	0.03	0.02
49180	0.66	0.43	0.51	0.76	0.18	0.38	0.27	0.12	0.11	0.17	0.19	0.09	0.04	0.02	0.02	0.02
49340	0.65	0.59	0.56	0.80	0.07	0.12	0.10	0.05	0.14	0.22	0.28	0.10	0.15	0.07	0.06	0.05
49420	0.54	0.47	0.31	0.58	0.01	0.02	0.01	0.01	0.42	0.49	0.67	0.38	0.03	0.02	0.02	0.02
49620	0.82	0.64	0.60	0.86	0.07	0.15	0.16	0.06	0.08	0.19	0.22	0.06	0.03	0.02	0.02	0.02
49660	0.84	0.54	0.64	0.88	0.11	0.37	0.26	0.08	0.04	0.08	0.09	0.03	0.02	0.01	0.01	0.01
49700	0.43	0.47	0.45	0.52	0.04	0.06	0.04	0.04	0.30	0.33	0.36	0.30	0.24	0.15	0.15	0.14
49740	0.39	0.35	0.21	0.49	0.03	0.05	0.02	0.02	0.55	0.59	0.75	0.46	0.03	0.02	0.02	0.02
